# Life-Cycle Risk Assessment of Second-Generation Cellulose Nanomaterials

**DOI:** 10.3390/nano15030238

**Published:** 2025-02-04

**Authors:** James D. Ede, Amanda K. Charlton-Sevcik, Julia Griffin, Padmapriya Srinivasan, Yueyang Zhang, Christie M. Sayes, You-Lo Hsieh, Nicole Stark, Jo Anne Shatkin

**Affiliations:** 1 Vireo Advisors, LLC, Boston, MA 02205, USA; 2 Department of Environmental Science, Baylor University, Waco, TX 76798, USA; 3 Department of Chemistry, University of British Columbia, Vancouver, BC V6T 1Z4, Canada; 4 Department of Biological Sciences, University of Alberta, Edmonton, AB T6G 2E9, Canada; 5 Biological and Agricultural Engineering, Chemical Engineering, University of California at Davis, Davis, CA 95616, USA; 6 Forest Products Laboratory (FPL), USDA Forest Service, Madison, WI 53726, USA

**Keywords:** nanomaterial, cellulose, risk assessment, life-cycle, hazard, exposure

## Abstract

A nanomaterial life-cycle risk assessment (Nano LCRA) was conducted for second-generation functionalized cellulose nanomaterials (CNs) in five case studies, including applications in water filtration, food contact packaging (including as an additive and coating), and food additives, to identify and prioritize potential occupational, health, consumer, and environmental risks. Exposure scenarios were developed and ranked for each product life-cycle stage. A Safer-by-Design Toolbox (SbD Toolbox) representing a compendium of high-throughput physical, chemical, and toxicological new approach methodologies (NAMs) was used for a screening-level hazard assessment. Overall, risks identified for the CN-enabled products were low. Of the exposure scenarios, occupational inhalation exposures during product manufacturing and application ranked the highest. Despite differences in chemistry and morphology, the materials behaved similarly in oral, dermal, and inhalation models, supporting their grouping and read-across. The screening-level hazard assessment identified potential lung inflammation associated with CN exposure, and a review of the literature supported this funding, suggesting CNs behave as poorly soluble, low-toxicity dusts with the potential to irritate the lung. Key research gaps to reduce uncertainty include evaluating long-term, low-dose exposures typical of the workplace, as well as the potential release and toxicity of CN-containing composite particles.

## 1. Introduction

Cellulose nanomaterials (CNs) are nanoscale forms of cellulose, the most abundant natural polymer on earth. CNs are used in numerous applications, including composites, paints and coatings, cosmetics, food and food packaging, and medical and dental devices [1]. Primarily derived from wood, CNs offer sustainable, high-performance alternatives to traditional petrochemicals [2].

First-generation cellulose materials, developed from cellulose feedstocks (e.g., cellulose pulp), are highly refined forms of cellulose fibers [3]. They are typically referred to as microfibrillated cellulose (MFC), fibrillated cellulose, or unmodified cellulose nanofibers or nanofibrils (CNF). MFC is typically manufactured by mechanically freeing cellulose fibrils from bleached or unbleached wood pulp. Sharing a molecular structure with conventional cellulose, MFC is a heterogenous material consisting of cellulose fibers, fines, and microfibrils in various proportions [4]. As with refined wood pulp, the microfibrils are generally attached to the pulp fibers or exist in an entangled network of fibers. MFC has a hierarchical branching structure where the fibers (mm- to µm-scale diameters) branch out into smaller fibrils (nm-scale diameters) toward their ends. Those finer micro and elementary fibrils have high aspect ratios (i.e., several hundred microns in length). This morphology imparts MFC with several unique properties for commercial applications, including high strength; lightweighting; absorbency; viscosity modification; optical transparency; and sound, grease, oil, and oxygen barrier [3,5,6].

The safety of first-generation cellulose materials has been extensively evaluated in conventional animal models [7,8,9,10,11] and non-animal new approach methodologies (NAMs) [12]. Studies have shown that MFC is well-tolerated following oral and dermal exposures and acts similarly to poorly soluble low-toxicity (PSLT) dusts when inhaled. These data support that MFC is a safer and more sustainable alternative to many traditional chemicals in a variety of applications.

Despite their demonstrated safety and sustainability, the performance of first-generation cellulose materials can be advanced by imparting new surface chemistries that either improve or enable new commercial applications. For example, in food packaging applications, surface hydroxyl groups impart high hydrophilicity to cellulose, resulting in swelling in humid conditions and in the presence of moist foods. This swelling can reduce barrier properties and performance [13]. To address these issues, chemical modifications of cellulose materials are critical to improve their performance (e.g., as barrier coatings) and to allow for their use in composite materials (e.g., cement, packaging, paints and coatings, polymers, and cosmetics) [14].

Chemically-modified CNs represent the second generation of cellulose materials. Developed from cellulose feedstocks, they offer improved barrier properties, biocompatibility, selective absorbance, and antioxidant and antimicrobial activity, extending their commercial applications [15,16,17,18]. However, new physical and chemical properties, including new surface chemistries, may impact the safety of a substance [15,19,20] and its regulatory status. Since second-generation CNs represent an emerging class of nanomaterials (NMs), safety evaluations are necessary to responsibly commercialize these materials, support regulatory acceptance, and realize their potential as advanced, sustainable materials.

To promote the safe and responsible commercialization of second-generation CNs, there is a need to evaluate their safety in commercially-relevant forms. Toward this end, we identified second-generation CNs with high technological-readiness and numerous commercial applications (i.e., sulfated and carboxylated surface chemistries). We synthesized, characterized, and evaluated the safety of these commercially-relevant CNs with various charge levels and morphologies. We also developed high-throughput toxicological assays to assess the potential CN hazards associated with three exposure routes:

- Oral: a simulated digestion and intestinal tri-culture model was used to replicate the biological complexity of the human gut, including enterocytes, goblet cells, and immune cells;
- Inhalation: a co-culture of human lung epithelial cells and dendritic (immune) cells was used to replicate the epithelium of the human lung;
- Dermal: a co-culture of dermal epithelial cells and dermal fibroblasts was used to replicate the human epidermis.

Funded by P3Nano, this project, “Safer-by-Design Toolbox to Advance Safer Manufacturing of Functionalized CNs”, developed NAMs for CNs, and compiled physicochemical and safety data and methods in a publicly-available Safer-by-Design Toolbox (SbD Toolbox). The SbD Toolbox is intended to aid in:

- Pre-commercial safety screening of new chemistries early in the research and development process;
- Reducing the time and cost for conducting pre-commercial safety evaluations;
- Generating comprehensive physical, chemical, and toxicological data using NAMs to determine if CNs can be grouped together for the purpose of read-across, following established guidance for NMs [21];

If testing supports grouping CNs for read-across, hazards for a group of CNs (e.g., oral toxicity, dermal toxicity) may be assessed from one representative material within said group, using supporting toxicity data from the peer-reviewed literature. This would enable resource-efficient safety screening to promote the responsible commercialization of second-generation CNs.

While the data and methods detailed in the SbD Toolbox may be used to assess the safety of CNs, a holistic consideration of the materials is required to determine their risks in realistic use cases. Nanomaterial life-cycle risk assessment (Nano LCRA) is a proactive approach to NM risk assessment and management that considers safety across the entire life-cycle of a product [7]. The adaptive, screening-level risk assessment framework involves describing the product life-cycle, developing qualitative exposure scenarios, ranking exposure scenarios using exposure criteria, evaluating potential hazards and exposures, and a final qualitative evaluation of risks for the intended application. Previous Nano LCRAs have been successful in furthering the safe and sustainable development of advanced materials in emerging technologies [7,22,23].

Nano LCRA is practical for pre-manufacturing evaluations, as it adopts worst-case assumptions, identifying potential risks in advance of actual exposure, and allowing changes in manufacturing or risk management early in the product development process. This SbD approach incorporates conservatism into product design and risk management decisions under uncertainty, while providing key insights for future data needs to inform more rigorous and quantitative assessments.

We conducted a Nano LCRA to identify and prioritize the human and environmental health risks of functionalized CNs. As risk depends on both the nature of the hazard as well as the level of exposure, data for both were developed for the Nano LCRA. We first identified potential exposure scenarios for occupational, consumer, and environmental receptors in five case studies (CSs), including the use of functionalized CNs in food contact (e.g., water filtration, food packaging) and food additive applications. Exposure scenarios were ranked and prioritized by applying exposure criteria to identify priority exposure pathways. Exposure assessments were then refined by evaluating available studies in the peer-reviewed literature characterizing potential occupational, consumer, or environmental CN exposures.

For high-ranking exposure scenarios, we evaluated potential hazards using data in the literature and the SbD Toolbox and assessed whether second-generation CNs can be grouped together for read-across as part of their hazard assessment.

These two analyses, exposure and hazard assessment, were then brought together to qualitatively evaluate occupational, health, and environmental risks from CNs for the five evaluated case studies. The findings from this analysis elucidate: (i) potential risks from the use of second-generation CNs in food and food packaging; (ii) safer CN forms or surface chemistries for these applications; and (iii) the utility of the SbD Toolbox to inform potential hazards and evaluate risks. The Nano LCRA also prioritizes outstanding data gaps that, when filled, will allow for a more comprehensive safety evaluation for the responsible commercialization of functionalized CNs in food and food packaging.

## 2. Materials and Methods

### 2.1. SbD Toolbox CN Synthesis and Characterization

Microfibrillated cellulose (MFC, University of Maine Process Development Center, Orono, ME, USA) was purchased and used as an unmodified reference material (i.e., first-generation CN) alongside industrially relevant surface-functionalized CNs (i.e., second-generation CNs) [24]. Sulfated (acid hydrolyzed) and carboxylated (2,2,6,6-tetramethylpiperidin-1-oxyl radical [TEMPO]-oxidized or sequential periodate-chlorite [PC]-oxidized) CNFs (SCNF, TCNF, and PCCNF, respectively) were strategically designed, synthesized, and characterized (physically, chemically, and toxicologically) with the SbD Toolbox’s methodologies. TCNF, PCCNF, and SCNF were prepared by varying the (i) the reagent concentration and reaction time, and (ii) the high-speed blending time (Table 1) [12,24]. For each chemistry (SCNF, TCNF, and PCCNF), four materials (SCNFa-d, TCNFa-d, and PCCNFa-d) were manufactured and designed to span different charge levels and morphologies. The materials were characterized with a combination of atomic force microscopy (AFM, Asylum Research MFP-3D, Oxford Instruments, Concord, MA, USA) to evaluate overall morphology, including CNF lengths and thicknesses, and conductometric titration to differentiate surface charges between materials [24]. The synthesis and characterization methodologies are available in the SbD Toolbox Section 3.1 PChem Database, and have been previously published [25,26]. Representative atomic force micrographs for each material are in the SbD Toolbox Section 3.1 PChem Database and in Supplementary Materials, Figure S1.

### 2.2. SbD Toolbox Hazard Assessment

#### 2.2.1. Human Intestinal System Model

Each CN underwent standardized sample dispersion and simulated gastrointestinal digestion, as described in the SbD Toolbox Section 2.2 [12,24]. Before and after digestion, diluted CN dispersions were characterized. Digested SCNF, TCNF, and PCCNF dispersions were prepared at concentrations ranging from 0.2–2% *w*/*v* in complete cell culture media (complete Dulbecco’s Modified Eagle Medium/F-12, Gibco, Thermo Fisher Scientific Inc., Waltham, MA, USA) via serial dilutions for subsequent toxicological testing. Digested CNs were exposed to a co-culture model composed of human Burkitt’s lymphoma Raji B cells, human colon carcinoma Caco-2 cells, and HT29-MTX cells from the American Type Culture Collection (ATCC, Manassas, VA, USA) in a 9:9:1 ratio [24,27].

#### 2.2.2. Human Inhalation System Model

Human epithelial lung cells (A549) and dendritic cells (JAWSII) from ATCC (Manassas, VA, USA) were seeded in 1:1 ratio, as described in the SbD Toolbox Section 2.3. A549 epithelial cells from ATCC (Manassas, VA, USA) were grown in complete RPMI (cRPMI) 1640 (Thermo Fisher Scientific Inc., Waltham, MA, USA) supplemented with 10% Fetal Bovine Serum (FBS, Corning, Tewksbury, MA, USA) and 1% penicillin-streptomycin (Gibco, Billings, MO, USA). JAWSII cells from ATCC (Manassas, VA, USA) were cultured in complete Alpha minimum essential medium (cAMEM) with nucleosides (Thermo Fisher Scientific Inc., Waltham, MA, USA) and supplemented with 5 ng/mL murine GM-CSF (BioLegend, San Diego, CA, USA); 20% FBS; and 1% penicillin-streptomycin. Cells were maintained at 37 °C in a humidified 5% CO_2_ atmosphere. A549 cells were added to well plates fitted with polyethylene terephthalate (PET) Transwell^®^ membranes (Corning, NY, USA) at 28 × 104 cells/cm^2^. Cells adhered and after 2–3 days a confluent monolayer was formed. The media was removed, and the inserts were inverted and placed into sterile glass petri dishes. JAWSII cells were resuspended in 500 μL of complete media and plated on the basal surface of the membrane at 7 × 104 cells/cm^2^ and allowed to adhere for 4 h. After adherence, excess media was removed, inserts were reverted into the well plate, and 1 mL of cAMEM was added to the basolateral chamber. The in vitro alveolar cell culture model was completed after replenishing media to a total volume of 500 μL. The model was then placed in a 37 °C humidified incubator (Labconco, Kansas City, MO, USA) at 5% CO_2_ atmosphere for 24 h before CN exposure.

CN exposures were conducted with the Vilnius aerosol generator (VAG) obtained from CH Technologies (Westwood, NJ, USA). The VAG and a compressed air supply were used to produce aerosols of each cellulose type with total mass concentration verified by a Casella real-time dust monitor (Casella Solutions, Rutland, VT, USA).

#### 2.2.3. Human Dermal System Model

Human dermal fibroblasts (HDFa) and human epidermal keratinocytes (HEKa) from ATCC (Manassas, VA, USA) were seeded in 1:1 ratio, as described in the SbD Toolbox Section 2.4. Briefly, cryopreserved primary HDFa cells (PCS-201-010, ATCC) were cultured in Dulbecco’s Modified Eagle’s Medium (Gibco, Billings, MO, USA) supplemented with 10% FBS (Corning, Tewksbury, MA, USA). HEKa cells (CRL-2404, ATCC) were cultured in serum-free keratinocyte medium (Thermo Fisher Scientific Inc., Waltham, MA, USA) supplemented with 25 g/mL bovine pituitary extract and 0.15 ng/mL recombinant epidermal growth factor (Thermo Fisher Scientific Inc., Waltham, MA, USA). Media was supplemented with an antibiotic cocktail of penicillin, streptomycin, and amphotericin (Sigma–Aldrich, St. Louis, MO, USA). Incubation (Labconco, Kansas City, MO, USA) took place at 37 °C with humidity and 5% CO_2_. Cells were grown to 80% confluency in well plates, then exposed to CNs or untreated for negative control. Each experiment was performed in triplicate.

#### 2.2.4. Cytotoxicity via Membrane Disruption

The concentration of lactate dehydrogenase (LDH) in the co-culture supernatants after cellulose exposure was quantified using CyQUANT LDH Cytotoxicity Assay kit (Invitrogen C20301, Thermo Fisher Scientific Inc., Waltham, MA, USA), as described in the SbD Toolbox Sections 2.3 and 2.4. A 1× LDH positive (reaction) control was run parallel to Triton X-100 positive control (Amresco, Solon, OH, USA). Sterile ultrapure water was used to quantify spontaneous LDH activity, and 10× lysis buffer was added to quantify the maximum LDH activity per kit recommendations. After post-exposure periods (15 min or 4 h), supernatants were transferred to a 96-well flat-bottom plate (Thermo Fisher Scientific Inc., Waltham, MA, USA) in triplicate. The reaction mixture and stop solution were added to wells according to the assay kit, and absorbance was measured at 490 and 680 nm on a Biotek Epoch2 microplate reader (Agilent Technologies, Santa Clara, CA, USA). Percent (%) cytotoxicity was calculated.

#### 2.2.5. Cellular Pro-Inflammatory Response

Pro-inflammatory response markers interleukin-6 (IL-6, for lung and intestinal) and interleukin-1b (IL-1b, for dermal) were quantified in pg/mL in CN-exposed cell lysates using an Enzyme-Linked Immunosorbent Assay (ELISA, Invitrogen, Thermo Fisher Scientific Inc., Waltham, MA, USA), as described in the SbD Toolbox Sections 2.3 and 2.4. Briefly, CN samples were added to the cell well plates for 15 min or 4 h at humidified 37 °C and 5% CO_2_ atmosphere (Labconco, Kansas City, MO, USA), after which supernatant was removed. Cells were washed 2X with cold sterile 1X PBS (Gibco, Billings, MO, USA). The cell lysate was collected in 1 mL microcentrifuge tubes and centrifuged for 10 min at 12,000× RPM at 15 °C (Eppendorf 5810, Thermo Fisher Scientific Inc., Waltham, MA, USA). The pellet was discarded, and the supernatant was stored at −80 °C until use. Absorbance was read at excitation and emission wavelengths (BioTek Synergy, Agilent, Santa Clara, CA, USA) per the manufacturer’s instruction. Data were analyzed utilizing a four-parameter curve. In measuring both cytotoxicity and pro-inflammatory response, simulated fluid (e.g., gastric fluid) was used as the vehicle control, and 1% (*v*/*v*) Triton X-100 (Thermo Fisher Scientific Inc., Waltham, MA, USA) was used as a positive control.

#### 2.2.6. Statistical Analysis

One-way Analysis of Variance (ANOVA) and independent *t*-tests (*p* < 0.05) were performed to determine statistically adverse effects compared to the vehicle control groups and derive either a No Observed Adverse Effect Level (NOAEL) if no adverse effects were found, or a Lowest Observed Adverse Effect Level (LOAEL) if adverse effects from CN exposure were identified. The NOAEL and LOAEL benchmark values were used to assess the potential hazards for these materials and whether they can be grouped together for read-across.

#### 2.2.7. SbD Toolbox for Read-Across

The generated physical, chemical, and safety data were compiled into a SbD Toolbox for ease of comparison. Using these data, we evaluated whether second-generation CNs can be grouped with first-generation cellulose materials to read-across the large body of safety data demonstrating safe use in food and food packaging applications. If a particular chemistry or morphology behaved differently (i.e., posed a greater hazard) than other CNs, it was flagged for further safety evaluations.

### 2.3. LCRA Overview

Nano LCRA is an adaptive, screening-level risk assessment framework. The iterative process evaluates risks across the product life-cycle to inform early decision-making about nanoscale technologies and provide analysis to support SbD strategies. The initial Nano LCRA steps include describing the life-cycle, identifying potential hazards, and conducting an exposure assessment at each life-cycle stage.

The analysis identifies qualitative exposure scenarios impacting workers, the public, and the environment across the product life-cycle. These scenarios are ranked using exposure criteria to identify priority pathways, which are evaluated via exposure and hazard assessment to assess potential risks.

**Figure 1:**
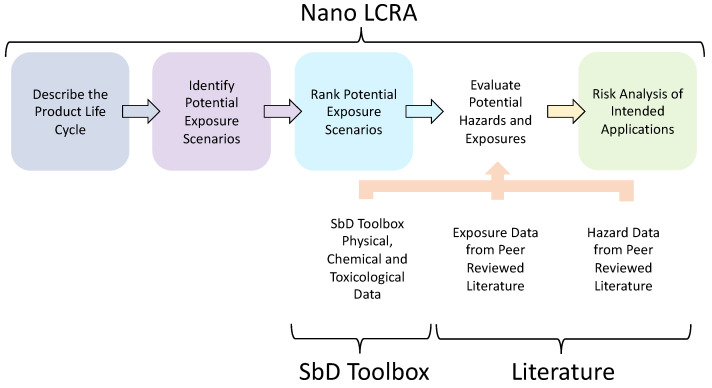
Overview of Nano LCRA and role of SbD Toolbox in hazard and exposure assessment.

The methods and data in the SbD Toolbox inform the hazard and exposure evaluation conducted as part of the Nano LCRA (Figure 1). The SbD Toolbox includes new approach (i.e., non-animal) methods, models, and data to screen for potential oral, dermal, and inhalation hazards. The side-by-side testing designed as part of the SbD Toolbox also allows evaluation of whether CNs can be grouped together for the purpose of read-across. If CN groupings can be justified, read-across can be applied to predict endpoint specific information (e.g., oral toxicity, dermal toxicity) from similar forms of CNs in the SbD Toolbox and the peer-reviewed literature.

#### 2.3.1. Case Study Development

CN chemistries and applications with high commercial potential were identified by surveying CN stakeholders (i.e., government agencies, research and technology organizations, companies, and researchers) and conducting a cursory literature review. Three CN chemistries were selected for evaluation: TCNFs, PCCNFs, and SCNFs (Table 2) [25,26]. These materials were evaluated side-by-side with first-generation cellulose materials (i.e., MFC).

The three applications adopted as CSs for the Nano LCRA are: (i) water filtration (CS1); (ii) food contact packaging (i.e., food packaging film [CS2.1], food packaging coating [CS2.2], and food packaging additive [CS2.3]); and (iii) food additive (CS3).

#### 2.3.2. Life-Cycle Stages and Assumptions

The product life-cycle for each CN CS is mapped in six stages, starting with raw material processing (i.e., wood pulp) for CN manufacture and ending with disposal of CN-containing products at the product end-of-life.

1. *Raw Material*. This stage includes harvesting, chipping/shredding, and pulping of hardwood or softwood to produce bleached or unbleached cellulose pulp. The cellulose pulp is used as a raw material for CN production in later life-cycle stages.
2. *Product Manufacturing*. The raw material is treated and processed to manufacture carboxylated or sulfated CNs. While production and chemical inputs differ by CN chemistry, all are produced in enclosed batch reactors with large-scale production capacity. The CNs are manufactured as low weight percentage (<2 wt %) aqueous dispersions. Production includes a drying step to produce CN powders (up to 100 wt %). The CNs are packaged, sold, and transported as powder.
3. *Product Application*. The CNs are manufactured into commercial products with different intended applications. The handling, manufacture, and intended use of these products vary between each of the five selected CSs. Generally, CN materials are transported as a powder into a manufacturing facility. The CN powders are handled and may be redispersed into an aqueous suspension as part of product application. For CS1 (i.e., CN water filtration membrane) and CS2.1 (i.e., CN food packaging film), a CN formulation is prepared, and a casting and drying process is used to manufacture water filter membranes (up to 10 wt % CN; CS1) and food packaging films (up to 100 wt % CN; CS2.1). In CS2.2 (i.e., CN-coated food packaging), the CN formulation (2 wt % CN) is coated on paper/board food contact packaging using spray coating. The coating is dried/cured and may contain up to 100% CN. In CS2.3 (i.e., CN food packaging additive), the CN dispersion is mixed with traditional ingredients used to manufacture food contact paper/board. The CS assumes traditional paper/board production methods (e.g., screening, pressing, drying). In CS3 (i.e., CN food additive), food manufacturers may add CNs up to 5 wt % into food using high shear mixing, or similar processes in an open system. CNs are incorporated into food for a variety of technical effects including use as a stabilizer, emulsifier, thickener, or caloric reduction. Further details of the product application life-cycle stage for each CS are outlined in Table 3.
4. *Product Use*. The five CSs involve CN-containing products for use in water filtration (CS1), food contact materials (CS2.1–2.3), and foods (CS3) and are detailed in Table 3. For CS1 (i.e., CN water filtration membrane), it is assumed the membrane is handled during instillation, and that is it primarily used for water filtration intended for consumption. The CN water filtration membrane may be used for large-scale filtration or for direct use by consumers. For CS2.1–2.3 (i.e., food contact applications), CNs are used as a single-use food packaging film, or as a coating or additive in food packaging paper/board. Under CS3, CNs are consumed as an additive in a variety of foods.
5. *Re-use/Recycling*. After a typical service life which varies by CS (e.g., food contact applications in CS2.1–2.3 are single-use products), the CN products are re-used, recycled, or composted, or they may remain in use beyond their recommended lifespan. Recycling activities may include physical (e.g., shredding, tearing) or chemical processes (e.g., pulping). Re-purposing activities may include bioconversion processes. Alternatively, CN products may be composted (particularly for applications in food and food contact materials) at either consumer or industrial scale.
6. *Disposal*. The original or reprocessed CN products are disposed of as waste. Consumer products typically end up in a landfill or incinerated for heat recovery. The products may also be discarded intentionally or unintentionally in an uncontrolled environment.

Case study development required the adoption of several assumptions; these are summarized for all five CSs in Table 3 with further details in Supplementary Materials, Tables S1–S5. Where possible, assumptions are based on the materials and manufacturing methods for each application detailed in the peer-reviewed literature. Importantly, the Nano LCRA is focused on the safety evaluation of the CN or CN composite materials and does not consider potential risks from other materials or ingredients.

#### 2.3.3. Exposure Scenario Development

For each life-cycle stage, possible scenarios that could lead to occupational, public, or environmental exposure were identified for each CS. Each scenario was then evaluated by incorporating potential exposure criteria, resulting in a mini conceptual exposure model connecting the use of CN and CN-enabled consumer products to potential receptors. For each CS, the process was repeated for each life-cycle stage until the product’s end-of-life. The different combinations of application, receptor, hazard, and exposure (both intentional and accidental), assessed across the life-cycle, led to the development of discrete exposure scenarios.

#### 2.3.4. Exposure Scenario Ranking

Following scenario development, the developed exposure scenarios for each CS were ranked by applying exposure criteria to identify priority exposure pathways. Exposure estimates were then developed for the highest priority pathways. Four dimensions of exposure were used to rank the exposure scenarios in each CS, following the methodology outlined in the Nano LCRA framework [7]:

1. Directness of exposure, which relates to the potential for direct contact of CNs (i.e., how easily particles are released);
2. Magnitude, which relates to the relative degree of exposure based on the percentage of CN in the product;
3. Likelihood, which prioritizes intentional exposures over unintentional or accidental ones;
4. Frequency, an estimate of how often an exposure is expected.

For each scenario, a relative score of low (1), medium (2), or high (3) was assigned for each of the four criteria. The criteria used are outlined in Table 4. Importantly, the analysis adopts a conservative assumption that no engineering controls are employed, and no PPE is worn. Summing the scores characterizes the potential CN exposure scenarios. The highest scores indicate the top-ranked scenarios with the most significant exposure potential.

#### 2.3.5. Exposure Assessment

We evaluated available information and data in the peer-reviewed literature on potential exposures to second-generation CNs (TCNF, PCCNF, and SCNF) in the workplace, to the public, and in the environment to better characterize exposure and risk across the product life-cycle. In cases where limited data are available, studies evaluating first-generation cellulose materials (e.g., MFC) are discussed. This section also evaluates the literature examining potential release and exposure to CNs from composite materials, such as food packaging. Where no data on relevant CN composites in the CSs (e.g., food packaging materials) are available, studies evaluating CN releases from other relevant matrix materials are described.

#### 2.3.6. Hazard Assessment

A screening-level oral, dermal, and inhalation hazard assessment of TCNF, PCCNF, and SCNF was first conducted using the hazard screening tools in the SbD Toolbox.

A literature review was used to complement the SbD Toolbox hazard assessment and identify potential hazards associated with TCNF, PCCNF, or SCNF exposure across the product life-cycle. Studies evaluating human health (in vivo and in vitro) and ecotoxicity were identified for carboxylated and sulfated CNs. Data gaps were identified by evaluating the current state of the hazard literature against potential hazards across the life-cycle for the five CSs.

#### 2.3.7. Qualitative Health and Environmental Risk Characterization

Using available information on exposure (Section 3.6) and hazard (Section 3.7 and Section 3.8), the potential risks associated with the top-ranking exposure scenarios were qualitatively characterized (Section 4).

## 3. Results

### 3.1. Exposure Scenario Development and Ranking

The potential exposure scenarios identified for the use of CNs in a water filtration membrane (CS1) are outlined in Table 5 as an example. Exposure scenarios developed for each CS (CS1, CS2.1, CS2.2, CS2.3, and CS3) are provided in Supplementary Materials, Tables S6–S10. Each table displays the life-cycle stage, scenario, receptor, and exposure route associated with each scenario in each CS.

**Figure 2:**
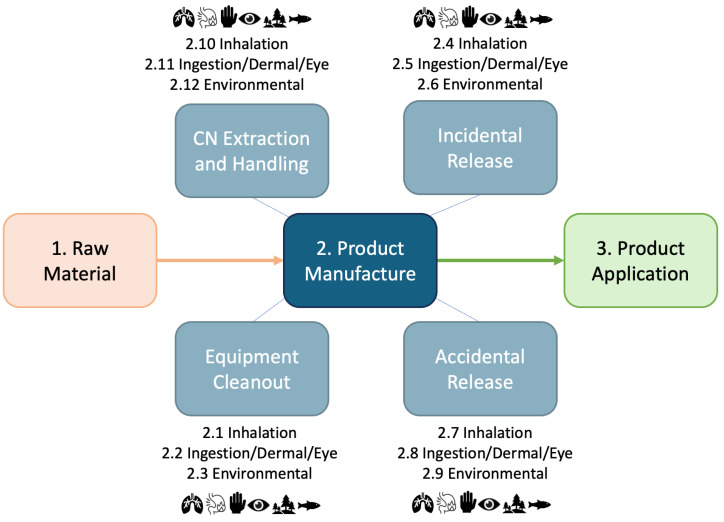
Exposure scenarios for the product manufacture life-cycle stage (Scenarios 2.1–2.12).

Figure 2 shows an example of the exposure scenarios developed for life-cycle Stage 2 (product manufacture), which is similar across the five CSs examined. Here, functionalized CNs (either TCNF, PCCNF, or SCNF) are manufactured by treating and processing cellulose pulp, which is subsequently dried to produce a CN powder. A worker transferring the CN from the reactor/drier to containers for commercial sale could inhale CN powder (Scenario 2.10) or could be exposed by touching their skin or eyes (Scenario 2.11). For all scenarios where dermal exposure is possible, it is also assumed ingestion of CN via hand-to-mouth transfer is possible. Scenarios 2.1, 2.2, and 2.3 describe potential inhalation; dermal or eye; or environmental exposure from CN reactor and drier cleanout. Incidental release of CN during manufacture, either as a liquid aerosol (during production) or powder (during drying) could potentially lead to inhalation (Scenario 2.4), dermal/eye contact (Scenario 2.5), or environmental release (Scenario 2.6). Inhalation (Scenario 2.7), eye/dermal contact (Scenario 2.8), or environmental release (Scenario 2.9) could occur during an accidental release or spill associated with CN manufacture.

Stage 3 is product application. In this stage, the CNs are manufactured into commercial products with different intended applications. The exposure scenarios differ based on the handling, manufacture, and intended use of the products in each of the five CSs. However, several exposure scenarios at this life-cycle stage are common to each CS. Occupational inhalation or dermal/eye exposure to dry CNs may occur during transfer and handling, as could an environmental exposure if there is release to the environment. As in Stage 2, equipment cleanout may result in inhalation, dermal/eye, or environmental exposure. In most CSs, it is assumed CNs will be redispersed as part of product application, with the potential for inhalation or dermal/eye exposures. Across all CSs, the product application stage also has potential inhalation, dermal, or environmental exposures associated with incidental release of CNs from production equipment or accidental spills.

The remaining exposure scenarios in the product application stage are unique to each evaluated CS. Production of a CN water filtration membrane (CS1) and a CN food packaging film (CS2.1) involve potential inhalation and dermal/eye exposures to workers associated with release of CNs during formulation preparation (e.g., mixing with other ingredients), casting the CNs into a membrane or film, drying the membrane or film, and any final treatments to the membrane or film (Supplementary Materials, Tables S6 and S7). In CS2.2 and CS2.3 (the production of a CN coating or food contact paper/board containing CNs), inhalation or dermal/eye exposures to workers are possible during spray coating of CNs on paper/board, or as the CN-containing paper is manufactured (e.g., screening, pressing). Potential inhalation and dermal/eye exposures were also associated with CN release during drying, coating, or packaging treatments, and physical manipulation of the coating or packaging (e.g., bending, folding; Supplementary Materials, Tables S8 and S9). When used as a food additive (CS3), CN inhalation and dermal/eye exposures to workers may occur during the mixing/processing of CNs into food, or through release from food during packaging and handling (Supplementary Materials, Table S10).

Stage 4 is the product use stage, where the CN-containing products are used or consumed by end-users. For each CS, the exposure scenarios are dependent on the intended use of the CN-enabled product and are summarized in Supplementary Materials (Tables S6–S10). Use of a CN membrane for water filtration (CS1) may result in dermal/eye exposures to workers or consumers, or potential inhalation exposures if CNs are released from the membrane during handling, instillation, or removal. Oral exposures are also possible to workers and consumers if CNs migrate from the membrane to drinking water during use. Environmental exposures may occur if filtered water is released to the environment. Where CNs are used in food contact materials (as a film, coating, or additive; CS2.1–2.3), consumers may be exposed to CNs if migration to food occurs, or if CNs are released from packaging during handling or misuse. Manufactured foods containing CNs (CS3) will result in oral exposures to consumers, and potentially dermal exposures associated with the handling of food.

In Stage 5 (re-use/recycling), the CN-enabled products in all five CSs may be used beyond the recommended product life, which will impact the degree to which CNs may be released. Inhalation or dermal/eye exposure to workers associated with transportation, recycling, re-use, or composting activities may occur in this stage. Environmental exposures are also possible if CNs are released to the environment during activities such as composting.

During life-cycle Stage 6 (disposal), the CN-containing products are disposed of; potential exposure scenarios are similar across the five CSs. Release of CN from these products is possible during transfer to disposal facilities or end-of-life treatments, such as incineration. Occupational exposures associated with these activities are primarily via inhalation or dermal pathways. Environmental release and exposure are also possible from disposal activities (e.g., transport; incineration; landfilling) and discarding in an uncontrolled environment (e.g., littering).

### 3.2. Exposure Scenario Ranking Results

Following exposure scenario development, the exposure scenarios were ranked by applying exposure criteria (directness of exposure, magnitude, likelihood, and frequency) to identify priority exposure pathways (see Section 2.3.4 for methodological details). Summing the scores characterizes the potential CN exposure scenarios. The highest scores indicate the top-ranked scenarios with the most significant exposure potential. Table 5 shows the results of the exposure scenario scoring and ranking for CS1.

Due to similarities in CN production, handling, and product manufacture, many of the developed exposure scenarios were similar across the five CSs, especially in early life-cycle stages such as product manufacturing and product application. The results from exposure scenario scoring and ranking for each CS can be found in Supplementary Materials, Tables S11–S14.

### 3.3. High Ranking Exposure Scenarios Common to All Five CSs

The highest-ranking exposure scenarios common to all five CSs are summarized in Table 6. The table details each top-ranking exposure scenario, results from the exposure ranking exercise, and the specific CSs that share a given exposure scenario.

The top-ranking scenarios (2.10, 2.11, 3.1, 3.2, 3.6, and 3.7; Table 6) all involve the potential for direct occupational exposure during product manufacturing and application life-cycle stages. Predominant exposure routes are via inhalation or dermal/eye contact (and incidental ingestion from hand-to-mouth transfer) associated with handling CN powder during manufacture and its rehydration/dispersion during product application. These activities rank high on all four exposure criteria and were the top-ranked exposure scenarios for all five CSs.

The next highest-ranking scenarios (3.19, 3.21) common to most CSs involve final product processing steps (e.g., drying steps, surface treatments) in the product application stage, which ranked high for magnitude, likelihood, and frequency of potential occupational dermal exposures. Dermal occupational exposures (5.2) also ranked highly across all CSs during re-use and recycling activities (life-cycle Stage 5) of CN-containing products. Highly-ranked potential environmental exposure scenarios (5.7, 6.6) were identified during re-use/recycling and disposal life-cycle stages, and involved activities associated with composting or landfilling CN-containing products (Table 6).

**Table 5:** Exposure scenario ranking for CS1: water filtration membrane [TCNF, PCCNF (carboxyl functionalization)].

Life-Cycle Stage	LCSC	LCSN	SN	Scenario	Receptor	ER	DE	M	L	F	Score	Rank
Product Manufacturing	PM	2	10	Dried formulation extraction and handling (for powder NC ingredients)	occupational	inhalation	3	3	3	3	12	1
Product Manufacturing	PM	2	11	Dried formulation extraction and handling (for powder NC ingredients)	occupational	ingestion/dermal/eye	3	3	3	3	12	1
Product Application	PA	3	1	Transfer CN from synthesis to application facility (e.g., handling, packaging)	occupational	inhalation	3	3	3	3	12	1
Product Application	PA	3	2	Transfer CN from synthesis to application facility (e.g., handling, packaging)	occupational	ingestion/dermal/eye	3	3	3	3	12	1
Product Application	PA	3	6	CN rehydration (if powder NC ingredient)	occupational	inhalation	3	3	3	3	12	1
Product Application	PA	3	7	CN rehydration (if powder NC ingredient)	occupational	ingestion/dermal/eye	3	3	3	3	12	1
Product Application	PA	3	19	CN drying (membrane formation)	occupational	ingestion/dermal/eye	2	3	3	3	11	2
Product Application	PA	3	21	Surface treatment of CN membrane (functionalization, other)	occupational	ingestion/dermal/eye	2	3	3	3	11	2
Re-use/Recycling	RR	5	2	Collection and transport of used membrane to re-use or composting facility	occupational	ingestion/dermal/eye	2	3	3	3	11	2
Re-use/Recycling	RR	5	7	Composting used membrane	environmental	direct	2	3	3	3	11	2
Re-use/Recycling	RR	5	11	Continued use (degraded membrane)	consumer	ingestion/dermal/eye	2	3	3	3	11	2
Re-use/Recycling	RR	5	12	Continued use (degraded membrane)	environmental	direct	2	3	3	3	11	2
Disposal	D	6	5	Long-term MSW landfill storage	environmental	direct	2	3	3	3	11	2
Product Manufacturing	PM	2	1	Cleaning out synthesis equipment	occupational	inhalation	2	2	3	3	10	3
Product Manufacturing	PM	2	2	Cleaning out synthesis equipment	occupational	ingestion/dermal/eye	2	2	3	3	10	3
Product Application	PA	3	3	Deposition and formulation equipment cleanout	occupational	inhalation	2	2	3	3	10	3
Product Application	PA	3	4	Deposition and formulation equipment cleanout	occupational	ingestion/dermal/eye	2	2	3	3	10	3
Product Application	PA	3	8	CN formulation preparation (e.g., mixing with other ingredients, homogenizing, other)	occupational	inhalation	2	2	3	3	10	3
Product Application	PA	3	9	CN formulation preparation (e.g., mixing with other ingredients, homogenizing, other)	occupational	ingestion/dermal/eye	2	2	3	3	10	3
Product Application	PA	3	10	CN casting to form membrane	occupational	inhalation	2	2	3	3	10	3
Product Application	PA	3	11	CN casting to form membrane	occupational	ingestion/dermal/eye	2	2	3	3	10	3
Product Use	PU	4	4	Use of membrane to filter drinking water	occupational	ingestion	2	2	3	3	10	3
Product Use	PU	4	5	Use of membrane to filter drinking water	consumer	ingestion	2	2	3	3	10	3
Product Use	PU	4	6	Use of membrane to filter drinking water	environmental	direct	2	2	3	3	10	3
Re-use/Recycling	RR	5	4	Composting used membrane	occupational	ingestion/dermal/eye	2	3	2	3	10	3
Product Application	PA	3	18	CN drying (membrane formation)	occupational	inhalation	2	3	1	3	9	4
Product Application	PA	3	20	Surface treatment of CN membrane (functionalization, other)	occupational	inhalation	2	3	1	3	9	4
Product Use	PU	4	2	Membrane handling, installation, and removal	occupational	ingestion/dermal/eye	2	3	3	1	9	4
Product Use	PU	4	3	Membrane handling, installation, and removal	consumer	ingestion/dermal/eye	2	3	3	1	9	4
Re-use/Recycling	RR	5	1	Collection and transport of used membrane to re-use or composting facility	occupational	inhalation	2	3	1	3	9	4
Product Manufacturing	PM	2	4	Incidental release of CN from synthesis equipment	occupational	inhalation	2	2	2	2	8	5
Product Manufacturing	PM	2	5	Incidental release of CN from synthesis equipment	occupational	ingestion/dermal/eye	2	2	2	2	8	5
Product Manufacturing	PM	2	6	Incidental release of CN from synthesis equipment	environmental	direct	2	2	2	2	8	5
Product Application	PA	3	12	Incidental release of CN from deposition equipment	occupational	inhalation	2	2	2	2	8	5
Product Application	PA	3	13	Incidental release of CN from deposition equipment	occupational	ingestion/dermal/eye	2	2	2	2	8	5
Re-use/Recycling	RR	5	6	Composting used membrane	consumer	ingestion/dermal/eye	2	3	2	1	8	5
Re-use/Recycling	RR	5	9	Bioconversion (anaerobic digestion)	occupational	ingestion/dermal/eye	2	3	2	1	8	5
Re-use/Recycling	RR	5	10	Bioconversion (anaerobic digestion)	environmental	direct	2	3	2	1	8	5
Disposal	D	6	1	Collection and transport to final end-of-life location	occupational	ingestion/dermal/eye	2	3	2	1	8	5
Disposal	D	6	2	Collection and transport to final end-of-life location	environmental	direct	2	3	2	1	8	5
Disposal	D	6	3	Incineration or heat recovery	occupational	inhalation	2	3	2	1	8	5
Disposal	D	6	4	Incineration or heat recovery	environmental	direct	2	3	2	1	8	5
Disposal	D	6	6	Membrane discarded in uncontrolled environment	environmental	direct	2	3	2	1	8	5
Product Manufacturing	PM	2	3	Cleaning out synthesis equipment	environmental	direct	2	2	2	1	7	6
Product Manufacturing	PM	2	7	Accidental spill of CN from synthesis equipment	occupational	inhalation	2	2	2	1	7	6
Product Manufacturing	PM	2	8	Accidental spill of CN from synthesis equipment	occupational	ingestion/dermal/eye	2	2	2	1	7	6
Product Manufacturing	PM	2	9	Accidental spill of CN from synthesis equipment	environmental	direct	2	2	2	1	7	6
Product Application	PA	3	5	Deposition and formulation equipment cleanout	environmental	direct	2	2	2	1	7	6
Product Application	PA	3	14	Incidental release of CN from deposition equipment	environmental	direct	2	2	2	1	7	6
Product Application	PA	3	15	Accidental spill of CN from deposition equipment	occupational	inhalation	2	2	2	1	7	6
Product Application	PA	3	16	Accidental spill of CN from deposition equipment	occupational	ingestion/dermal/eye	2	2	2	1	7	6
Product Application	PA	3	17	Accidental spill of CN from deposition equipment	environmental	direct	2	2	2	1	7	6
Product Use	PU	4	1	Membrane handling, installation, and removal	occupational	inhalation	2	3	1	1	7	6
Re-use/Recycling	RR	5	3	Composting used membrane	occupational	inhalation	2	3	1	1	7	6
Re-use/Recycling	RR	5	5	Composting used membrane	consumer	inhalation	2	3	1	1	7	6
Re-use/Recycling	RR	5	8	Bioconversion (anaerobic digestion)	occupational	inhalation	2	3	1	1	7	6
Raw Material	RM	1	1	Harvesting, chipping/shredding, pulping softwood or hardwood	occupational						0	7

Legend: LCSC, Life-Cycle Stage Code; LCSN, Life-Cycle Stage Number; SN, Scenario Number; ER, exposure route; DE, directness of exposure; M, magnitude; L, likelihood; F, frequency; RM, raw material; PM, product manufacturing; PA, product application; PU, product use; RR, re-use/recycling; D, disposal; MSW, municipal solid waste; CN, cellulose nanomaterial; TCNFs, TEMPO-oxidized cellulose nanofibers; PCCNFs, periodate-chlorite oxidized cellulose nanofibers. Colors: orange, high ranking; yellow, medium ranking; green, low ranking.

The third highest-ranking scenarios common across the evaluated CSs include potential occupational exposures in the product manufacturing and application life-cycle stages (2.1, 2.2, 3.3, 3.4, 3.8, 3.9, 3.10, 3.11). A variety of recurring cleaning and handling activities during these life-cycle stages (e.g., cleaning CN manufacturing equipment; CN formulation preparation during product manufacture; casting CN slurries for membrane, film, or packaging manufacture) may lead to inhalation, dermal, and eye exposures. Potential occupational exposure to CNs during industrial scale compositing activities (5.4) also ranked highly (Table 6).

### 3.4. High Ranking Exposure Scenarios Unique to Specific Case Studies

Exposure scenario development and ranking also identified scenarios unique to specific CSs, mainly due to differences in how the CN-containing products were manufactured, used, or recycled/re-used during end-of-life activities. High-ranking exposure scenarios are outlined in Table 7, broken out by scenarios specific to a given CS (e.g., CS1 production and use of a water filtration membrane) or group of CSs (e.g., CS2.1, 2.2, and 2.3 evaluating different applications in food contact packaging).

For CS1, potential consumer and environmental exposures ranked highly when the water filtration membranes were used past their recommended product life during the re-use/recycling life-cycle stage (5.11, 5.12). CNs released from the membranes as they degrade could result in oral exposures to consumers, or release to the environment. Potential oral exposures to consumers or workers from released CNs during product use also ranked high (4.4, 4.5), as did potential environmental exposure (4.5).

For CSs evaluating potential food contact packaging applications (CS2.1, 2.2, 2.3), scenarios with the most significant exposure potential included occupational and consumer exposures during product application, product use, and recycling life-cycle stages. These included potential dermal exposures associated with physical interactions with CN-containing packaging (e.g., physical handling during production, or shredding activities during recycling; 3.23, 4.3, 5.4, 5.6), as well as oral exposures associated with potential migration of CNs from packaging into food (4.1). In CS2.2, potential occupational inhalation and dermal/eye exposures also ranked highly during spray-coating of SCNF on food contact materials in the product application life-cycle stage (3.10, 3.11).

The most significant potential exposures specific to food additive applications of TCNF or PCCNF (CS3) were oral or dermal exposures to consumers, associated with food handling and consumption during product use (4.1, 4.2). The analysis also highly ranked potential environmental exposure to CNs during re-use/recycling activities (5.2), such as composting or converting CN-containing food waste into animal feed.

The quantitative ranking of exposure scenarios for all five CSs establishes the priorities for further characterization of potential human health or environmental risks. In the Exposure Assessment Results (Section 3.6), available evidence from the peer-reviewed literature is reviewed to refine exposure estimates for these scenarios. In the Hazard Assessment (Section 3.7 and Section 3.8), the potential oral, dermal, and inhalation hazards of TCNF, PCCNF, and SCNF are evaluated. This is accomplished by (1) using the methods and data in the SbD Toolbox to conduct a screening-level hazard assessment of TCNF, PCCNF, and SCNF to determine if these materials can be grouped together for the read-across of safety data; and (2) a review of the peer-reviewed literature to derive benchmark toxicity values for hazard assessment. In the Health and Environmental Risk Characterization (Section 4), the potential risks associated with top-ranking exposure scenarios at each life-cycle stage are characterized using the developed information on TCNF, PCCNF, and SCNF exposures and hazards.

**Table 6:** Top ranking exposure scenarios common to all five CSs.

	Exposure Scenarios							Exposure Scenario Ranking					
CS	Life-Cycle Stage	LCSC	LCSN	SN	Scenario	R	ER	DE	M	L	F	Score	Rank
**Top Ranking Exposure Scenarios**													
All CSs	Product Manufacturing	PM	2	10	Dried formulation extraction and handling (for powder CN ingredients)	O	I	3	3	3	3	12	1
All CSs	Product Manufacturing	PM	2	11	Dried formulation extraction and handling (for powder CN ingredients)	O	I/D/E	3	3	3	3	12	1
All CSs	Product Application	PA	3	1	Transfer CN from synthesis to application facility (e.g., handling, packaging)	O	I	3	3	3	3	12	1
All CSs	Product Application	PA	3	2	Transfer CN from synthesis to application facility (e.g., handling, packaging)	O	I/D/E	3	3	3	3	12	1
All CSs	Product Application	PA	3	6	CN rehydration	O	I	3	3	3	3	12	1
All CSs	Product Application	PA	3	7	CN rehydration	O	I/D/E	3	3	3	3	12	1
**Second Highest Ranked Exposure Scenarios**													
CS1, 2.1, 2.2, 2.3	Product Application	PA	3	19	CN drying (membrane/film/packaging formation)	O	I/D/E	2	3	3	3	11	2
CS1, 2.1, 2.2, 2.3	Product Application	PA	3	21	Surface treatment of CN membrane/film/packaging (e.g., functionalization, other)	O	I/D/E	2	3	3	3	11	2
All CSs	Re-use/Recycling	RR	5	2	Collection and transport of used CN product to re-use or composting facility	O	I/D/E	2	3	3	3	11	2
All CSs	Re-use/Recycling	RR	5	7	Composting used CN products	E	D	2	3	3	3	11	2
All CSs	Disposal	D	6	6	Long-term MSW landfill storage of CN products	E	D	2	3	3	3	11	2
**Third Highest Ranked Exposure Scenarios**													
All CSs	Product Manufacturing	PM	2	1	Cleaning out synthesis equipment	O	I	2	2	3	3	10	3
All CSs	Product Manufacturing	PM	2	2	Cleaning out synthesis equipment	O	I/D/E	2	2	3	3	10	3
All CSs	Product Application	PA	3	3	CN product manufacturing equipment cleanout	O	I	2	2	3	3	10	3
All CSs	Product Application	PA	3	4	CN product manufacturing equipment cleanout	O	I/D/E	2	2	3	3	10	3
All CSs	Product Application	PA	3	8	CN formulation preparation (e.g., mixing with other ingredients, homogenizing, other)	O	I	2	2	3	3	10	3
All CSs	Product Application	PA	3	9	CN formulation preparation (e.g., mixing with other ingredients, homogenizing, other)	O	I/D/E	2	2	3	3	10	3
CS1, 2.1, 2.3	Product Application	PA	3	10	CN casting to form membrane/film/packaging	O	I	2	2	3	3	10	3
CS1, 2.1, 2.3	Product Application	PA	3	11	CN casting to form membrane/film/packaging	O	I/D/E	2	2	3	3	10	3
All CSs	Re-use/Recycling	RR	5	4	Composting used CN product	O	I/D/E	2	3	2	3	10	3

Legend: LCSC, Life-Cycle Stage Code; LCSN, Life-Cycle Stage Number; SN, Scenario Number; R, receptor; ER, exposure route; DE, directness of exposure; M, magnitude; L, likelihood; F, frequency; RM, raw material; PM, product manufacturing; PA, product application; PU, product use; RR, re-use/recycling; D, disposal; MSW, municipal solid waste; O, occupational; I/D/E, ingestion, dermal, eye; I, inhalation; Ingest., ingestion; E, environmental; C, consumer; D, direct; CN, cellulose nanomaterial; TCNFs, TEMPO-oxidized cellulose nanofibers; PCCNFs, periodate-chlorite oxidized cellulose nanofibers. Colors: orange, high ranking; yellow, medium ranking.

**Table 7:** High ranking exposure scenarios unique to specific case studies.

	Exposure Scenarios							Exposure Scenario Ranking					
CS	Life-Cycle Stage	LCSC	LCSN	SN	Scenario	R	ER	DE	M	L	F	Score	Rank
**CS1 (Water Filtration Membrane; TCNF and PCCNF) Top Ranking Scenarios**													
CS1	Re-use/Recycling	RR	5	11	Continued use (degraded membrane)	C	I/D/E	2	3	3	3	11	2
CS1	Re-use/Recycling	RR	5	12	Continued use (degraded membrane)	E	D	2	3	3	3	11	2
CS1	Product Use	PU	4	4	Use of membrane to filter drinking water	O	Ingest.	2	2	3	3	10	3
CS1	Product Use	PU	4	5	Use of membrane to filter drinking water	C	Ingest.	2	2	3	3	10	3
CS1	Product Use	PU	4	6	Use of membrane to filter drinking water	E	D	2	2	3	3	10	3
**Food Contact CSs (CS2.1, 2.2, 2.3)**													
CS2.1, 2.2, 2.3	Product Application	PA	3	23	Physical treatment of CN packaging (e.g., forming, bending, other)	O	I/D/E	2	3	3	3	11	2
CS2.1, 2.2, 2.3	Product Use	PU	4	3	Food packaging handling/interaction (release)	C	I/D/E	2	3	3	3	11	2
CS2.1, 2.2, 2.3	Product Use	PU	4	1	Food packaging use (release, migration to food)	C	Ingest.	2	3	3	3	11	2
CS2.1, 2.2, 2.3	Re-use/Recycling	RR	5	4	Recycling activities: Physical breakdown of used food packaging (e.g., tearing)	O	I/D/E	2	3	3	3	11	2
CS2.1, 2.2, 2.3	Re-use/Recycling	RR	5	6	Recycling activities: Chemical breakdown of used food packaging (e.g., pulping)	O	I/D/E	2	3	3	3	11	2
**CS2.2 (Food Packaging Coating; SCNF) Top Ranking Scenarios**													
CS2.2	Product Application	PA	3	10	CN formulation applied via spraying coating on paper food contact material	O	I	2	2	3	3	10	3
CS2.2	Product Application	PA	3	11	CN formulation applied via spraying coating on paper food contact material	O	I/D/E	2	2	3	3	10	3
**CS3 (Food Additive; TCNF and PCCNF) Top Ranking Scenarios**													
CS3	Product Use	PU	4	1	Food handling and consumption	C	Ingest.	2	2	3	3	10	2
CS3	Product Use	PU	4	2	Food handling and consumption	C	D/E	2	2	3	3	10	2
CS3	Re-use/Recycling	RR	5	2	Production of food waste into animal feed	E	D	2	2	3	3	10	2

Legend: LCSC, Life-Cycle Stage Code; LCSN, Life-Cycle Stage Number; SN, Scenario Number; R, receptor; ER, exposure route; DE, directness of exposure; M, magnitude; L, likelihood; F, frequency; RM, raw material; PM, product manufacturing; PA, product application; PU, product use; RR, re-use/recycling; D, disposal; MSW, municipal solid waste; O, occupational; I/D/E, ingestion, dermal, eye; I, inhalation; Ingest., ingestion; E, environmental; C, consumer; D, direct; CN, cellulose nanomaterial; TCNFs, TEMPO-oxidized cellulose nanofibers; PCCNFs, periodate-chlorite oxidized cellulose nanofibers. Colors: orange, high ranking; yellow, medium ranking.

### 3.5. Potential Exposures Across the Product Life-Cycle

The extent of potential health risks to people or the environment vary across the product life-cycle and depend on the form (e.g., powder versus aqueous suspension) and route of exposure. During raw material and product manufacturing, cellulose pulp is refined and chemically modified to produce second-generation CNs (i.e., TCNF, PCCNF or SCNF) within enclosed tanks. Workers may be exposed to CN slurries through inhalation, dermal, or eye contact. If CN production includes a drying step, exposure to CN powders is also possible.

During product application, the CN dispersions or powders are transformed into a film by casting techniques, or by spray drying on a food packaging substrate. CNs may also be incorporated into food packaging or added directly to a food matrix. At this stage, inhalation or dermal occupational exposure to powder CNs (100 wt %), CN suspensions (up to 2 wt %), or mixtures (various wt %) may occur during handling and manufacturing activities. Additional dermal occupational exposures are likely from handling CN-containing products, and from any additional processing steps (e.g., surface treatments). During product manufacture and product use, potential inhalation and dermal exposures to workers are also possible during cleaning activities or accidental spills. These exposures may be to powder CNs (100 wt %) or CN suspensions (up to 2 wt %).

During product use, CNs have typically been manufactured into a film (up to 100% CNs) or CN-containing product (2–100 wt %). Inhalation exposures are expected to be low. Consumers are expected to be orally exposed to CNs as additives in food, or if CNs migrate from filtration membranes or food packaging into water/food. Dermal exposures to CNs are possible from typical handling of CN-containing products.

During product re-use/recycling and disposal, all receptor groups may encounter the following degradation products: individual CNs, CN composite particles, or modified CN forms resulting from reprocessing steps. Occupational exposures during collection and transport of used CN products, or during recycling/re-use activities (e.g., shredding, tearing) are possible. Environmental exposures are also likely associated with direct environmental release during composting or landfilling activities. Consumer exposures are also possible, including potential misuse of products (e.g., using degraded water filter membranes).

For the five CSs evaluated, any human or environmental exposures across the product life-cycle will be to one of two forms of functionalized CNs: their pristine form or as part of a composite. In early life-cycle stages, such as product manufacturing or product application, occupational inhalation, dermal, or eye exposures to pristine forms of functionalized CNs (i.e., powder or aqueous suspensions of TCNF, PCCNF, or SCNF) are possible, as is their accidental release to the environment. In later life-cycle stages, such as product use, recycling, or disposal, potential exposures to workers, the public, or the environment may involve functionalized CN composites.

### 3.6. Exposure Assessment Results

#### 3.6.1. Occupational Exposures

In facilities with adequate engineering controls that follow Good Manufacturing Practices (GMP), occupational exposure assessments (OEAs) have generally found low CN exposures and that current controls are adequate to mitigate exposure. Several approaches, techniques, and methodologies are available for OEAs in CN-based facilities, including: (i) exposure sampling techniques developed by the National Institute for Occupational Safety and Health (NIOSH) [28] and (ii) occupational exposure characterization guidance provided by United States Department of Agriculture (USDA) Forest Service (FS) [29]. However, there are currently no established occupational exposure limits (OEL) for functionalized CNs and unmodified CNs different from conventional celluloses [30].

Studies evaluating occupational exposures to surface-functionalized CNs during manufacturing and product use are still emerging; no studies were found evaluating occupational exposure to the functionalized CNs evaluated in this LCRA (i.e., TCNF, PCCNF, SCNF). Here, we describe available studies characterizing human exposures to first-generation cellulose materials (e.g., MFC) or other forms of CNs (e.g., cellulose nanocrystals [CNCs]) in occupational settings.

Ogura et al. (2020) evaluated occupational exposures to unmodified CNFs at a production facility during the collection of CNF powder from dried slurry and the transfer of CNF powder from large to small bags [31]. The study found release of aerosolized CNF during both tasks, but exposures were effectively mitigated by the use of a ventilation system. Aerosolized CNF concentrations, measured by carbon analysis and pyrolysis-gas chromatography-mass spectrometry (Py-GC-MS), were less than 5 mg/m^3^, aligning with the permissible exposure limit (PEL) set by Occupational Safety and Health Administration (OSHA) for bulk cellulose (respirable fraction).

Vartiainen et al. (2011) investigated workplace exposures to airborne MFC during grinding and spray drying production stages [32]. Airborne exposures generated during grinding activities were effectively removed by ventilation. During spray drying activities, no significant increase in airborne particles was measured with direct real-time instrumentation when the filtration system was operating. However, potential incidental release was identified from opening the air dryer or handling dried material.

Using direct, real-time measurements, O’Connor et al. (2014) evaluated potential occupational exposures associated with spray drying and handling/bagging activities at a CNC production plant [33]. When in operation, total airborne particulates were significantly elevated compared to background measurements. However, the exposures were to larger (micron-sized) particles and overall exposures were still low (0.05 mg/m^3^). Evaluating nanoscale particulates with a scanning mobility particle sizer (SMPS), the authors found no significant change in particle size distribution when exposure controls (e.g., ventilation) were properly implemented.

NIOSH, in collaboration with USDA Forest Product Laboratory (FPL), has conducted on-site field studies at a pilot plant facility. Researchers at NIOSH investigated workplace exposures to CNs using cesium-tagged CNC in post-synthesis and post-purification stages at the pilot plant [7]. They found that aerosolized cesium concentrations were the highest in filter-based samples collected during centrifugation, while concentrations during freeze-drying activities were nearly three orders of magnitude lower. NIOSH also evaluated potential occupational exposures to CNC from the release of CNC-composite materials during production, cutting, and milling. During these activities, NIOSH found the highest level of cesium in samples obtained within the personal breathing zone of a worker, followed by those near an extruder mixer and composite press. While NIOSH’s work elucidated broader CNC exposure patterns, quantitative measurements were not possible to more accurately evaluate exposure levels. The researchers also reported difficulties in using electron microscopy to identify CNC on cellulose-based filters.

#### 3.6.2. Releases from CN Composites

As demonstrated by the exposure scenario development and ranking exercise, occupational and consumer exposures may differ in frequency, magnitude, duration, and route, and the differences are generally CS-specific. Workers are typically exposed to repeated, low-magnitude CN exposures, while consumers are typically exposed to low levels of CN composite particles released during product use. The form of CN that people or the environment are exposed to (i.e., pristine versus CN composite particles) largely affects the relative hazard. Limited studies were found that evaluated the potential release of TCNF, PCCNF, or SCNF from composites during manufacturing, use, and re-use/recycle processes. Release studies on other forms of CN composites (e.g., CNC) are included here to help address these data gaps.

(1) Releases from Carboxylated CN Composites

No studies were found characterizing the potential release of carboxylated CNs (neither TCNF nor PCCNF) from composite materials.

(2) Releases from Sulfated CN Composites

Gong et al. (2021) evaluated the release of CNC from nanocomposite films composed of natural rubber latex and cellulose (nano)crystals (NR/CC) into aqueous solutions [34]. The CNCs were labeled with 5-(4,6-Dichlorotriazinyl) amino fluorescein (DTAF) and their release from NR/CC composites with different loadings (3, 6, 12 wt % CNC) was quantified using UV-vis spectrometry. The authors found release and migration of CNC from the NR/CC composite into various aqueous media, albeit at low levels (6–18 mg CNC released per gram of NR/CC composite). Several factors impacted the level of CNC release; higher migration was induced by higher CNC loading and by contact with acidic or alkaline media. When in contact with high ionic strength media, lower release of CNC was observed.

The potential migration of CNC from polylactic acid (PLA)-based CNC composites for active food packaging applications was evaluated by Fortunati et al. (2013) [35]. They evaluated the overall migration of film components into two liquid food simulants: 10% ethanol (an aqueous food simulant) and isooctane (a fatty food simulant). The results indicated that the overall migration from the composite was higher compared to pristine PLA in both food simulants; however, no specific migration testing to evaluate CNC migration was conducted. The authors reported that total migration from the PLA-CNC composite films was low (~75–275 µg/kg) and did not exceed the overall migration limits for plastic-based packaging in the EU (60 mg/kg).

Dhar et al. (2015) evaluated overall migration from poly(3-hydroxybutyrate; PHB)-based CNC composite films into two food simulants: isooctane and 10% ethanol [36]. The authors found at low concentrations of CNC (1–2 wt %), overall migration decreased compared to neat PHB in both ethanol 10% and isooctane. However, CNC loadings above 3 wt % resulted in higher overall migration values, attributed to poor adhesion between the hydrophilic CNC and hydrophobic PHB phases. The overall migration doubled when the PHB was loaded with 5 wt % CNC (~40 µg/kg in isooctane, and ~180 µg/kg into 10% ethanol). However, the study did not evaluate the specific migration of CNC from the PHC-CNC composite.

#### 3.6.3. Environmental Exposures, Fate, and Persistence

Studies evaluating the bioaccumulative potential, environmental persistence, and environmental fate of CNs are discussed to better evaluate potential environmental exposures across the life-cycle.

(1) Carboxylated CNs

Hossain et al. (2022) evaluated the biodegradation of TEMPO-oxidized CN superabsorbent polymer (SAP) in soil [37]. The authors found that the SAP biodegraded in the presence and absence of enzymatic-assisted (i.e., cellulase) conditions. The authors also found that crosslinking the CN SAP accelerated environmental biodegradation. In the absence of enzyme, crosslinked CN SAP degraded by 12% after 6 days, while neat CN SAP degraded by 8%. With enzyme, crosslinked CN SAP also degraded faster (around 56% after day 2) than the neat CN SAP (around 40% after day 2).

Similarly, Barajas-Ledesma et al. (2022) assessed the biodegradation rate of TEMPO-oxidized CN SAPs in soil [38]. Biodegradation was evaluated for two types of TEMPO-oxidized CN SAPs: freeze-dried and oven-dried at 50 °C. The authors found that the overall level of biodegradation after 28 days was dependent on the level of CN SAP loaded in soil, as well as the type of CN SAP (i.e., freeze- versus oven-dried). In soil treated with 0.2 wt % CN SAP, both freeze-dried and oven-dried forms degraded by 60% after 28 days of exposure. For soil treated with 1 wt % CN SAP, the freeze-dried form began degrading after 7 days of exposure, whereas oven-dried forms had slower degradation throughout the 28-day exposure. The addition of fertilizer to soil with 1 wt % SAP significantly increased the SAP degradation rate. The study suggests that TEMPO-CNs biodegrade under typical soil conditions.

(2) Sulfated CNs

The aerobic biodegradation of CNC and bulk cellulose fibers was evaluated by Kümmerer et al. (2011) using the Organisation for Economic Co-operation and Development (OECD) standard method (OECD 301D) [39]. CNC underwent 46% and 54% biodegradation by days 14 and 28, respectively, while conventional cellulose underwent 21% and 45% biodegradation. The higher degradation rate of CNC was likely due to its smaller size and higher surface area. While CNC biodegrades under aerobic conditions, the authors noted it did not meet the ‘readily biodegradable’ criteria (60% biodegradation after 28 days). O’Connor et al. (2014) also evaluated the biodegradability of CNC using OECD standard methods and found 42% biodegradation after 10 days [33].

#### 3.6.4. Exposure Assessment Limitations

There are several sources of uncertainty that limit the ability to make quantitative exposure estimates as part of this Nano LCRA. In the exposure scenario development and ranking exercise (Section 3.1, Section 3.2, Section 3.3, Section 3.4 and Section 3.5), several conservative assumptions were adopted that likely overestimated exposures, including that for occupational exposures, it is assumed that no PPE or engineering controls are implemented in the workplace to mitigate CN exposures. We also note key data gaps in our understanding of potential occupational, consumer, and environmental exposures. No data were identified characterizing occupational exposures to surface-functionalized CNs. Instead, we relied on available studies from first-generation cellulose materials or other forms of CNs. In evaluating potential consumer exposures, very few studies were identified that characterize the release of TCNF, PCCNF, or SCNF from relevant composite materials (e.g., food packaging) and our exposure assessment had to rely on related CNs (e.g., CNC) and composite materials. Together, these assumptions and data gaps represent sources of uncertainty in our exposure assessment.

### 3.7. Hazard Assessment: SbD Toolbox

A screening-level oral, dermal, and inhalation hazard assessment of TCNF, PCCNF, and SCNF was conducted using hazard screening tools in the SbD Toolbox. A literature review was then conducted to more fully characterize potential TCNF, PCCNF, and SCNF hazards across the product life-cycle (detailed in Section 3.8).

#### 3.7.1. Oral Hazard Evaluation

The NOAEL and LOAEL values of first-generation cellulose materials (i.e., MFC) and second-generation CNs (PCCNFa-d, TCNFa-d, and SCNFa-d) following simulated oral exposure to the SbD Toolbox’s intestinal triculture model were calculated. The original toxicological data are published [24] and are available in the SbD Toolbox. The NOAEL and LOAEL values were derived for each endpoint (i.e., cytotoxicity, inflammation, oxidative stress, and barrier integrity) and exposure duration (i.e., 15 min or 4 h). NOAEL values (in green) were derived based on the highest concentration able to be evaluated in the intestinal model for each material.

None of the evaluated cellulose materials induced cytotoxicity in the intestinal triculture model at either timepoint (Table 8). A similar trend was found for inflammatory markers, except for PCCNFb exposure. No adverse inflammation was observed at either timepoint. These results suggest that, following oral exposure, the differences in chemistry, charge density, and morphology between CNs do not impact their oral cytotoxicity or inflammation at the doses and exposure durations evaluated. These findings support that CNs can be grouped together for assessing potential oral cytotoxicity or pro-inflammatory endpoints. However, further assessment of PCCNFb toxicity is warranted.

Differences in induced oxidative stress were found between exposure to carboxylated (e.g., TEMPO-mediated) and sulfated forms of CNF, as well as between forms within the same chemistry (e.g., TCNFa-d and SCNFa-d; Table 9). Generally, CNs did not induce any oxidative stress at either the 15 min or 4 h timepoint. However, significant elevation was noted from 15 min of exposure to TCNFa (LOAEL = 0.4%) and SCNFa (LOAEL = 0.2%), and after 4 h of exposure to SCNFc and SCNFd (LOAELs of 0.4% and 0.2%, respectively). These results suggest that differences in surface chemistries, as well as charge density or morphology, may impact potential oxidative stress following oral exposure.

Except for TCNFa (LOAEL = 0.6%), none of the evaluated cellulose materials induced adverse changes in barrier integrity after 8 days of exposure. The physical and chemical differences between materials did not adversely impact the intestinal barrier following exposures to MFC, PCCNF, and SCNF at up to 2 wt %. These results support their grouping for assessing potential impacts to barrier integrity following oral exposure. Since significant changes in barrier integrity were noted with TCNFa exposure (LOAEL = 0.6%); this material was flagged for further evaluation.

Together, these results suggest that the differences in chemistry, charge density, and morphology of CNs do not impact their oral cytotoxicity, inflammation, or barrier integrity at the doses and exposure durations evaluated. With a few noted exceptions, these data provide evidence that first-generation cellulose materials and several second-generation CNs can be grouped together for assessing potential oral cytotoxicity, pro-inflammatory response, and barrier integrity. Grouping is less supported for evaluating potential oxidative stress following oral exposure, given differences noted between surface chemistries and physical and chemical properties of materials.

#### 3.7.2. Inhalation Hazard Evaluation

**Figure 3:**
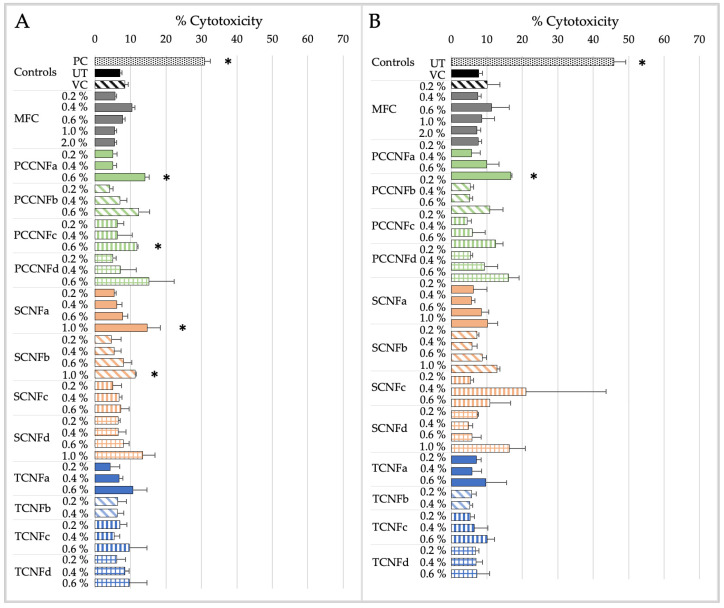
Percent cytotoxicity induced by CN exposure for (**A**) 15 min and (**B**) 4 h to SbD lung co-culture model as measured by LDH. Treatments include untreated cells (UT); simulated gastrointestinal fluid (VC: vehicle control); 1% (*v*/*v*) Triton X-100 (PC: positive control); unmodified cellulose material (MFC: unmodified cellulose fiber); periodate-chlorite surface-functionalized CNs (PCCNF); sulfonate surface-functionalized CNs (SCNF); and TEMPO surface-functionalized CNs (TCNF). Values corresponding to statistically adverse effects compared to vehicle control are denoted (* *p* < 0.05).

**Figure 4:**
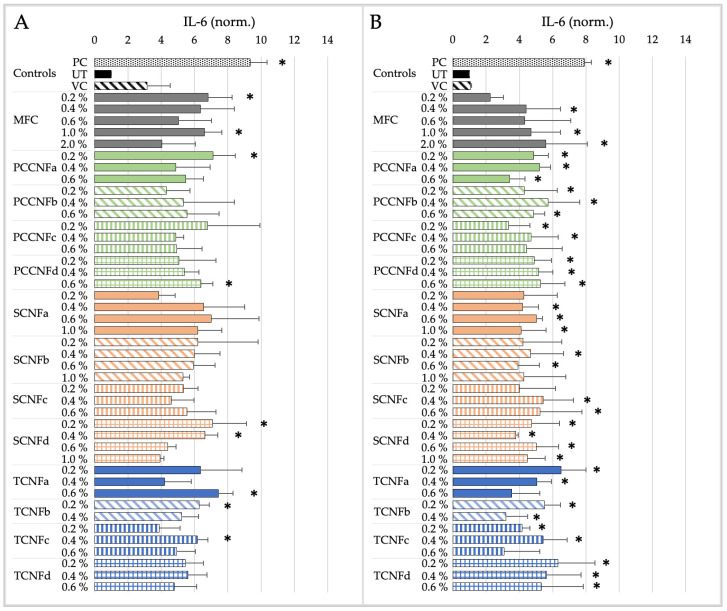
Inflammation induced by CN exposure for (**A**) 15 min and (**B**) 4 h to SbD lung co-culture model as measured by IL-6. Materials include untreated cells (UT); simulated gastrointestinal fluid (VC: vehicle control); 1% (*v*/*v*) Triton X-100 (PC: positive control); unmodified cellulose material (MFC: unmodified cellulose fiber); periodate-chlorite surface-functionalized CNs (PCCNF); sulfonate surface-functionalized CNs (SCNF); and TEMPO surface-functionalized CNs (TCNF). Values corresponding to statistically adverse effects compared to vehicle control are denoted (* *p* < 0.05).

The cytotoxicity and pro-inflammatory markers induced by MFC and second-generation CNs (PCCNFa-d, TCNFa-d, and SCNFa-d) following simulated inhalation exposure for 15 min or 4 h are shown in Figure 3 and Figure 4.

The derived NOAEL and LOAEL values for MFC and second-generation CNs (PCCNFa-d, TCNFa-d, and SCNFa-d) following simulated inhalation exposure are detailed in Table 10.

After 15 min of exposure, differences in cytotoxicity are noted between different surface chemistries (e.g., PCCNF and SCNF versus TCNF) and forms (e.g., PCCNFa,c versus PCCNFb,d). However, for longer exposure durations (i.e., 4 h), with the exception of PCCNFa (LOAEL = 0.6%), none of the evaluated materials induced any cytotoxicity following inhalation exposure (Table 10). Similar differences between materials were noted when evaluating acute (i.e., 15 min) proinflammatory responses. However, by 4 h of exposure, all cellulose materials had induced proinflammatory markers, with LOAEL values ranging from 0.2–0.4%.

The results suggest that grouping MFC and second-generation CNs (TCNF, PCCNF, and SCNF) for short-term exposures is not supported given the differences between materials. Generally, limited cytotoxicity is observed following exposure to MFC, TCNF, PCCNF, or SCNF up to 2 wt % after 4 h of exposure. However, these materials all induce pro-inflammatory markers following 4 h of inhalation exposure. These results suggest that, for longer term exposures (i.e., 4 h), these materials can be grouped together to support inhalation hazard assessments.

#### 3.7.3. Dermal Hazard Evaluation

**Figure 5:**
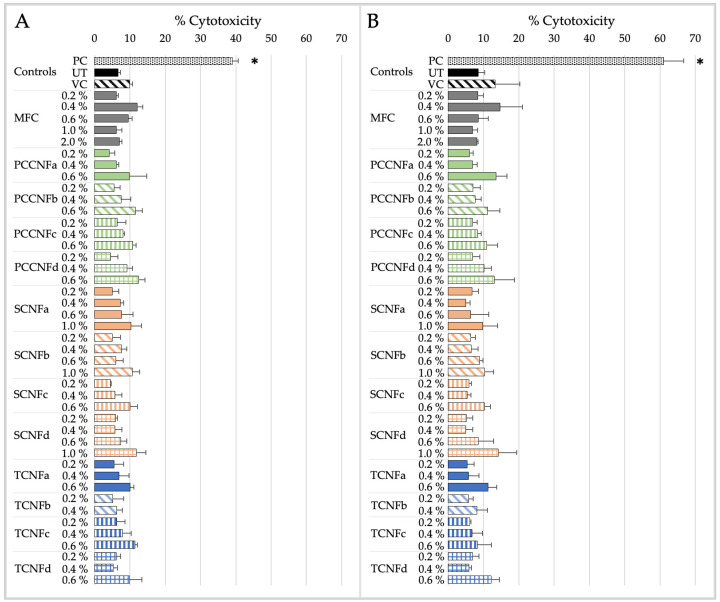
Percent cytotoxicity induced by CN exposure for (**A**) 15 min and (**B**) 4 h to SbD dermal co-culture model as measured by LDH. Materials include untreated cells (UT); simulated gastrointestinal fluid (VC: vehicle control); 1% (*v*/*v*) Triton X-100 (PC: positive control); unmodified cellulose material (MFC: unmodified cellulose fiber); periodate-chlorite surface-functionalized CNs (PCCNF); sulfonate surface-functionalized CNs (SCNF); and TEMPO surface-functionalized CNs (TCNF). Values corresponding to statistically adverse effects compared to vehicle control are denoted (* *p* < 0.05).

**Figure 6:**
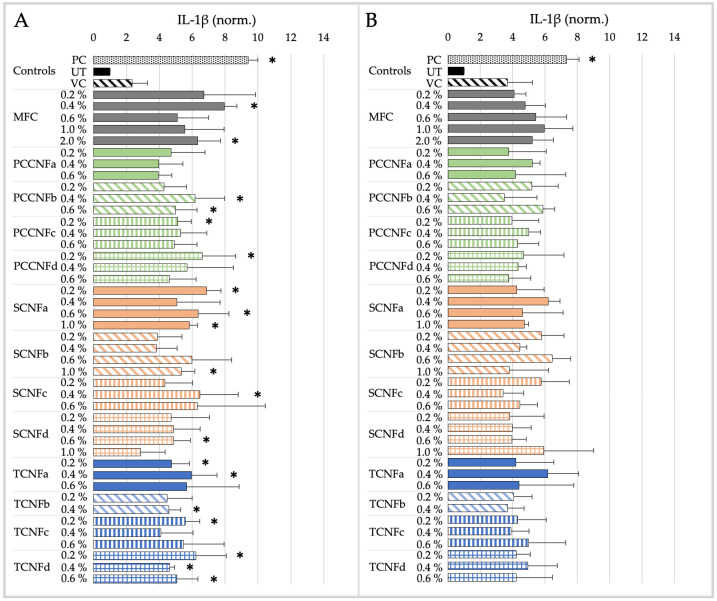
Inflammation induced by CN exposure for (**A**) 15 min and (**B**) 4 h to SbD dermal co-culture model as measured by IL-1β. Materials include untreated cells (UT); simulated gastrointestinal fluid (VC: vehicle control); 1 mM phorbol myristate acetate (PMA, PC: positive control); unmodified cellulose material (MFC: unmodified cellulose fiber); periodate-chlorite surface-functionalized CNs (PCCNF); sulfonate surface-functionalized CNs (SCNF); and TEMPO surface-functionalized CNs (TCNF). Values corresponding to statistically adverse effects compared to vehicle control are denoted (* *p* < 0.05).

The cytotoxicity and pro-inflammatory markers induced by MFC and second-generation CNs (PCCNFa-d, TCNFa-d, and SCNFa-d) following simulated dermal exposure for 15 min or 4 h are shown in Figure 5 and Figure 6.

Based on the dermal toxicity testing performed with the SbD Toolbox, the grouping of MFC together with second-generation CNs (PCCNFa-d, TCNFa-d, and SCNFa-d) is generally supported for evaluating potential cytotoxicity and pro-inflammation following dermal exposure. The derived NOAEL and LOAEL values for all materials following simulated dermal exposure (15 min and 4 h) are detailed in Table 11.

None of the cellulose materials induced cytotoxicity following 15 min or 4 h of dermal exposure. Pro-inflammatory markers were elevated following 15 min of dermal exposure to all materials (excluding PCCNFa), with LOAEL values between 0.2 and 1%. However, the inflammation was transient; after 4 h of dermal exposure, no significant increase in pro-inflammatory markers was found for any material.

#### 3.7.4. Summary: SbD Toolbox CN Hazard Assessment

The SbD Toolbox was used to conduct a screening-level toxicity assessment of second-generation CNs (TCNF, PCCNF, and SCNF) side-by-side with first-generation cellulose materials. This analysis aimed to: (i) screen for potential oral, dermal, and inhalation hazards of second-generation CNs; and (ii) evaluate whether grouping of cellulose materials is supported for endpoint-specific hazard assessments. For the concentrations and timepoints evaluated in the SbD Toolbox, the following conclusions can be made:

- Following oral exposure, cellulose materials generally do not induce cytotoxicity or inflammation, or impact barrier integrity. The differences in chemistry, charge density, and morphology (i.e., length and width) between materials does not impact their oral cytotoxicity, inflammation, or barrier integrity. With a few noted exceptions, these data provide evidence that first-generation cellulose materials (i.e., MFC) and several second-generation CNs (TCNF, PCCNF, SCNF) can be grouped together for assessing these endpoints. Grouping is less supported for evaluating potential oxidative stress following oral exposure, given differences between surface chemistries and physical and chemical properties of materials.
- Following inhalation exposure, limited cytotoxicity is observed for all cellulose materials after 4 h of exposure. However, all materials induced pro-inflammatory markers at this timepoint. The results suggest that, for longer term inhalation exposures, grouping of first-generation cellulose materials (i.e., MFC) and second-generation CNs (TCNF, PCCNF, SCNF) is supported for inhalation hazard assessments. Given discrepant cytotoxicity and inflammation noted after 15 min of exposure, grouping is less supported for this timepoint.
- No dermal cytotoxicity was found. Pro-inflammatory impacts were similar across materials, with elevated proinflammatory markers after 15 min which subsided by 4 h of exposure. The grouping of MFC with second-generation CNs (PCCNFa-d, TCNFa-d, and SCNFa-d) is generally supported for evaluating potential cytotoxicity and pro-inflammation following dermal exposures.

### 3.8. Hazard Assessment: Literature Review

A literature review was used to complement the SbD Toolbox screening hazard assessment (Section 3.7) and inform potential hazards associated with second-generation CN exposure across the product life-cycle. Summaries of published human health and environmental hazards from exposure to carboxylated and sulfated CNs are outlined in Table 12 and Table 13 with further discussion of the studies in Supplementary Materials. There were no available studies evaluating the hazards associated with PCCNFs. Hazard data are organized and reported by endpoint (e.g., pulmonary toxicity, dermal toxicity, or oral toxicity). The reported toxicity in the literature from second-generation CNs are synthesized together with the SbD screening hazard assessment to qualitatively evaluate health and environmental risks of CNs from use in food and food contact applications in Section 4.

#### CN Composite Hazards

There is a limited body of literature evaluating the potential health or ecological impacts from exposure to CN composites. In general, studies on composites suggest that: (1) NM composite particles, rather than pristine NMs, are generally released from the matrix during use, weathering, or end-of-life; (2) the matrix material is the primary determinant of toxicity; and (3) NMs may ‘modulate’ the toxicity of the matrix material, but this depends on several factors, including the type of NM (e.g., surface functionalization), the type of polymer, and the percent loading of NM [60,61]. No studies evaluating human health toxicity or ecotoxicity from exposure to CN-based composites were identified.

## 4. Discussion: Qualitative Health and Environmental Risk Characterization

The potential risks associated with the top-ranking exposure scenarios at each life-cycle stage are characterized according to available information on exposure and hazard. Here, these risks are summarized for the five CSs.

### 4.1. Risks During Raw Material and Product Manufacturing Life-Cycle Stages

The top-ranking exposure scenarios common to all five CSs (Table 6) relate to occupational activities associated with manufacture of TCNF, PCCNF, or SCNF. These include handling activities of CN powders during manufacture and transfer (e.g., handling, packaging) and transportation activities of powder CN products. Scenarios associated with worker exposure during manufacturing equipment cleanout or maintenance also ranked highly for exposure potential.

*Hazard*. The most significant potential hazard during raw material and product manufacturing life-cycle stages is inhalation of TCNF, PCCNF, or SCNF powders. The screening-level hazard assessment conducted with the SbD Toolbox suggests the inhalation hazards of first-generation cellulose materials (such as MFC) and second-generation CNs are similar, with limited cytotoxicity for longer exposure duration, but potential acute proinflammatory impacts. The similar pulmonary responses across materials following exposure supports that these materials can be grouped together to support inhalation hazard assessments. An evaluation of the literature suggests that CNs have a markedly lower pulmonary toxicity compared to other high-aspect ratio materials such as carbon nanotubes (CNTs), but acute inflammation has been documented in several studies. This suggests that CNs, as with many other poorly soluble particles, have the potential to irritate the lung [8]. Exposures evaluated in acute and subchronic inhalation studies report NOAEL and LOAEL values ranging from 0.3–2 mg/kg. However, there are no publicly available studies evaluating low-dose, chronic CN pulmonary exposures typical of the workplace. Addressing this outstanding data gap would allow a better understanding of chronic pulmonary hazards, and better characterization of risk during product manufacturing life-cycle stages where exposures to powdered forms of CN are most likely to occur.

*Exposure*. The extent of worker exposure to CNs during product manufacture depends on the design of the production equipment and transfer points (e.g., to a spray dryer) in terms of the likelihood of direct inhalation or dermal contact with CN powders. The exposure ranking exercise assumes no PPE is worn and that CNs may be released near the person performing these tasks. No studies were identified evaluating occupational exposures to TCNF, PCNF, or SCNF. Occupational exposure assessments from related materials such as MFC or S-CNC indicate that traditional engineering controls are effective in mitigating workplace CN exposures. Although there are no currently established OELs for functionalized CNs, they have been developed for bulk cellulose dust and are generally 5 mg/m^3^ (respirable fraction). Occupational exposure assessments conducted to date on related materials such as MFC or S-CNC have generally been well below this limit when engineering controls were properly implemented.

Inhalation of powdered CNs is the primary health concern identified for the five CSs. Data gaps remain in our understanding of the potential effects from frequent, low-dose, and direct exposure typical in occupational settings during the manufacturing life-cycle stage.

### 4.2. Risks During Product Application Life-Cycle Stage

In the product application life-cycle stage, the CNs are manufactured into commercial products with different intended applications. The CNs are present at this life-cycle stage as powders (100 wt % CN) or aqueous suspensions (2 wt % CN) before being incorporated into water filtration membranes (10 wt % CN), food contact packaging (5–100 wt % CN) or food (5 wt % CN).

*Hazard*. Similar to the product manufacture life-cycle stage, the most significant potential hazard during the product application life-cycle stage is inhalation of TCNF, PCCNF, or SCNF powders. Dermal exposures are also likely during this stage, associated with handling the CN-containing products during product application (e.g., casting CN films, drying CN films, etc.). The screening-level hazard assessment conducted with the SbD Toolbox suggests low dermal toxicity from exposure to TCNF, PCCNF, and SCNF materials, similar to first-generation cellulose materials, such as MFC. No cytotoxicity was observed for any material. Although acute inflammatory mediators were released, they resolved after 4 h. The similar responses following dermal exposures support that these materials can be grouped together to support dermal hazard assessments. An evaluation of the literature suggests that CNs are generally well-tolerated, non-irritating, and non-sensitizing dermally.

*Exposures*. The exposure scenarios during product application differed depending on the handling, manufacture, and intended use of the products in each of the five CSs. However, several high-ranking exposure scenarios at this life-cycle stage were common to each CS. These scenarios involved occupational inhalation or dermal/eye exposure to CNs. In most CSs, it is assumed CNs were formulated with other ingredients and/or redispersed, with the potential for inhalation (if aerosolized) or dermal exposures. During various production steps (e.g., casting membranes/films/packaging), inhalation exposures are possible if aerosolization occurs, as are dermal exposures associated with handling activities. Across all CSs, the product application stage also had high ranking inhalation and dermal exposures associated with equipment maintenance and clean-out. Unique to CS2.2, evaluating the use of SCNF as a food packaging coating, potential inhalation and dermal exposures ranked highly during spray coating activities used to apply the formulation onto food contact materials.

### 4.3. Risks During Product Use Life-Cycle Stage

During the product use phase, CNs have generally been incorporated into composite materials (e.g., water filtration membrane, food packaging). Although few studies evaluating the potential release of CNs from composite materials were identified, the potential for direct exposure to CNs is anticipated to be lower than previous life-cycle stages. The exception is CS3, where CNs are used as a food additive at up to 5 wt % and direct oral exposures by consumers are expected.

*Hazard*. No studies were identified that evaluated potential dermal or inhalation hazards from functionalized CN composites; this remains an outstanding data gap in our understanding of potential risks from CN composites generally.

During the product use life-cycle stage, oral exposures to CNs are possible from their use as a food additive (CS3), from migration from food packaging into food (CS2.1, 2.2, and 2.3), or from release into water from filtration membranes (CS1). The screening-level hazard assessment conducted with the SbD Toolbox, as well as 90 d subchronic studies, suggest low oral toxicity from exposure to TCNF, PCCNF, and SCNF materials, similar to responses observed for first-generation cellulose materials such as MFC [9]. Limited cytotoxicity, inflammation, or adverse impacts to barrier integrity were noted at the doses and exposure durations evaluated. With a few noted exceptions, these data provide evidence that first-generation cellulose materials (i.e., MFC) and several second-generation CNs (TCNF, PCCNF, SCNF) can be grouped together for assessing potential oral cytotoxicity, pro-inflammatory, or barrier integrity endpoints; the differences in chemistry, charge density, and morphology (i.e., length and width) between materials does not impact their oral toxicity. There now exists a substantial set of data demonstrating the safety of MFC for food-related applications, and this grouping supports read-across of these data for TCNF, PCCNF, and SCNF oral hazard assessments.

*Exposures*. The most likely exposures associated with product use are from applications of TCNF or PCCNF as a food additive (CS3), where direct oral exposures to consumers are expected. Few studies were identified evaluating potential releases from CN composites, such as water filtration membranes (CS1) or food packaging (CS2). However, release of CNs from composite materials into aqueous suspensions and food simulants has been documented, albeit at low release rates. While exposure is likely to be to CN composite particles, release of free CNs is possible, as demonstrated by release studies of other composite materials. Potential exposure routes during product use are primarily oral exposures associated with the consumption of CN-containing food, or oral exposure to CNs that have released and migrated from food contact materials or water filtration membranes. Dermal exposures associated with handling membranes and food contact packaging are also likely.

### 4.4. Risks During Re-Use and Recycling Life-Cycle Stages

At the end-of-life stages of re-use and recycling, CN-containing products may be used beyond their recommended lifespan (e.g., water filters). CN-containing products may be recycled, re-used (e.g., food packaging), or composted. Additionally, upcycling processes like pyrolysis and biodrying may be used to create new materials at this stage.

*Hazard*. During the re-use and recycling life-cycle stages, exposure is most likely to functionalized CN composites. No studies were identified that evaluated potential hazards from functionalized CN composite exposure, and this remains an outstanding data gap.

*Exposures*. The highest-ranking exposure scenarios during re-use and recycling involved potential occupational exposures associated with recycling or composting activities of CN-containing products. This can include potential dermal exposures if CNs are released from products during handling in the collection and transport of used CN products. Recycling and composting activities of CN-containing products involve some degree of mechanical (e.g., shredding) or chemical (e.g., pulping) processing that may result in inhalation or dermal exposures to CN or CN composites. There is uncertainty amid very limited data evaluating exposure characteristics of particles released from CN composites in general.

### 4.5. Risks During Disposal Life-Cycle Stage

*Hazard*. During disposal, exposure is most likely to CN composites. No studies were identified that evaluated potential dermal or inhalation hazards from CN composites, and this remains an outstanding data gap.

*Exposures*. Exposures to consumers or workers associated with the disposal life-cycle stage were the lowest-ranking due to their low magnitude, frequency, directness, and likelihood. Disposal is likely to occur under controlled conditions, such as incineration or landfilling. Occupational exposures are unlikely in these highly-controlled environments. However, direct environmental exposures are possible in this life-cycle stage.

### 4.6. Risks to the Environment

*Hazard*. Available data evaluating the ecotoxicity of CNs suggest low toxicity with limited adverse impacts across a number of organisms spanning several trophic levels, even at relatively high environmental concentrations (mg/L range). Cellulose is the most abundant natural polymer on earth and is expected to be well tolerated in the environment. The available studies published in the peer-reviewed literature indicate chemical functionalization to produce TCNF, PCCNF, and SCNF does not significantly impact exposure, and these forms are equally well-tolerated by the environment.

*Exposures*. The highest-ranked exposure scenarios resulting in direct environmental release were associated with the re-use/recycling and disposal life-cycle stages. In all CSs, CN-containing products can be released into the environment through composting or landfilling. However, similar to conventional forms of cellulose, carboxylated and sulfated CNs will undergo aerobic degradation in the environment and are not expected to be persistent.

Together, the low potential for environmental persistence and low ecotoxicity suggest that overall environmental risks from the use of functionalized CNs in food and food contact packaging applications are low.

## 5. Conclusions

The Nano LCRA identified and prioritized potential risks throughout the product life-cycle for second-generation CNs in five case studies, focusing on food and food contact material applications. The SbD Toolbox, a compendium of high throughput physical, chemical, and toxicological methodologies and data, was used to conduct a screening-level hazard assessment of carboxylated (TCNF, PCCNF) and sulfated (SCNF) CNs as part of the Nano LCRA. The screening level hazard assessment found:

- First and second-generation cellulose materials elicit similar cytotoxicity and pro-inflammatory responses following longer-term oral, dermal or inhalation exposures;
- At the doses and time points evaluated, cellulose materials behaved similarly and generally had limited cytotoxicity in oral, dermal and inhalation models. The materials also behaved similarly for inflammation endpoints, inducing limited pro-inflammatory mediators in the oral and dermal model, while significant pro-inflammatory mediator release was observed for all materials in the inhalation model;
- The similar biological responses of these materials provide supportive evidence that first-generation cellulose materials (i.e., MFC) and several second-generation CNs (TCNF, PCCNF, SCNF) can be grouped for oral, dermal, and inhalation hazard assessments.

The Nano LCRA demonstrated how the screening level hazard assessments conducted with the SbD Toobox’s methods and data were then incorporated into the risk assessment for an early, pre-commercial evaluation of safety of TCNF, PCCNF, and SCNF materials used in five food and food contact applications. Major findings included:

- The exposure scenarios with the most direct and highest potential exposure to CNs were occupational activities during the product manufacturing and product application life-cycle stages. While these scenarios are the most controllable, no studies were identified evaluating potential hazards from low-dose, chronic exposure to CNs typical of the workplace. The screening level hazard assessment conducted with the SbD Toolbox highlighted potential inflammation from CN inhalation exposure, which was supported by studies evaluated in the literature which suggest CNs behave as poorly soluble, low-toxicity dusts with the potential to irritate the lung when inhaled;
- Once the CNs are incorporated into composite materials (e.g., water filtration membranes, food contact packaging) during the product application life-cycle stage, the primary exposure route is dermal and exposures are not likely to be to pristine CNs, but rather to CN composite particles. Our literature review found there is very limited data evaluating the migration or release of CNs from composites, and no studies were identified that evaluated the toxicity of CN composite particles. The exception is the use of CNs as a food additive, where direct consumer oral exposure is expected to occur. The screening level hazard assessment conducted with the SbD Toolbox suggests low oral toxicity from exposure to TCNF, PCCNF, and SCNF materials, and supported that these materials can be grouped with first-generation cellulose materials such as MFC, which have been demonstrated to be safe for oral consumption at high levels of the diet (e.g., up to 4 wt %) [9];
- There are limited risks to the environment from use of second-generation CNs in food and food contact packaging applications. These materials are biodegradable and therefor unlikely to accumulate in the environment; have limited potential to bioaccumulate; and have low ecotoxicity.

The methods and data in the SbD Toolbox can be used to evaluate the risks of new CNs early in product development and encourage the development of materials that are safer-by-design. The high-throughput physical, chemical, and toxicological new approach methodologies in the SbD Toolbox can be applied to conduct screening-level hazard assessments of new CNs, and integrated into a comprehensive Nano LCRA that evaluates potential risks across the product life-cycle early in commercial development. The SbD Toolbox employs physical, chemical, and new approach methods to demonstrate whether CNs are sufficiently similar to be grouped following established guidance for NMs. If groupings can be justified, future safety testing may be conducted on a single ‘representative’ material. This approach enables a more resource-efficient pre-commercial safety screening to promote responsible commercialization of second-generation CNs. By assessing the safety of emerging materials before their commercial adoption, this proactive approach can help mitigate harm to human health or the environment and be used to promote responsible commercialization of new cellulose materials.

## Figures and Tables

**Table 1:** Summary of CN synthesis conditions and physical and chemical properties.

	Reagent, Concentration (mmol/g), Time (min)	BT (min)	Length (nm)	Width (nm)	Charge (mmol/g)
PCCNFa	NaIO_4_, 3.08, 4; NaClO_2_, 6.16, 30	30	452 ± 162	5.9 ± 1.6	0.72
PCCNFb	NaIO_4_, 3.08, 4; NaClO_2_, 6.16, 9 h	30	533 ± 275	5.7 ± 1.6	0.82
PCCNFc	NaIO_4_, 3.08, 4; NaClO_2_, 6.16, 12 h	30	381 ± 150	5.5 ± 1.6	0.91
PCCNFd	NaIO_4_, 4.62, 4; NaClO_2_, 6.16, 6 h	30	344 ± 170	5.6 ± 1.7	1.04
TCNFa	NaClO, 3, 30	30	551 ± 200	6.5 ± 2.2	1.1
TCNFb	NaClO, 5, 50	10	486 ± 174	6.1 ± 1.6	1.42
TCNFc	NaClO, 5, 60	30	530 ± 145	4.6 ± 1.6	1.42
TCNFd	NaClO, 8, 80	30	486 ± 206	4.9 ± 2.0	1.48
SCNFa	HSO_3_Cl, 1, 30	30	693 ± 330	3.2 ± 0.9	1.49
SCNFb	HSO_3_Cl, 1.25, 45	30	577 ± 294	4.0 ± 1.0	1.84
SCNFc	HSO_3_Cl, 1.5, 60	5	501 ± 295	3.2 ± 0.7	2.23
SCNFd	HSO_3_Cl, 1.5, 60	30	365 ± 194	NA	2.23

Legend: BT, blending time.

**Table 2:** Priority second-generation CN chemistries for safety assessment in Nano LCRA.

Form	Functional Group	Regioselectivity	CN Form	Reaction	Reagent
**Chemically Unmodified Cellulose Materials**					
MFC	Cellulose-OH	NA	MFC	None	None
**Chemically Modified CNs**					
Sulfated	Cellulose-OSO_3_	None	SCNF	Sulfation	Chlorosulfonic Acid
Carboxylated (TEMPO)	Cellulose-COOH	C6	TCNF	Oxidation	TEMPO
Carboxylated (PC)	Cellulose-COOH	C2 and C3	PCCNF	Oxidation	Periodate/Chlorite (P/C)

**Table 3:** Life-cycle stages and adopted assumptions for five case studies (CSs).

CS Applications	Water Filtration	Food Contact			Food Additive
	CS1	CS2.1	CS2.2	CS2.3	CS3
**Scenario**	Water Filtration Membrane	Food Packaging Film	Food Packaging Coating	Food Packaging Additive	Food Additive
	Manufacture and use of a CNF membrane for water filtration.	Manufacture and use of a CNF food packaging film.	Manufacture and use of a CNF barrier coating applied to food contact packaging.	Manufacture and use of CNF as a food packaging additive.	Manufacture and use of CNF as a food additive.
**CNF** **Chemistries**	TCNF and PCCNF (Carboxylated)	TCNF and PCCNF (Carboxylated)	SCNF (Sulfated)	SCNF (Sulfated)	TCNF and PCCNF (Carboxylated)
**All Life-Cycle Stages**	In evaluating potential exposures across product life, assumes no Personal Protective Equipment (PPE) is used. Nano LCRA evaluates the safety of the CN; the safety of other ingredients and materials are not considered.				
**Raw Material**	Harvesting, chipping/shredding, pulping of hardwood or softwood for CN production.				
**Product** **Manufacturing**	CN is produced in an enclosed batch reactor. CN produced as a 2% suspension/gel. Manufacture includes a drying step to produce a re-dispersible powder. CN powder is handled, bagged, and shipped. Incidental release of CN powder possible from manufacturing or drying equipment. Accidental spill of CN during manufacture possible. CN manufacture takes place indoors with limited environmental release.				
	Follows conventional carboxylation steps (TEMPO or P/C oxidized) for CN.	Follows conventional carboxylation steps (TEMPO or P/C oxidized) for CN.	Follows conventional sulfation steps for CN.		Follows conventional carboxylation steps (TEMPO or P/C oxidized) for CN.
**Product** **Application**	Shipping/transportation of CN to product application facility. Redispersion of CN powder required as part of product application; powder exposures possible during handing. Incidental release and accidental spill of CN during product application is possible. Product application takes place indoors with limited environmental release.				
	CN formulation is prepared (e.g., mixing with other ingredients, etc.) at <2 wt % aqueous solution. CN formulation is cast to form a membrane. Membrane is dried prior to handling; membrane is >10 wt % CN. CN membrane may be treated to improve absorption capability.	CN formulation is prepared (e.g., mixing with other ingredients, etc.) at <2 wt % aqueous solution. Assume CN is cast at <2 wt % to form a food packaging film. CN film is dried prior to handling and may be up to 100 wt % CN. Optional finishing steps of CN film include hot pressing, embossing, and folding, as well as application of potential coatings.	CN coating formulation is prepared (e.g., mixing with other ingredients, etc.) at <2 wt % aqueous solution. CN coating is applied to conventional food contact paper/board using spray coating. Coating is left to dry/cure and may be up to 100% CN. Optional finishing step of the CN coating includes hot pressing and folding.	CNs are mixed into traditional ingredients used to make food contact paper/board (e.g., pulp). Assumes traditional papermaking methods are used (e.g., screening, pressing, and drying steps). Optional finishing steps for CN paper/board may include hot pressing, embossing, or folding.	CNs are incorporated into food for a variety of technical effects (e.g., stabilizer, emulsifier, thickener, calorie reducer). CNs added in up to 5 wt % using high shear mixing or similar processes in an open system.
**Product Use**	Membrane handling during installation. Membrane has a pre-defined commercial lifetime. CN membrane is primarily used for water filtration intended for consumption. CN membrane may be used for large-scale water filtration (e.g., occupational exposures) or for direct use by consumers.	Single-use food packaging film in direct contact with food. Misuse/degradation of the food packaging film is possible.	Single-use food contact paper/board coated with CN in direct contact with food. Misuse/degradation of the CN coated food contact paper/board is possible.	Single-use food contact paper/board containing up to 5 wt % CN in direct contact with food. Misuse/degradation of the CN containing food contact paper/board is possible.	Assumes CN-containing foods are handled and consumed typically by consumers.
**Re-Use/** **Recycling**	Shipping/transportation of CN products to re-use/recycling/composting facility. Assumes direct contact with the CN products during re-use/recycling/composting process is limited, and that these processes take place in an open system.				
	Composting may be done at consumer or industrial scale. Re-use is only expected to happen once in lifetime of CN filter membrane before entering disposal stage. Bioconversion processes may include chemical breakdown with biological processes or agents.	Recycling activities may include physical (e.g., tearing) and/or chemical (e.g., pulping) breakdown. Composting may be done at consumer or industrial scale. Bioconversion processes may include chemical breakdown with biological processes or agents.	Recycling activities may include physical (e.g., tearing) and/or chemical (e.g., pulping) breakdown. Composting may be done at consumer or industrial scale. Bioconversion processes may include chemical breakdown with biological processes or agents.	Recycling activities may include physical (e.g., tearing) and/or chemical (e.g., pulping) breakdown. Composting may be done at consumer or industrial scale. Bioconversion processes may include chemical breakdown with biological processes or agents.	Assumes food waste is primarily re-used for animal feed or for composting at consumer or industrial scale. Bioconversion processes may include chemical breakdown with biological processes or agents.
**Disposal**	Transportation of discarded CN products to end-of-life location. Primary disposal facilities include (1) long-term municipal solid waste landfill or (2) incineration in waste-to-energy facility. Assumes some CN products are discarded in environment (e.g., litter).				

**Table 4:** Exposure criteria for scenario ranking.

	Directness of Exposure	Magnitude	Likelihood	Frequency
Low (1)	Covalently bound CN in substrate	Exposure to an article where one component is <1% CN	Direct contact mitigated	Infrequent—exposure possible < 10 times per year
Medium (2)	CN potentially releasable from substrate	Exposure to a material 1% < CN < 10%	Unintentional—exposure possible based on activity	Incidental—use 10–50 times per year
High (3)	Dried CN in powder form	Exposure to a material >10% CN	Intentional—repeat exposure during normal use	Regular—greater than 50 times per year

**Table 8:** Cytotoxicity and inflammation NOAEL/LOAEL values for CNs following exposure to SbD intestinal tri-culture model.

NOAEL/LOAEL Value	Cytotoxicity		Inflammation	
	15 min	4 h	15 min	4 h
MFC	NOAEL > 2%	NOAEL > 2%	NOAEL > 2%	NOAEL > 2%
PCCNFa	NOAEL > 0.6%	NOAEL > 0.6%	NOAEL > 0.6%	NOAEL > 0.6%
PCCNFb	NOAEL > 0.6%	NOAEL > 0.6%	LOAEL = 0.2%	NOAEL > 0.6%
PCCNFc	NOAEL > 0.6%	NOAEL > 0.6%	NOAEL > 0.6%	NOAEL > 0.6%
PCCNFd	NOAEL > 0.6%	NOAEL > 0.6%	NOAEL > 0.6%	NOAEL > 0.6%
TCNFa	NOAEL > 0.6%	NOAEL > 0.6%	NOAEL > 0.6%	NOAEL > 0.6%
TCNFb	NOAEL > 0.4%	NOAEL > 0.4%	NOAEL > 0.4%	NOAEL > 0.4%
TCNFc	NOAEL > 0.6%	NOAEL > 0.6%	NOAEL > 0.6%	NOAEL > 0.6%
TCNFd	NOAEL > 0.6%	NOAEL > 0.6%	NOAEL > 0.6%	NOAEL > 0.6%
SCNFa	NOAEL > 1%	NOAEL > 1%	NOAEL > 1%	NOAEL > 1%
SCNFb	NOAEL > 1%	NOAEL > 1%	NOAEL > 1%	NOAEL > 1%
SCNFc	NOAEL > 0.6%	NOAEL > 0.6%	NOAEL > 0.6%	NOAEL > 0.6%
SCNFd	NOAEL > 1%	NOAEL > 1%	NOAEL > 1%	NOAEL > 1%

Legend: green, NOAEL; orange, LOAEL.

**Table 9:** Oxidative stress and barrier integrity NOAEL/LOAEL values for CNs following exposure to SbD intestinal tri-culture model.

NOAEL/LOAEL Value	Oxidative Stress		Barrier Integrity
	15 min	4 h	8 Days
MFC	NOAEL > 2%	NOAEL > 2%	NOAEL > 2%
PCCNFa	NOAEL > 0.6%	NOAEL > 0.6%	NOAEL > 0.6%
PCCNFb	NOAEL > 0.6%	NOAEL > 0.6%	NOAEL > 0.6%
PCCNFc	NOAEL > 0.6%	NOAEL > 0.6%	NOAEL > 0.6%
PCCNFd	NOAEL > 0.6%	NOAEL > 0.6%	NOAEL > 0.6%
TCNFa	LOAEL = 0.4%	NOAEL > 0.6%	LOAEL = 0.6%
TCNFb	NOAEL > 0.4%	NOAEL > 0.4%	NOAEL > 0.4%
TCNFc	NOAEL > 0.6%	NOAEL > 0.6%	NOAEL > 0.6%
TCNFd	NOAEL > 0.6%	NOAEL > 0.6%	NOAEL > 0.6%
SCNFa	LOAEL = 0.2%	NOAEL > 1%	NOAEL > 1%
SCNFb	NOAEL > 1%	NOAEL > 1%	NOAEL > 1%
SCNFc	NOAEL > 0.6%	LOAEL = 0.4%	NOAEL > 1%
SCNFd	NOAEL > 1%	LOAEL = 0.2%	NOAEL > 1%

Legend: green, NOAEL; orange, LOAEL.

**Table 10:** Cytotoxicity and inflammation NOAEL/LOAEL values for CNs following exposure to SbD lung co-culture model.

NOAEL/LOAEL Value	Cytotoxicity		Inflammation	
	15 min	4 h	15 min	4 h
MFC	NOAEL > 2%	NOAEL > 2%	LOAEL = 1%	LOAEL = 0.4%
PCCNFa	LOAEL = 0.6%	LOAEL = 0.6%	LOAEL = 0.2%	LOAEL = 0.2%
PCCNFb	NOAEL > 0.6%	NOAEL > 0.6%	NOAEL > 0.6%	LOAEL = 0.2%
PCCNFc	LOAEL = 0.6%	NOAEL > 0.6%	NOAEL > 0.6%	LOAEL = 0.2%
PCCNFd	NOAEL > 0.6%	NOAEL > 0.6%	LOAEL = 0.6%	LOAEL = 0.2%
TCNFa	NOAEL > 1%	NOAEL > 0.6%	LOAEL = 0.6%	LOAEL = 0.2%
TCNFb	NOAEL > 0.4%	NOAEL > 0.4%	LOAEL = 0.2%	LOAEL = 0.2%
TCNFc	NOAEL > 1%	NOAEL > 0.6%	LOAEL = 0.4%	LOAEL = 0.2%
TCNFd	NOAEL > 1%	NOAEL > 0.6%	NOAEL > 0.6%	LOAEL = 0.2%
SCNFa	LOAEL = 1%	NOAEL > 1%	NOAEL > 1%	LOAEL = 0.4%
SCNFb	LOAEL = 1%	NOAEL > 1%	NOAEL > 1%	LOAEL = 0.4%
SCNFc	NOAEL > 0.6%	NOAEL > 0.6%	NOAEL > 0.6%	LOAEL = 0.4%
SCNFd	NOAEL > 1%	NOAEL > 1%	LOAEL = 0.2%	LOAEL = 0.2%

Legend: green, NOAEL; orange, LOAEL.

**Table 11:** Cytotoxicity and inflammation NOAEL/LOAEL values for CNs following exposure to SbD dermal co-culture model.

NOAEL/ LOAELValue	Cytotoxicity		Inflammation	
	15 min	4 h	15 min	4 h
MFC	NOAEL > 2%	NOAEL > 2%	LOAEL = 0.4%	NOAEL > 2%
PCCNFa	NOAEL > 0.6%	NOAEL > 0.6%	NOAEL > 0.6%	NOAEL > 0.6%
PCCNFb	NOAEL > 0.6%	NOAEL > 0.6%	LOAEL = 0.4%	NOAEL > 0.6%
PCCNFc	NOAEL > 0.6%	NOAEL > 0.6%	LOAEL = 0.2%	NOAEL > 0.6%
PCCNFd	NOAEL > 0.6%	NOAEL > 0.6%	LOAEL = 0.2%	NOAEL > 0.6%
TCNFa	NOAEL > 0.6%	NOAEL > 0.6%	LOAEL = 0.2%	NOAEL > 0.6%
TCNFb	NOAEL > 0.4%	NOAEL > 0.4%	LOAEL = 0.4%	NOAEL > 0.4%
TCNFc	NOAEL > 0.6%	NOAEL > 0.6%	LOAEL = 0.2%	NOAEL > 0.6%
TCNFd	NOAEL > 0.6%	NOAEL > 0.6%	LOAEL = 0.2%	NOAEL > 0.6%
SCNFa	NOAEL > 1%	NOAEL > 1%	LOAEL = 0.2%	NOAEL > 1%
SCNFb	NOAEL > 1%	NOAEL > 1%	LOAEL = 1%	NOAEL > 1%
SCNFc	NOAEL > 0.6%	NOAEL > 0.6%	LOAEL = 0.4%	NOAEL > 0.6%
SCNFd	NOAEL > 1%	NOAEL > 1%	LOAEL = 0.6%	NOAEL > 1%

Legend: green, NOAEL; orange, LOAEL.

**Table 12:** Benchmark toxicity values for carboxylated CNs.

Form	Endpoint and Model	Result	Benchmark	Reference
1. Human Health—Acute Toxicity				
(i) Inhalation				
TCNF	Female C57BL/6 mice; 1-, 28-, and 90-day toxicity	TCNF at the dose of 56 µg/mouse/aspiration significantly increased the neutrophil counts in bronchoalveolar lavage fluid (BALF) on day 1.	1-day LOAEL for neutrophil influx: 50 µg/mouse/aspiration	[40]
TCNF	Female C57BL/6 mice; 24 h (OECD 474, OECD 489)	TCNF significantly induced DNA damage in lung cells, increased the recruitment of inflammatory cells to the lungs, and increased mRNA expression of tumor necrosis factor α, IL-1β, IL-6, and chemokine (C-X-C motif) ligand 5 in a dose-dependent manner, but had no effects on the bone marrow micronucleus.		[41]
Carboxylated CNF	Female C57BL/6 mice; 24 h	Carboxylated CNF at 40 µg/mouse triggered influx of neutrophils into BAL and elevated the mRNA expression of IL-6 and IL-13 in the lung tissue 24 h after treatment, but had no effects on eosinophils, IL-1β, and Tumor Necrosis Factor (TNF).	24-h LOAEL for IL-6 and IL-13 mRNA: 40 µg/mouse	[42]
Carboxylated CNF	Female C57BL/6 mice; 24 h	Exposure to 0.9 mg/kg bw/day of CNF at day 1 post-exposure significantly induced total neutrophil influx into BAL fluid and systemic Serum Amyloid A3 (SAA3) levels	24-h NOAEL for pulmonary inflammation: 0.3 mg/kg bw/day	[43]
TCNF	Sprague-Dawley rats; 2 days (OECD 474)	No difference between the proportion of micronucleated bone marrow polychromatic erythrocytes (MNPCEs) in bone marrow from exposure to TCNF compared to the negative control group	NOAEL for MNPCEs: 1.0 mg/kg	[44]
TCNF	Co-culture of A549 and monocyte-derived macrophage (THP-1) cells; 24 or 48 h (OECD 487)	No proinflammatory cytokine IL-1β was detected in the co-culture suggesting no immunotoxicity. TCNF treatment did not induce sizable levels of DNA damage in A549 cells, but it led to micronuclei formation at 1.5 and 3 μg/cm^2^	NOAEL for immunotoxicity and DNA damage: 25 µg/cm^2^	[45]
(ii) Oral				
TCNF	Male C57BL/6J mice; up to 2 h	TCNF (1.2 mmol/g carboxyl content and 120 aspect ratio) exposure significantly reduced the postprandial blood glucose, plasma insulin, GIP, and triglyceride concentrations.		[46]
(iii) Dermal				
TCNF	HEKa and HDFa; up to 24 h (ISO 10993-5)	TCNF had no effects on cytotoxicity and cytokine induction up to 50 µg/mL but slightly decreased metabolic activity.	24-h NOAEL for cytotoxicity and cytokine induction: 50 µg/mL	[47]
(iv) Eye				
No data available				
2. Human Health—Subchronic and Chronic Toxicity				
(i) Inhalation				
TCNF	Female C57BL/6 mice; 1-, 28-, and 90-day toxicity	TCNF significantly increased neutrophil counts in BAL on day 28 (80 and 200 µg/mouse/aspiration) and day 90 (200 µg/mouse/aspiration). TCNF at doses of 14, 28, and 56 µg/mouse/aspiration significantly increased DNA damage in BAL at 90 days.	90-day LOAEL for neutrophil influx: 50 µg/mouse/aspiration. 28-day LOAEL for neutrophil influx: 28 µg/mouse/aspiration. 90-day LOAEL for DNA damage: 14 µg/mouse/aspiration.	[40]
Carboxylated CNF	Female C57BL/6 mice; 28 days	Carboxylated CNF induced modest immune responses after 28 days, indicated by a slight increase in the number of neutrophils and lymphocytes in lung tissue. However, the effects were markedly attenuated as compared with the ones after 24 h.		[42]
Carboxylated CNF	Female C57BL/6 mice; 28 days	Exposure to 0.3 mg/kg bw/day of CNF at day 28 post-exposure significantly induced total neutrophil influx into BAL fluid, but had no effects on systemic SAA3 levels.	28-day NOAEL for SAA: 0.9 mg/kg bw/day	[43]
TCNF	Male Sprague-Dawley rats; 90 days	BALF analysis, histopathological examination, and a comprehensive gene expression analysis confirmed acute inflammation following the instillation of TCNFs.		[48]
3. Ecotoxicity				
TCNF	*Daphnia magna*: 24, 48, and 96 h (OECD TG 202)	No acute toxicity was observed at any given concentrations.	LC_50_ > 100 mg/L EC_50_ > 100 mg/L	[49]
	*Oryzias latipes*; 24, 48, and 96 h (OECD TG 203)	No acute toxicity was observed at any given concentrations.	LC_50_ > 100 mg/L EC_50_ > 100 mg/L	
TCNF	*Raphidocelis subcapitata*; 72 h (OECD 201)	No growth inhibition was observed at all concentrations tested (up to 100 mg/L).	72 h-EC_50_ of growth inhibition > 100 mg/L	[50]
TCNF	*Mytilus galloprovincialis*; 48 and 96 h	No effects on oxidative stress or biotransformation were observed in the digestive glands and gills. The destabilization of lysosomal membranes of hemocytes, the inhibition of P-glycoprotein efflux activities in the gills and the inhibition of cholinergic enzymes (ASCh–ChE) activities in hemocytes, gills, and digestive glands were observed.		[51]
TCNF	*Paracentrotus lividus* and *Arbacia lixula*; 48 h	TCNF leachate exposure inhibited sea urchin embryo development, decreased sperm fertilizing capability and egg fertilization competence, and significantly reduced sperm motility. In *A. lixula* spermatozoa, a significant increase in the intracellular hydrogen peroxide level has been recorded. In *P. lividus* eggs, the MMP values significantly increased.		[52]
TCNF	Zebrafish embryo; 5 days	No mortality or effects on the incidence rate of pericardial and yolk sac edema were observed from TCNF exposure at a concentration of 250 mg/L.	5-day LC_50_ > 100 mg/L. 5-day EC_50_ of pericardial edema > 250 mg/L. 5-day EC_50_ of yolk sac edema > 250 mg/L.	[53]

**Table 13:** Benchmark toxicity values for sulfated CNs.

Form	Endpoint and Model	Result	Benchmark	Reference
1. Human Health—Acute Toxicity				
(i) Inhalation				
S-CNC	Rats; 4 h (OECD 403)	No mortality, gross toxicity, adverse effects, or abnormalities in rats exposed to 0.26 mg/L of S-CNC for 4 h was observed.	NOAEL: 0.26 mg/L	[33]
CNC	Female C57BL/6 mice; 24 h	Exposure to CNC led to an innate inflammatory response, as evidenced by an increase in the number of leukocytes and eosinophils recovered by BAL. Exposure to CNC induced lung damage (LDH) and oxidative stress (formation of protein carbonyls and elevation of 40 hydroxynonenal).		[54]
S-CNC	Triple cell co-culture model of the human epithelial airway barrier; 24 h	No significant cytotoxicity, induction of oxidative stress, or pro-inflammatory response at any concentrations was observed.	NOAEL: 1.57 µg/cm^2^	[55]
S-CNC	3D triple cell coculture model of THP-1, JAWSII, and 16 Human Bronchial Epithelial Cells, Clone 14o (16HBE14o-); 24 h	Apical cytotoxicity was observed at concentrations of 15 and 30 mg/L, but no basolateral cytotoxicity at any dose examined. A small elevation of pro-inflammatory chemokine at the highest dose tested (30 mg/L).		[56]
(ii) Oral				
S-CNC	Sprague-Dawley rats; 7 and 14 days (OECD 407)	No adverse effects on cytotoxicity, metabolic activity, membrane permeability, oxidative stress, and proinflammatory responses from oral CNC exposure in rats up to 4% of the diet.	NOAEL (male) > 2085.3 mg/kg/day. NOAEL (female) > 2682.8 mg/kg/day	[57]
S-CNC	Rats; 14 days (OECD 425)	No effects were observed at the highest concentration tested. Not considered to present a significant hazard if swallowed.	14-day, one-time dose LD_50_ > 2000 mg/kg	[33]
(iii) Dermal				
S-CNC	Rabbits; 4 h (OECD 404)	No corrosive effects were observed from a single dose of S-CNC (0.5 g) for 4 h on the skin of an albino rabbit.		[33]
S-CNC	Guinea pigs; Acute (OECD 406)	S-CNC was found to be non-sensitizing at a single dose of 1.1 mg/mL (intradermal) and 103 mg/mL (topical induction and challenges phase).		
S-CNC	Mice; 6 days (OECD 429)	S-CNC was not considered to be a contact dermal sensitizer at concentrations of <10.7%.		
(iv) Eye				
No data available				
2. Human Health—Subchronic and Chronic Toxicity				
(i) Inhalation				
S-CNC	Male and female C57BL/6 mice; 3 weeks	Exposure resulted in pulmonary inflammation and damage, elevated oxidative stress, increased TGF-β and collagen levels in lung, and impaired pulmonary functions.		[58]
(ii) Oral				
S-CNC	Rats; 28 days (OECD 407)	No toxicity was observed at any dose. All parameters (neurological, body weight, weight gain, and food consumption) were not different from control.	28-day NOAEL > 2000 mg/kg/day	[33]
S-CNC	Sprague-Dawley rats; 90 days (OECD 408)	No adverse effects on cytotoxicity, metabolic activity, membrane permeability, oxidative stress, and proinflammatory responses from oral CNC exposure in rats up to 4% of the diet.	NOAEL (male) > 2085.3 mg/kg/day. NOAEL (female) > 2682.8 mg/kg/day	[57]
(iii) Dermal				
No data available				
(iv) Eye				
No data available				
3. Ecotoxicity				
S-CNC	*D. magna*; 48 h		48-h LC_50_ > 1 g/L	[59]
	*Ceriodaphnia dubia*; 48 h		48-h LC_50_ > 1 g/L	
	Rainbow trout; 96 h		96-h LC_50_ > 1 g/L	
	Fathead minnow; 10 days	Exposure to 0.3–0.24 g/L of S-CNC for 10 days had no effects on the cumulative egg production. S-CNC at 0.48 g/L significantly reduced egg production.	10-day IC_50_ of reproduction: 0.29 g/L	
	Zebrafish embryo; 96 h		96-h LC_50_ > 6 g/L 96-h IC_50_ of delayed hatching > 6 g/L	
	Rainbow trout hepatocytes; 48 h		48-h EC_50_ of cell viability: 245 mg/L. 48-h EC_50_ of total sugars: 23 mg/L. 48-h EC_50_ of lipid peroxidation: 434 mg/L. 48-h EC_50_ of available zinc > 2000 mg/L. 48-h EC_50_ of heat shock proteins > 2000 mg/L. 48-h EC_50_ of DNA strand break > 2000 mg/L	
	*Vibrio fischeri*; 15 min		15-min IC_25_ of luminescence > 10 g/L	
	*Pseudokirchneriella subcapitata*; 72 h		72-h IC_25_ of cell growth > 2.5 g/L	
	*Thamnocephalus platyurus*; 24 h		24-h LC_50_: 13.2 g/L	
	*Hydra attenuate*; 96 h		96-h LC_50_: 14.22 g/L (batch 4 of CNC at pH 6.8). 96-h LC_50_ > 6.8 g/L (batch 5b of CNC at pH 6.4). 96-h EC_50_ of morphological anomalies: 2.6 g/L (batch 4 of CNC at pH 6.8). 96-h EC_50_ of morphological anomalies > 6.8 g/L (batch 5b of CNC at pH 6.4)	
S-CNC	Zebrafish embryo; 5 days	No significant sublethal impacts of S-CNC on developing zebrafish were found at 200 mg/L for any of the 19 sublethal impact endpoints assessed.	5-day EC_50_ > 200 mg/L	[53]

Legend: S-CNC, sulfated CNC.

## Data Availability

The data presented in this study are openly available in the CN Safer-by-Design-Toolbox on the Cellulose Safety Resources section of the Vireo Advisors, LLC website at https://www.vireoadvisors.com/cn-material-safety-resources (accessed on 22 January 2025).

## Supplementary Materials

The following supporting information can be downloaded at: https://www.mdpi.com/article/10.3390/nano15030238/s1, Table S1: Assumptions adopted for CS1: water filtration membrane [TCNF, PCCNF (carboxyl functionalization)]; Table S2: Assumptions adopted for CS2.1: food packaging film [TCNF, PCCNF (carboxyl functionalization)]; Table S3: Assumptions adopted for CS2.2: food packaging coating (SCNF); Table S4: Assumptions adopted for CS2.3: food packaging additive (SCNF); Table S5: Assumptions adopted for CS3: food additive [TCNF, PCCNF (carboxyl functionalization)]; Table S6: Exposure scenarios for CS1: water filtration membrane [TCNF, PCCNF (carboxyl functionalization)]; Table S7: Exposure scenarios for CS2.1: food packaging film [TCNF, PCCNF (carboxyl functionalization)]; Table S8: Exposure scenarios for CS2.2: food packaging coating (SCNF); Table S9: Exposure scenarios for CS2.3: food packaging additive (SCNF); Table S10: Exposure scenarios for CS3: food additive [TCNF, PCCNF (carboxyl functionalization)]; Table S11: Exposure scenario ranking for CS2.1: carboxylated CNF/CNC food packaging film; Table S12: Exposure scenario ranking for CS2.2: sulfated CNF/CNC food packaging coating; Table S13: Exposure scenario ranking for CS2.3: sulfated CNF/CNC food packaging additive; Table S14: Exposure scenario ranking for CS3: carboxylated CNF/CNC food additive; Figure S1: Atomic force micrographs of SCNFs ((a–d); top row), TCNFs ((a–d); middle row), and PCCNFs ((a–d); bottom row), designed, synthesized, and characterized for hazard assessment [24]. The micrographs show how differences in manufacturing conditions (concentration, time, blending) resulted in materials that varied by chemistry and morphology; Hazard Assessment: Literature Review.

## Abbreviations

The following abbreviations are used in this manuscript:

CNs	cellulose nanomaterials
MFC	microfibrillated cellulose
CNFs	cellulose nanofibers or nanofibrils
SbD Toolbox	Safer-by-Design Toolbox
NM	nanomaterial
Nano LCRA	nanomaterial life-cycle risk assessment
CSs	case studies
TEMPO	2,2,6,6-tetramethylpiperidin-1-oxyl radical
TCNF	TEMPO-oxidized carboxylated CNF
PC	periodate-chlorite
PCCNF	periodate-chlorite oxidized carboxylated CNF
SCNF	sulfated CNF
A549 cells	human epithelial lung cells
JAWSII	dendritic cells
FBS	Fetal Bovine Serum
cAMEM	Alpha minimum essential medium
VAG	Vilnius aerosol generator
HDFa	human dermal fibroblasts
HEKa	human epidermal keratinocytes
LDH	lactate dehydrogenase
IL	interleukin
NOAEL	No Observed Adverse Effect Level
LOAEL	Lowest Observed Adverse Effect Level
PPE	Personal Protective Equipment
OEA	occupational exposure assessments
NIOSH	National Institute for Occupational Safety and Health
CNCs	cellulose nanocrystals
NR/CC	natural rubber latex/cellulose (nano)crystals
PLA	polylactic acid
PHB	poly(3-hydroxybutyrate)
SAP	superabsorbent polymer
OECD	Organisation for Economic Co-operation and Development
THP-1 cells	monocyte-derived macrophages
BALF	bronchoalveolar lavage fluid
SAA3	Serum Amyloid A3
MNPCEs	micronucleated bone marrow polychromatic erythrocytes
S-CNC	sulfated CNC
LC_50_	Lethal Concentration 50%
EC_50_	Half Maximal Effective Concentration
LD_50_	Lethal Dose 50%
