# Supplementary material for: Life-Cycle Risk Assessment of Second-Generation Cellulose Nanomaterials
Source: Nanomaterials (Basel). 2025 Feb 4;15(3):238. doi: 10.3390/nano15030238 (PMC11819754; doi:10.3390/nano15030238)
Supplement: Supplementary file 1 [file nanomaterials-15-00238-s001.zip › nanomaterials-3412952-supplementary.pdf]

nanomaterials

*Supplementary Materials*

# Life-Cycle Risk Assessment of Second-Generation Cellulose Nanomaterials

**James D. Ede [1,*], Amanda K. Charlton-Sevcik [2], Julia Griffin [1], Padmapriya Srinivasan [1,3], Yueyang Zhang [1,4], Christie M. Sayes [2], You-Lo Hsieh [5], Nicole Stark [6] and Jo Anne Shatkin [1,*]**

[1] Vireo Advisors, LLC, Boston, MA 02205, USA
[2] Department of Environmental Science, Baylor University, Waco, TX 76798, USA
[3] Department of Chemistry, University of British Columbia, Vancouver, BC V6T 1Z4, Canada
[4] Department of Biological Sciences, University of Alberta, Edmonton, AB T6G 2E9, Canada
[5] Biological and Agricultural Engineering, Chemical Engineering, University of California at Davis, Davis, CA 95616, USA
[6] Forest Products Laboratory (FPL), USDA Forest Service, Madison, WI 53726, USA
* Correspondence: jede@vireoadvisors.com (J.D.E.); jashatkin@vireoadvisors.com (J.A.S.)

## 1. Assumptions Adopted During Case Study Development

**Table S1.** Assumptions adopted for CS1: water filtration membrane [TCNF, PCCNF (carboxyl functionalization)].

| | |
|---|---|
| **Scenario** | • Manufacture and use of a CNF membrane for water filtration<br>• TCNF and PCCNF (carboxylated) chemistries |
| **All Life-Cycle Stages** | • In evaluating potential exposures across product life, assumes no PPE is used<br>• Nano LCRA evaluates the safety of the CN; the safety of other ingredients and materials are not considered |
| **Raw Material** | • Harvesting, chipping/shredding, pulping of hardwood or softwood |
| **Product Manufacturing** | • Follows conventional carboxylation steps (TEMPO or P/C oxidized) for CN<br>• CN is produced in an enclosed batch reactor<br>• CN produced as a 2% suspension/gel<br>• Manufacture includes a drying step to produce a redispersible powder<br>• CN powder is handled, bagged and shipped<br>• Incidental release of CN powder possible from manufacturing or drying equipment<br>• Accidental spill of CN during manufacture possible<br>• CN manufacture takes place indoors with limited environmental release |
| **Product Application** | • Shipping/transportation of CN to product application facility<br>• Redispersion of CN powder required as part of product application; powder exposures possible during handing<br>• Incidental release and accidental spill of CN during product application is possible<br>• Product application takes place indoors with limited environmental release<br>• CN formulation is prepared at < 2% wt. aqueous solution<br>• CN formulation is cast to form a membrane<br>• Membrane is dried prior to handling; membrane is > 10% wt. CN<br>• CN membrane may be treated to improve absorption capability |
| **Product Use** | • Membrane handling during installation<br>• Membrane has a pre-defined commercial lifetime<br>• CN membrane is primarily used for water filtration intended for consumption<br>• CN membrane may be used for large-scale water filtration (e.g., occupational exposures) or for direct use by consumers |
| **Re-use/** | • Shipping/transportation of CN products to re-use/recycling/composting facility |

| | |
|---|---|
| **Recycling** | • Assumes direct contact with the CN products during during re-use/recycling/composting process is limited, and that these processes takes place in an open system<br>• Composting may be done at consumer or industrial scale<br>• Re-use is only expected to happen once in lifetime of CN filter membrane<br>• Bioconversion processes may include chemical breakdown with biological processes or agents |
| **Disposal** | • Transportation of discarded CN membrane to end-of-life location<br>• Primary disposal facilities include (1) long-term municipal solid waste landfill or (2) incineration in waste-to-energy facility<br>• Assumes some CN products is discarded in environment (e.g. litter) |

**Table S2.** Assumptions adopted for CS2.1: food packaging film [TCNF, PCCNF (carboxyl functionalization)].

| | |
|---|---|
| **Scenario** | • Manufacture and use of a CNF food packaging film<br>• TCNF and PCCNF (carboxylated) chemistries |
| **All Life-Cycle Stages** | • In evaluating potential exposures across product life, assumes no PPE is used<br>• Nano LCRA evaluates the safety of the CN; the safety of other ingredients and materials are not considered |
| **Raw Material** | • Harvesting, chipping/shredding, pulping of hardwood or softwood |
| **Product Manufacturing** | • Follows conventional carboxylation steps (TEMPO or P/C oxidized) for CN<br>• CN is produced in an enclosed batch reactor<br>• CN produced as a 2% suspension/gel<br>• Manufacture includes a drying step to produce a redispersible powder<br>• CN powder is handled, bagged and shipped<br>• Incidental release of CN powder possible from manufacturing or drying equipment<br>• Accidental spill of CN during manufacture possible<br>• CN manufacture takes place indoors with limited environmental release |
| **Product Application** | • Shipping/transportation of CN to product application facility<br>• Redispersion of CN powder required as part of product application; powder exposures possible during handing<br>• Incidental release and accidental spill of CN during product application is possible<br>• Product application takes place indoors with limited environmental release<br>• CN formulation is prepared at < 2% wt. aqueous solution<br>• Assume CN is cast at < 2% wt. to form a food packaging film<br>• CN film is dried prior to handling and may be up to 100% wt. CN<br>• Optional finishing steps of CN film include hot pressing, embossing, and folding, as well as application of potential coatings |
| **Product Use** | • Single-use food packaging film in direct contact with food<br>• Misuse/degradation of the food packaging film is possible |
| **Re-use/ Recycling** | • Shipping/transportation of CN products to re-use/recycling/composting facility<br>• Assumes direct contact with the CN products during during re-use/recycling/composting process is limited, and that these processes takes place in an open system<br>• Recycling activities may include physical (e.g. tearing) and/or chemical (e.g. pulping) breakdown<br>• Composting may be done at consumer or industrial scale<br>• Bioconversion processes may include chemical breakdown with biological processes or agents |
| **Disposal** | • Transportation of discarded CN packaging film to end-of-life location<br>• Primary disposal facilities include (1) long-term municipal solid waste landfill or (2) incineration in waste-to-energy facility<br>• Assumes some CN packaging films is discarded in environment (e.g. litter) |

**Table S3.** Assumptions adopted for CS2.2: food packaging coating (SCNF).

| | |
|---|---|
| **Scenario** | • Manufacture and use of a CNF barrier coating applied to food contact packaging<br>• SCNF (sulfated) chemistry |
| **All Life-Cycle Stages** | • In evaluating potential exposures across product life, assumes no PPE is used<br>• Nano LCRA evaluates the safety of the CN; the safety of other ingredients and materials are not considered |
| **Raw Material** | • Harvesting, chipping/shredding, pulping of hardwood or softwood |
| **Product Manufacturing** | • Follows conventional sulfation steps for CN<br>• CN is produced in an enclosed batch reactor<br>• CN produced as a 2% suspension/gel<br>• Manufacture includes a drying step to produce a redispersible powder<br>• CN powder is handled, bagged and shipped<br>• Incidental release of CN powder possible from manufacturing or drying equipment<br>• Accidental spill of CN during manufacture possible<br>• CN manufacture takes place indoors with limited environmental release |
| **Product Application** | • Shipping/transportation of CN to product application facility<br>• Redispersion of CN powder required as part of product application; powder exposures possible during handing<br>• Incidental release and accidental spill of CN during product application is possible<br>• Product application takes place indoors with limited environmental release<br>• CN coating formulation is prepared at < 2% wt. aqueous solution<br>• CN coating is applied to conventional food contact paper/board using spray coating.<br>• Coating is left to dry/cure and may be up to 100% CN<br>• Optional finishing step of the CN coating include hot pressing and folding |
| **Product Use** | • Single-use food contact paper/ board coated with CN in direct contact with food<br>• Misuse/degradation of the CN coated food contact paper/board is possible |
| **Re-use/ Recycling** | • Shipping/transportation of CN products to re-use/recycling/composting facility<br>• Assumes direct contact with the CN products during during re-use/recycling/composting process is limited, and that these processes takes place in an open system<br>• Recycling activities may include physical (e.g. tearing) and/or chemical (e.g. pulping) breakdown<br>• Composting may be done at consumer or industrial scale<br>• Bioconversion processes may include chemical breakdown with biological processes or agents |
| **Disposal** | • Transportation of discarded CN-coated packaging to end-of-life location<br>• Primary disposal facilities include (1) long-term municipal solid waste landfill or (2) incineration in waste-to-energy facility<br>• Assumes some CN-coated packaging is discarded in environment (e.g. litter) |

**Table S4.** Assumptions adopted for CS2.3: food packaging additive (SCNF).

| | |
|---|---|
| **Scenario** | • Manufacture and use of CNF as a food packaging additive<br>• SCNF (sulfated) chemistry |
| **All Life-Cycle Stages** | • In evaluating potential exposures across product life, assumes no PPE is used<br>• Nano LCRA evaluates the safety of the CN; the safety of other ingredients and materials are not considered |
| **Raw Material** | • Harvesting, chipping/shredding, pulping of hardwood or softwood |
| **Product Manufacturing** | • Follows conventional sulfation steps for CN<br>• CN is produced in an enclosed batch reactor<br>• CN produced as a 2% suspension/gel<br>• Manufacture includes a drying step to produce a redispersible powder<br>• CN powder is handled, bagged and shipped<br>• Incidental release of CN powder possible from manufacturing or drying equipment<br>• Accidental spill of CN during manufacture possible<br>• Manufacture takes place indoors with limited environmental release |
| **Product Application** | • Shipping/transportation of CN to product application facility<br>• Redispersion of CN powder required as part of product application; powder exposures possible during handing<br>• Incidental release and accidental spill of CN during product application is possible<br>• Product application takes place indoors with limited environmental release<br>CNs are mixed into traditional ingredients used to make food contact paper/board (e.g., pulp)<br>• Assumes traditional papermaking methods are used (e.g., screening, pressing and drying steps)<br>• Optional finishing steps for CN paper/board may include hot pressing, embossing or folding |
| **Product Use** | • Single-use food contact paper/ board containing up to 5% wt. CN in direct contact with food<br>• Misuse/degradation of the CN containing food contact paper/board is possible |
| **Re-use/ Recycling** | • Shipping/transportation of CN products to re-use/recycling/composting facility<br>• Assumes direct contact with the CN products during during re-use/recycling/composting process is limited, and that these processes takes place in an open system<br>• Recycling activities may include physical (e.g. tearing) and/or chemical (e.g. pulping) breakdown<br>• Composting may be done at consumer or industrial scale<br>• Bioconversion processes may include chemical breakdown with biological processes or agents |
| **Disposal** | • Transportation of discarded CN-containing packaging to end-of-life location<br>• Primary disposal facilities include (1) long-term municipal solid waste landfill or (2) incineration in waste-to-energy facility<br>• Assumes some CN-containing packaging is discarded in environment (e.g. litter) |

**Table S5.** Assumptions adopted for CS3: food additive [TCNF, PCCNF (carboxyl functionalization)].

| | |
|---|---|
| **Scenario** | • Manufacture and use of CNF as a food additive<br>• TCNF and PCCNF (Carboxylated) chemistries |
| **All Life-Cycle Stages** | • In evaluating potential exposures across product life, assumes no PPE is used<br>• Nano LCRA evaluates the safety of the CN; the safety of other ingredients and materials are not considered |
| **Raw Material** | • Harvesting, chipping/shredding, pulping of hardwood or softwood |
| **Product Manufacturing** | • Follows conventional carboxylation steps (TEMPO or P/C oxidized) for CN<br>• CN is produced in an enclosed batch reactor<br>• CN produced as a 2% suspension/gel<br>• Manufacture includes a drying step to produce a redispersible powder<br>• CN powder is handled, bagged and shipped<br>• Incidental release of CN powder possible from manufacturing or drying equipment<br>• Accidental spill of CN during manufacture possible<br>• Manufacture takes place indoors with limited environmental release |
| **Product Application** | • Shipping/transportation of CN to product application facility<br>• Redispersion of CN powder required as part of product application; powder exposures possible during handing<br>• Incidental release and accidental spill of CN during product application is possible<br>• Product application takes place indoors with limited environmental release<br>• CNs are incorporated into food for a variety of technical effects (e.g., stabilizer, emulsifier, thickener, calorie reducer)<br>• CNs added in up to 5% wt. using high shear mixing or similar processes in an open system |
| **Product Use** | • Assumes CN-containing foods are handled and consumed typically by consumers |
| **Re-use/ Recycling** | • Shipping/transportation of CN products to re-use/recycling/composting facility<br>• Assumes direct contact with the CN products during during re-use/recycling/composting process is limited, and that these processes takes place in an open system<br>• Assumes food waste is primarily re-used for animal feed, or composting at consumer or industrial scale<br>• Bioconversion processes may include chemical breakdown with biological processes or agents |
| **Disposal** | • Transportation of discarded CN-containing food waste to end-of-life location<br>• Primary disposal facilities include (1) long-term municipal solid waste landfill or (2) incineration in waste-to-energy facility<br>• Assumes some CN-containing food is discarded in environment (e.g. litter) |

## 2. Exposure Scenario Development

**Table S6.** Exposure scenarios for CS1: water filtration membrane [TCNF, PCCNF (carboxyl functionalization)].

| Life-Cycle Stage | LC Code | LCS # | Scenario # | Scenario | Receptor | Exposure Route |
|---|---|---|---|---|---|---|
| Raw Material | RM | 1 | 1 | Harvesting, chipping/shredding, pulping softwood or hardwood | occupational | inhalation |
| Product Manufacturing | PM | 2 | 1 | Cleaning out synthesis equipment | occupational | inhalation |
| Product Manufacturing | PM | 2 | 2 | Cleaning out synthesis equipment | occupational | dermal/eye |
| Product Manufacturing | PM | 2 | 3 | Cleaning out synthesis equipment | environmental | direct |
| Product Manufacturing | PM | 2 | 4 | Incidental release of CN from synthesis equipment | occupational | inhalation |
| Product Manufacturing | PM | 2 | 5 | Incidental release of CN from synthesis equipment | occupational | dermal/eye |
| Product Manufacturing | PM | 2 | 6 | Incidental release of CN from synthesis equipment | environmental | direct |
| Product Manufacturing | PM | 2 | 7 | Accidental spill of CN from synthesis equipment | occupational | inhalation |
| Product Manufacturing | PM | 2 | 8 | Accidental spill of CN from synthesis equipment | occupational | dermal/eye |
| Product Manufacturing | PM | 2 | 9 | Accidental spill of CN from synthesis equipment | environmental | direct |
| Product Manufacturing | PM | 2 | 10 | Dried formulation extraction and handling (for powder NC ingredients) | occupational | inhalation |
| Product Manufacturing | PM | 2 | 11 | Dried formulation extraction and handling (for powder NC ingredients) | occupational | dermal/eye |
| Product Application | PA | 3 | 1 | Transfer CN from synthesis to application facility (e.g. handling, packaging) | occupational | inhalation |
| Product Application | PA | 3 | 2 | Transfer CN from synthesis to application facility (e.g. handling, packaging) | occupational | dermal/eye |
| Product Application | PA | 3 | 3 | Deposition and formulation equipment cleanout | occupational | inhalation |
| Product Application | PA | 3 | 4 | Deposition and formulation equipment cleanout | occupational | dermal/eye |
| Product Application | PA | 3 | 5 | Deposition and formulation equipment cleanout | environmental | direct |
| Product Application | PA | 3 | 6 | CN rehydration (if powder NC ingredient) | occupational | inhalation |
| Product Application | PA | 3 | 7 | CN rehydration (if powder NC ingredient) | occupational | dermal/eye |
| Product Application | PA | 3 | 8 | CN formulation preparation (e.g. mixing with other ingredients, homogenizing, other) | occupational | inhalation |
| Product Application | PA | 3 | 9 | CN formulation preparation (e.g. mixing with other ingredients, homogenizing, other) | occupational | dermal/eye |
| Product Application | PA | 3 | 10 | CN casting to form membrane | occupational | inhalation |
| Product Application | PA | 3 | 11 | CN casting to form membrane | occupational | dermal/eye |
| Product Application | PA | 3 | 12 | Incidental release of CN from deposition equipment | occupational | inhalation |
| Product Application | PA | 3 | 13 | Incidental release of CN from deposition equipment | occupational | dermal/eye |
| Product Application | PA | 3 | 14 | Incidental release of CN from deposition equipment | environmental | direct |
| Product Application | PA | 3 | 15 | Accidental spill of CN from deposition equipment | occupational | inhalation |
| Product Application | PA | 3 | 16 | Accidental spill of CN from deposition equipment | occupational | dermal/eye |

| | | | | | | |
|---|---|---|---|---|---|---|
| Product Application | PA | 3 | 17 | Accidental spill of CN from deposition equipment | environmental | direct |
| Product Application | PA | 3 | 18 | CN drying (membrane formation) | occupational | inhalation |
| Product Application | PA | 3 | 19 | CN drying (membrane formation) | occupational | dermal/eye |
| Product Application | PA | 3 | 20 | Surface treatment of CN membrane (functionalization, other) | occupational | inhalation |
| Product Application | PA | 3 | 21 | Surface treatment of CN membrane (functionalization, other) | occupational | dermal/eye |
| Product Use | PU | 4 | 1 | Membrane handling, installation, and removal | occupational | inhalation |
| Product Use | PU | 4 | 2 | Membrane handling, installation, and removal | occupational | dermal/eye |
| Product Use | PU | 4 | 3 | Membrane handling, installation, and removal | consumer | dermal/eye |
| Product Use | PU | 4 | 4 | Use of membrane to filter drinking water | occupational | ingestion |
| Product Use | PU | 4 | 5 | Use of membrane to filter drinking water | consumer | ingestion |
| Product Use | PU | 4 | 6 | Use of membrane to filter drinking water | environmental | direct |
| Re-use/Recycling | RR | 5 | 1 | Collection and transport of used membrane to re-use or composting facility | occupational | inhalation |
| Re-use/Recycling | RR | 5 | 2 | Collection and transport of used membrane to re-use or composting facility | occupational | dermal/eye |
| Re-use/Recycling | RR | 5 | 5 | Composting used membrane | consumer | inhalation |
| Re-use/Recycling | RR | 5 | 6 | Composting used membrane | consumer | dermal/eye |
| Re-use/Recycling | RR | 5 | 3 | Composting used membrane | occupational | inhalation |
| Re-use/Recycling | RR | 5 | 4 | Composting used membrane | occupational | dermal/eye |
| Re-use/Recycling | RR | 5 | 7 | Composting used membrane | environmental | direct |
| Re-use/Recycling | RR | 5 | 8 | Bioconversion (anaerobic digestion) | occupational | inhalation |
| Re-use/Recycling | RR | 5 | 9 | Bioconversion (anaerobic digestion) | occupational | dermal/eye |
| Re-use/Recycling | RR | 5 | 10 | Bioconversion (anaerobic digestion) | environmental | direct |
| Re-use/Recycling | RR | 5 | 11 | Continued use (degraded membrane) | consumer | dermal/eye |
| Re-use/Recycling | RR | 5 | 12 | Continued use (degraded membrane) | environmental | direct |
| Disposal | D | 6 | 1 | Collection and transport to final end-of-life location | occupational | dermal/eye |
| Disposal | D | 6 | 2 | Collection and transport to final end-of-life location | environmental | direct |
| Disposal | D | 6 | 3 | Incineration or heat recovery | occupational | inhalation |
| Disposal | D | 6 | 4 | Incineration or heat recovery | environmental | direct |
| Disposal | D | 6 | 5 | Long-term MSW landfill storage | environmental | direct |
| Disposal | D | 6 | 6 | Membrane discarded in uncontrolled environment | environmental | direct |

**Legend:** LC Code, life-cycle code; LCS #, life-cycle stage number; RM, raw material; PM, product manufacturing; PA, product application; PU, product use; RR, re-use/recycling; D, disposal; CN, cellulose nanomaterial; TCNFs, TEMPO-oxidized cellulose nanofibers; PCCNFs, periodate-chlorite oxidized cellulose nanofibers.

**Table S7.** Exposure scenarios for CS2.1: food packaging film [TCNF, PCCNF (carboxyl functionalization)].

| Life-Cycle Stage | LC Code | LCS # | Scenario # | Scenario | Receptor | Exposure Route |
|---|---|---|---|---|---|---|
| Raw Material | RM | 1 | 1 | Harvesting, chipping/shredding, pulping softwood or hardwood | occupational | inhalation |
| Product Manufacturing | PM | 2 | 1 | Cleaning out synthesis equipment | occupational | inhalation |
| Product Manufacturing | PM | 2 | 2 | Cleaning out synthesis equipment | occupational | dermal/eye |
| Product Manufacturing | PM | 2 | 3 | Cleaning out synthesis equipment | environmental | direct |
| Product Manufacturing | PM | 2 | 4 | Incidental release of CN from synthesis equipment | occupational | inhalation |
| Product Manufacturing | PM | 2 | 5 | Incidental release of CN from synthesis equipment | occupational | dermal/eye |
| Product Manufacturing | PM | 2 | 6 | Incidental release of CN from synthesis equipment | environmental | direct |
| Product Manufacturing | PM | 2 | 7 | Accidental spill of CN from synthesis equipment | occupational | inhalation |
| Product Manufacturing | PM | 2 | 8 | Accidental spill of CN from synthesis equipment | occupational | dermal/eye |
| Product Manufacturing | PM | 2 | 9 | Accidental spill of CN from synthesis equipment | environmental | direct |
| Product Manufacturing | PM | 2 | 10 | Dried formulation extraction and handling (for powder CN ingredients) | occupational | inhalation |
| Product Manufacturing | PM | 2 | 11 | Dried formulation extraction and handling (for powder CN ingredients) | occupational | dermal/eye |
| Product Application | PA | 3 | 1 | Transfer CN from synthesis to application facility (e.g. handling, packaging) | occupational | inhalation |
| Product Application | PA | 3 | 2 | Transfer CN from synthesis to application facility (e.g. handling, packaging) | occupational | dermal/eye |
| Product Application | PA | 3 | 3 | Deposition and formulation equipment cleanout | occupational | inhalation |
| Product Application | PA | 3 | 4 | Deposition and formulation equipment cleanout | occupational | dermal/eye |
| Product Application | PA | 3 | 5 | Deposition and formulation equipment cleanout | environmental | direct |
| Product Application | PA | 3 | 6 | CN rehydration (if powder CN ingredient) | occupational | inhalation |
| Product Application | PA | 3 | 7 | CN rehydration (if powder CN ingredient) | occupational | dermal/eye |
| Product Application | PA | 3 | 8 | CN film formulation preparation (e.g. mixing with other ingredients, homogenixing) | occupational | inhalation |
| Product Application | PA | 3 | 9 | CN film formulation preparation (e.g. mixing with other ingredients, homogenixing) | occupational | dermal/eye |
| Product Application | PA | 3 | 10 | CN casting to form film | occupational | inhalation |
| Product Application | PA | 3 | 11 | CN casting to form film | occupational | dermal/eye |
| Product Application | PA | 3 | 12 | Incidental release of CN from deposition equipment | occupational | inhalation |
| Product Application | PA | 3 | 13 | Incidental release of CN from deposition equipment | occupational | dermal/eye |
| Product Application | PA | 3 | 14 | Incidental release of CN from deposition equipment | environmental | direct |
| Product Application | PA | 3 | 15 | Accidental spill of CN from deposition equipment | occupational | inhalation |
| Product Application | PA | 3 | 16 | Accidental spill of CN from deposition equipment | occupational | dermal/eye |
| Product Application | PA | 3 | 17 | Accidental spill of CN from deposition equipment | environmental | direct |
| Product Application | PA | 3 | 18 | CN drying (film formation) | occupational | inhalation |
| Product Application | PA | 3 | 19 | CN drying (film formation) | occupational | dermal/eye |

| | | | | | | |
|---|---|---|---|---|---|---|
| Product Application | PA | 3 | 20 | Surface treatment of CN film (e.g., hot press, wax coating, other) | occupational | inhalation |
| Product Application | PA | 3 | 21 | Surface treatment of CN film (e.g., hot press, wax coating, other) | occupational | dermal/eye |
| Product Application | PA | 3 | 22 | Physical treatment of CN film (e.g., forming, bending, other) | occupational | inhalation |
| Product Application | PA | 3 | 23 | Physical treatment of CN film (e.g., forming, bending, other) | occupational | dermal/eye |
| Product Use | PU | 4 | 1 | Food packaging use (release, migration to food) | consumer | ingestion |
| Product Use | PU | 4 | 2 | Food packaging handling/interaction (release) | consumer | inhalation |
| Product Use | PU | 4 | 3 | Food packaging handling/interaction (release) | consumer | dermal/eye |
| Product Use | PU | 4 | 4 | Misuse: Degraded food packaging | consumer | inhalation |
| Product Use | PU | 4 | 5 | Misuse: Degraded food packaging | consumer | dermal/eye |
| Product Use | PU | 4 | 6 | Misuse: Degraded food packaging | consumer | ingestion |
| Product Use | PU | 4 | 7 | Misuse: Degraded food packaging | environmental | direct |
| Re-use/Recycling | RR | 5 | 1 | Collection and transport of used food packaging to recycling, re-use or compost facility | occupational | inhalation |
| Re-use/Recycling | RR | 5 | 2 | Collection and transport of used food packaging to recycling, re-use or compost facility | occupational | dermal/eye |
| Re-use/Recycling | RR | 5 | 3 | Recycling activities: Physical breakdown of used food packaging (e.g. tearing) | occupational | inhalation |
| Re-use/Recycling | RR | 5 | 4 | Recycling activities: Physical breakdown of used food packaging (e.g. tearing) | occupational | dermal/eye |
| Re-use/Recycling | RR | 5 | 5 | Recycling activities: Chemical breakdown of used food packaging (e.g. pulping) | occupational | inhalation |
| Re-use/Recycling | RR | 5 | 6 | Recycling activities: Chemical breakdown of used food packaging (e.g. pulping) | occupational | dermal/eye |
| Re-use/Recycling | RR | 5 | 7 | Composting used food packaging | consumer | inhalation |
| Re-use/Recycling | RR | 5 | 8 | Composting used food packaging | consumer | dermal/eye |
| Re-use/Recycling | RR | 5 | 9 | Composting used food packaging | occupational | inhalation |
| Re-use/Recycling | RR | 5 | 10 | Composting used food packaging | occupational | dermal/eye |
| Re-use/Recycling | RR | 5 | 11 | Composting used food packaging | environmental | direct |
| Re-use/Recycling | RR | 5 | 12 | Bioconversion (anaerobic digestion) | occupational | inhalation |
| Re-use/Recycling | RR | 5 | 13 | Bioconversion (anaerobic digestion) | occupational | dermal/eye |
| Re-use/Recycling | RR | 5 | 14 | Bioconversion (anaerobic digestion) | environmental | direct |
| Disposal | D | 6 | 1 | Collection and transport to final end-of-life location | occupational | dermal/eye |
| Disposal | D | 6 | 2 | Collection and transport to final end-of-life location | environmental | direct |
| Disposal | D | 6 | 3 | Incineration or heat recovery | occupational | inhalation |
| Disposal | D | 6 | 4 | Incineration or heat recovery | environmental | direct |
| Disposal | D | 6 | 5 | Long-term MSW landfill storage | environmental | direct |
| Disposal | D | 6 | 6 | Food packaging discarded in uncontrolled environment | environmental | direct |

**Legend:** LC Code, life-cycle code; LCS #, life-cycle stage number; RM, raw material; PM, product manufacturing; PA, product application; PU, product use; RR, re-use/recycling; D, disposal; CN, cellulose nanomaterial; TCNFs, TEMPO-oxidized cellulose nanofibers; PCCNFs, periodate-chlorite oxidized cellulose nanofibers.

**Table S8.** Exposure scenarios for CS2.2: food packaging coating (SCNF).

| Life-Cycle Stage | LC Code | LCS # | Scenario # | Scenario | Receptor | Exposure Route |
|---|---|---|---|---|---|---|
| Raw Material | RM | 1 | 1 | Harvesting, chipping/shredding, pulping softwood or hardwood | occupational | inhalation |
| Product Manufacturing | PM | 2 | 1 | Cleaning out synthesis equipment | occupational | inhalation |
| Product Manufacturing | PM | 2 | 2 | Cleaning out synthesis equipment | occupational | dermal/eye |
| Product Manufacturing | PM | 2 | 3 | Cleaning out synthesis equipment | environmental | direct |
| Product Manufacturing | PM | 2 | 4 | Incidental spill of CN from synthesis equipment | occupational | inhalation |
| Product Manufacturing | PM | 2 | 5 | Incidental spill of CN from synthesis equipment | occupational | dermal/eye |
| Product Manufacturing | PM | 2 | 6 | Incidental spill of CN from synthesis equipment | environmental | direct |
| Product Manufacturing | PM | 2 | 7 | Accidental spill of CN from synthesis equipment | occupational | inhalation |
| Product Manufacturing | PM | 2 | 8 | Accidental spill of CN from synthesis equipment | occupational | dermal/eye |
| Product Manufacturing | PM | 2 | 9 | Accidental spill of CN from synthesis equipment | environmental | direct |
| Product Manufacturing | PM | 2 | 10 | Dried formulation extraction and handling (for powder CN ingredients) | occupational | inhalation |
| Product Manufacturing | PM | 2 | 11 | Dried formulation extraction and handling (for powder CN ingredients) | occupational | dermal/eye |
| Product Application | PA | 3 | 1 | Transfer CN from synthesis to application facility (e.g. handling, packaging) | occupational | inhalation |
| Product Application | PA | 3 | 2 | Transfer CN from synthesis to application facility (e.g. handling, packaging) | occupational | dermal/eye |
| Product Application | PA | 3 | 3 | Deposition (and formulation prep) equipment cleanout | occupational | inhalation |
| Product Application | PA | 3 | 4 | Deposition (and formulation prep) equipment cleanout | occupational | dermal/eye |
| Product Application | PA | 3 | 5 | Deposition (and formulation prep) equipment cleanout | environmental | direct |
| Product Application | PA | 3 | 6 | CN rehydration (if powder NC ingredient) | occupational | inhalation |
| Product Application | PA | 3 | 7 | CN rehydration (if powder NC ingredient) | occupational | dermal/eye |
| Product Application | PA | 3 | 8 | CN coating formulation preparation (e.g. mixing with other ingredients, homogenixing) | occupational | inhalation |
| Product Application | PA | 3 | 9 | CN coating formulation preparation (e.g. mixing with other ingredients, homogenixing) | occupational | dermal/eye |
| Product Application | PA | 3 | 10 | CN formulation applied via spraying coating on paper food contact material | occupational | inhalation |
| Product Application | PA | 3 | 11 | CN formulation applied via spraying coating on paper food contact material | occupational | dermal/eye |
| Product Application | PA | 3 | 12 | Incidental release of CN from deposition equipment | occupational | inhalation |
| Product Application | PA | 3 | 13 | Incidental release of CN from deposition equipment | occupational | dermal/eye |
| Product Application | PA | 3 | 14 | Incidental release of CN from deposition equipment | environmental | direct |
| Product Application | PA | 3 | 15 | Accidental spill of CN from deposition equipment | occupational | inhalation |
| Product Application | PA | 3 | 16 | Accidental spill of CN from deposition equipment | occupational | dermal/eye |
| Product Application | PA | 3 | 17 | Accidental spill of CN from deposition equipment | environmental | direct |
| Product Application | PA | 3 | 18 | CN coating dried on paper substrate (coating formation) | occupational | inhalation |

| | | | | | | |
|---|---|---|---|---|---|---|
| Product Application | PA | 3 | 19 | CN coating dried on paper substrate (coating formation) | occupational | dermal/eye |
| Product Application | PA | 3 | 20 | Surface treatment of CN coating (hot press, other) | occupational | inhalation |
| Product Application | PA | 3 | 21 | Surface treatment of CN coating (hot press, other) | occupational | dermal/eye |
| Product Application | PA | 3 | 22 | Physical treatment of CN coating (e.g. forming, bending, other) | occupational | inhalation |
| Product Application | PA | 3 | 23 | Physical treatment of CN coating (e.g. forming, bending, other) | occupational | dermal/eye |
| Product Use | PU | 4 | 1 | Food packaging use (release, migration) | consumer | ingestion |
| Product Use | PU | 4 | 2 | Food packaging handling/interaction (release) | consumer | inhalation |
| Product Use | PU | 4 | 3 | Food packaging handling/interaction (release) | consumer | dermal/eye |
| Product Use | PU | 4 | 4 | Misuse: Degraded food packaging | consumer | inhalation |
| Product Use | PU | 4 | 5 | Misuse: Degraded food packaging | consumer | dermal/eye |
| Product Use | PU | 4 | 6 | Misuse: Degraded food packaging | consumer | ingestion |
| Product Use | PU | 4 | 7 | Misuse: Degraded food packaging | environmental | direct |
| Re-use/Recycling | RR | 5 | 1 | Collection and transport of used food packaging to recycling, re-use or compost facility | occupational | inhalation |
| Re-use/Recycling | RR | 5 | 2 | Collection and transport of used food packaging to recycling, re-use or compost facility | occupational | dermal/eye |
| Re-use/Recycling | RR | 5 | 3 | Recycling activities: Physical breakdown of used food packaging (e.g. tearing) | occupational | inhalation |
| Re-use/Recycling | RR | 5 | 4 | Recycling activities: Physical breakdown of used food packaging (e.g. tearing) | occupational | dermal/eye |
| Re-use/Recycling | RR | 5 | 5 | Recycling activities: Chemical breakdown of used food packaging (e.g. pulping) | occupational | inhalation |
| Re-use/Recycling | RR | 5 | 6 | Recycling activities: Chemical breakdown of used food packaging (e.g. pulping) | occupational | dermal/eye |
| Re-use/Recycling | RR | 5 | 7 | Composting used food packaging | consumer | inhalation |
| Re-use/Recycling | RR | 5 | 8 | Composting used food packaging | consumer | dermal/eye |
| Re-use/Recycling | RR | 5 | 9 | Composting used food packaging | occupational | inhalation |
| Re-use/Recycling | RR | 5 | 10 | Composting used food packaging | occupational | dermal/eye |
| Re-use/Recycling | RR | 5 | 11 | Composting used food packaging | environmental | direct |
| Re-use/Recycling | RR | 5 | 12 | Bio-conversion (anaerobic digestion) | occupational | inhalation |
| Re-use/Recycling | RR | 5 | 13 | Bio-conversion (anaerobic digestion) | occupational | dermal/eye |
| Re-use/Recycling | RR | 5 | 14 | Bio-conversion (anaerobic digestion) | environmental | direct |
| Disposal | D | 6 | 1 | Collection and transport to final end-of-life location | occupational | dermal/eye |
| Disposal | D | 6 | 2 | Collection and transport to final end-of-life location | environmental | direct |
| Disposal | D | 6 | 3 | Incineration or heat recovery | occupational | inhalation |
| Disposal | D | 6 | 4 | Incineration or heat recovery | environmental | direct |
| Disposal | D | 6 | 5 | Long-term MSW landfill storage | environmental | direct |
| Disposal | D | 6 | 6 | Food packaging discarded in uncontrolled environment | environmental | direct |

**Legend:** LC Code, life-cycle code; LCS #, life-cycle stage number; RM, raw material; PM, product manufacturing; PA, product application; PU, product use; RR, re-use/recycling; D, disposal; CN, cellulose nanomaterial; SCNF, sulfated cellulose nanofibers.

**Table S9.** Exposure scenarios for CS2.3: food packaging additive (SCNF).

| Life-Cycle Stage | LC Code | LCS # | Scenario # | Scenario | Receptor | Exposure Route |
|---|---|---|---|---|---|---|
| Raw Material | RM | 1 | 1 | Harvesting, chipping/shredding, pulping softwood or hardwood | occupational | inhalation |
| Product Manufacturing | PM | 2 | 1 | Cleaning out synthesis equipment | occupational | inhalation |
| Product Manufacturing | PM | 2 | 2 | Cleaning out synthesis equipment | occupational | dermal/eye |
| Product Manufacturing | PM | 2 | 3 | Cleaning out synthesis equipment | environmental | direct |
| Product Manufacturing | PM | 2 | 4 | Incidental release of CN from synthesis equipment | occupational | inhalation |
| Product Manufacturing | PM | 2 | 5 | Incidental release of CN from synthesis equipment | occupational | dermal/eye |
| Product Manufacturing | PM | 2 | 6 | Incidental release of CN from synthesis equipment | environmental | direct |
| Product Manufacturing | PM | 2 | 7 | Accidental spill of CN from synthesis equipment | occupational | inhalation |
| Product Manufacturing | PM | 2 | 8 | Accidental spill of CN from synthesis equipment | occupational | dermal/eye |
| Product Manufacturing | PM | 2 | 9 | Accidental spill of CN from synthesis equipment | environmental | direct |
| Product Manufacturing | PM | 2 | 10 | Dried formulation extraction and handling (for powder CN ingredients) | occupational | inhalation |
| Product Manufacturing | PM | 2 | 11 | Dried formulation extraction and handling (for powder CN ingredients) | occupational | dermal/eye |
| Product Application | PA | 3 | 1 | Transfer CN from synthesis to application facility (e.g. handling, packaging) | occupational | inhalation |
| Product Application | PA | 3 | 2 | Transfer CN from synthesis to application facility (e.g. handling, packaging) | occupational | dermal/eye |
| Product Application | PA | 3 | 3 | Formulation and papermaking equipment cleanout | occupational | inhalation |
| Product Application | PA | 3 | 4 | Formulation and papermaking equipment cleanout | occupational | dermal/eye |
| Product Application | PA | 3 | 5 | Formulation and papermaking equipment cleanout | environmental | direct |
| Product Application | PA | 3 | 6 | CN rehydration (if powder CN ingredient) | occupational | inhalation |
| Product Application | PA | 3 | 7 | CN rehydration (if powder CN ingredient) | occupational | dermal/eye |
| Product Application | PA | 3 | 8 | CN paper formulation preparation (e.g. mixing with other ingredients, homogenixing) | occupational | inhalation |
| Product Application | PA | 3 | 9 | CN paper formulation preparation (e.g. mixing with other ingredients, homogenixing) | occupational | dermal/eye |
| Product Application | PA | 3 | 10 | Casting CN paper (screening, pressing) | occupational | inhalation |
| Product Application | PA | 3 | 11 | Casting CN paper (screening, pressing) | occupational | dermal/eye |
| Product Application | PA | 3 | 12 | Incidental release of CN from papermaking equipment | occupational | inhalation |
| Product Application | PA | 3 | 13 | Incidental release of CN from papermaking equipment | occupational | dermal/eye |
| Product Application | PA | 3 | 14 | Incidental release of CN from papermaking equipment | environmental | direct |
| Product Application | PA | 3 | 15 | Accidental spill of CN from papermaking equipment | occupational | inhalation |
| Product Application | PA | 3 | 16 | Accidental spill of CN from papermaking equipment | occupational | dermal/eye |
| Product Application | PA | 3 | 17 | Accidental spill of CN from papermaking equipment | environmental | direct |
| Product Application | PA | 3 | 18 | CN paper drying | occupational | inhalation |
| Product Application | PA | 3 | 19 | CN paper drying | occupational | dermal/eye |

| | | | | | | |
|---|---|---|---|---|---|---|
| Product Application | PA | 3 | 20 | Surface treatment of CN paper product (e.g., hot press, embossing, other) | occupational | inhalation |
| Product Application | PA | 3 | 21 | Surface treatment of CN paper product (e.g., hot press, embossing, other) | occupational | dermal/eye |
| Product Application | PA | 3 | 22 | Physical treatment of CN paper (e.g. forming, bending, other) | occupational | inhalation |
| Product Application | PA | 3 | 23 | Physical treatment of CN paper (e.g. forming, bending, other) | occupational | dermal/eye |
| Product Use | PU | 4 | 1 | Food packaging use (release, migration) | consumer | ingestion |
| Product Use | PU | 4 | 2 | Food packaging handling | consumer | inhalation |
| Product Use | PU | 4 | 3 | Food packaging handling | consumer | dermal/eye |
| Product Use | PU | 4 | 4 | Misuse: Degraded food packaging | consumer | inhalation |
| Product Use | PU | 4 | 5 | Misuse: Degraded food packaging | consumer | dermal/eye |
| Product Use | PU | 4 | 6 | Misuse: Degraded food packaging | consumer | ingestion |
| Product Use | PU | 4 | 7 | Misuse: Degraded food packaging | environmental | direct |
| Re-use/Recycling | RR | 5 | 1 | Collection and transport to re-use facility of used food packaging | occupational | inhalation |
| Re-use/Recycling | RR | 5 | 2 | Collection and transport to re-use facility of used food packaging | occupational | dermal/eye |
| Re-use/Recycling | RR | 5 | 3 | Recycling activities: Physical breakdown of used food packaging (e.g. tearing) | occupational | inhalation |
| Re-use/Recycling | RR | 5 | 4 | Recycling activities: Physical breakdown of used food packaging (e.g. tearing) | occupational | dermal/eye |
| Re-use/Recycling | RR | 5 | 5 | Recycling activities: Chemical breakdown of used food packaging (e.g. pulping) | occupational | inhalation |
| Re-use/Recycling | RR | 5 | 6 | Recycling activities: Chemical breakdown of used food packaging (e.g. pulping) | occupational | dermal/eye |
| Re-use/Recycling | RR | 5 | 7 | Composting used food packaging | consumer | inhalation |
| Re-use/Recycling | RR | 5 | 8 | Composting used food packaging | consumer | dermal/eye |
| Re-use/Recycling | RR | 5 | 9 | Composting used food packaging | occupational | inhalation |
| Re-use/Recycling | RR | 5 | 10 | Composting used food packaging | occupational | dermal/eye |
| Re-use/Recycling | RR | 5 | 11 | Composting used food packaging | environmental | direct |
| Re-use/Recycling | RR | 5 | 12 | Bio-conversion (anaerobic digestion) | occupational | inhalation |
| Re-use/Recycling | RR | 5 | 13 | Bio-conversion (anaerobic digestion) | occupational | dermal/eye |
| Re-use/Recycling | RR | 5 | 14 | Bio-conversion (anaerobic digestion) | environmental | direct |
| Disposal | D | 6 | 1 | Collection and transport to final end-of-life location | occupational | dermal/eye |
| Disposal | D | 6 | 2 | Collection and transport to final end-of-life location | environmental | direct |
| Disposal | D | 6 | 3 | Incineration or heat recovery | occupational | inhalation |
| Disposal | D | 6 | 4 | Incineration or heat recovery | environmental | direct |
| Disposal | D | 6 | 5 | Long-term MSW landfill storage | environmental | direct |
| Disposal | D | 6 | 6 | Food packaging discarded in uncontrolled environment | environmental | direct |

**Legend:** LC Code, life-cycle code; LCS #, life-cycle stage number; RM, raw material; PM, product manufacturing; PA, product application; PU, product use; RR, re-use/recycling; D, disposal; CN, cellulose nanomaterial; SCNF, sulfated cellulose nanofibers.

**Table S10.** Exposure scenarios for CS3: food additive [TCNF, PCCNF (carboxyl functionalization)].

| Life-Cycle Stage | LC Code | LCS # | Scenario # | Scenario | Receptor | Exposure Route |
|---|---|---|---|---|---|---|
| Raw Material | RM | 1 | 1 | Harvesting, chipping/shredding, pulping softwood or hardwood | occupational | inhalation |
| Product Manufacturing | PM | 2 | 1 | Cleaning out synthesis equipment | occupational | inhalation |
| Product Manufacturing | PM | 2 | 2 | Cleaning out synthesis equipment | occupational | dermal/eye |
| Product Manufacturing | PM | 2 | 3 | Cleaning out synthesis equipment | environmental | direct |
| Product Manufacturing | PM | 2 | 4 | Incidental release of CN from synthesis equipment | occupational | inhalation |
| Product Manufacturing | PM | 2 | 5 | Incidental release of CN from synthesis equipment | occupational | dermal/eye |
| Product Manufacturing | PM | 2 | 6 | Incidental release of CN from synthesis equipment | environmental | direct |
| Product Manufacturing | PM | 2 | 7 | Accidental spill of CN from synthesis equipment | occupational | inhalation |
| Product Manufacturing | PM | 2 | 8 | Accidental spill of CN from synthesis equipment | occupational | dermal/eye |
| Product Manufacturing | PM | 2 | 9 | Accidental spill of CN from synthesis equipment | environmental | direct |
| Product Manufacturing | PM | 2 | 10 | Dried formulation extraction and handling (for powder CN ingredients) | occupational | inhalation |
| Product Manufacturing | PM | 2 | 11 | Dried formulation extraction and handling (for powder CN ingredients) | occupational | dermal/eye |
| Product Application | PA | 3 | 1 | Transfer CN from synthesis to application facility (e.g. handling, packaging) | occupational | inhalation |
| Product Application | PA | 3 | 2 | Transfer CN from synthesis to application facility (e.g. handling, packaging) | occupational | dermal/eye |
| Product Application | PA | 3 | 3 | Food manufacturing equipment cleanout | occupational | inhalation |
| Product Application | PA | 3 | 4 | Food manufacturing equipment cleanout | occupational | dermal/eye |
| Product Application | PA | 3 | 5 | Food manufacturing equipment cleanout | environmental | direct |
| Product Application | PA | 3 | 6 | CN rehydration (if powder CN ingredient) | occupational | inhalation |
| Product Application | PA | 3 | 7 | CN rehydration (if powder CN ingredient) | occupational | dermal/eye |
| Product Application | PA | 3 | 8 | CN containing food formulation & preparation (e.g. high shear mixing with other ingredients, homogenixing, other) | occupational | inhalation |
| Product Application | PA | 3 | 9 | CN containing food formulation & preparation (e.g. high shear mixing with other ingredients, homogenixing, other) | occupational | dermal/eye |
| Product Application | PA | 3 | 10 | Incidental release of CN from manufacturing equipment | occupational | inhalation |
| Product Application | PA | 3 | 11 | Incidental release of CN from manufacturing equipment | occupational | dermal/eye |
| Product Application | PA | 3 | 12 | Incidental release of CN from manufacturing equipment | environmental | direct |
| Product Application | PA | 3 | 13 | Accidental spill of CN from manufacturing equipment | occupational | inhalation |
| Product Application | PA | 3 | 14 | Accidental spill of CN from manufacturing equipment | occupational | dermal/eye |
| Product Application | PA | 3 | 15 | Accidental spill of CN from manufacturing equipment | environmental | direct |
| Product Application | PA | 3 | 16 | Packaging and handling of CN-containing food | occupational | inhalation |
| Product Application | PA | 3 | 17 | Packaging and handling of CN-containing food | occupational | dermal/eye |

| | | | | | | |
|---|---|---|---|---|---|---|
| Product Use | PU | 4 | 1 | Food handling and consumption | consumer | ingestion |
| Product Use | PU | 4 | 2 | Food handling and consumption | consumer | dermal/eye |
| Re-use/Recycling | RR | 5 | 1 | Collection and transport of waste food to re-use or compost facility | occupational | dermal/eye |
| Re-use/Recycling | RR | 5 | 2 | Production of food waste into animal feed | environmental | direct |
| Re-use/Recycling | RR | 5 | 3 | Composting CN containing food | consumer | inhalation |
| Re-use/Recycling | RR | 5 | 4 | Composting CN containing food | consumer | dermal/eye |
| Re-use/Recycling | RR | 5 | 5 | Composting CN containing food | occupational | inhalation |
| Re-use/Recycling | RR | 5 | 6 | Composting CN containing food | occupational | dermal/eye |
| Re-use/Recycling | RR | 5 | 7 | Composting CN containing food | environmental | direct |
| Re-use/Recycling | RR | 5 | 8 | Bio-conversion (anaerobic digestion) | occupational | inhalation |
| Re-use/Recycling | RR | 5 | 9 | Bio-conversion (anaerobic digestion) | occupational | dermal/eye |
| Re-use/Recycling | RR | 5 | 10 | Bio-conversion (anaerobic digestion) | environmental | direct |
| Disposal | D | 6 | 1 | Collection and transport to final end-of-life location | occupational | dermal/eye |
| Disposal | D | 6 | 2 | Collection and transport to final end-of-life location | environmental | direct |
| Disposal | D | 6 | 3 | Incineration or heat recovery | occupational | inhalation |
| Disposal | D | 6 | 4 | Incineration or heat recovery | environmental | direct |
| Disposal | D | 6 | 5 | Long-term MSW landfill storage | environmental | direct |
| Disposal | D | 6 | 6 | Food discarded in uncontrolled environment | environmental | direct |

**Legend:** LC Code, life-cycle code; LCS #, life-cycle stage number; RM, raw material; PM, product manufacturing; PA, product application; PU, product use; RR, re-use/recycling; D, disposal; CN, cellulose nanomaterial; TCNFs, TEMPO-oxidized cellulose nanofibers; PCCNFs, periodate-chlorite oxidized cellulose nanofibers.

## 3. Exposure Scenario Ranking

Exposure scenario ranking for CS1: water filtration membrane [TCNF, PCCNF (carboxyl functionalization)] can be found in Table 5.

**Table S11.** Exposure scenario ranking for CS2.1: carboxylated CNF/CNC food packaging film.

| Life-Cycle Stage | LCSC | LCSN | SN | Scenario | Receptor | ER | DE | M | L | F | Score | Rank |
|---|---|---|---|---|---|---|---|---|---|---|---|---|
| Product Manufacturing | PM | 2 | 10 | Dried formulation extraction and handling (for powder CN ingredients) | occupational | inhalation | 3 | 3 | 3 | 3 | 12 | 1 |
| Product Manufacturing | PM | 2 | 11 | Dried formulation extraction and handling (for powder CN ingredients) | occupational | ingestion/dermal/eye | 3 | 3 | 3 | 3 | 12 | 1 |
| Product Application | PA | 3 | 1 | Transfer CN from synthesis to application facility (e.g. handling, packaging) | occupational | inhalation | 3 | 3 | 3 | 3 | 12 | 1 |
| Product Application | PA | 3 | 2 | Transfer CN from synthesis to application facility (e.g. handling, packaging) | occupational | ingestion/dermal/eye | 3 | 3 | 3 | 3 | 12 | 1 |
| Product Application | PA | 3 | 6 | CN rehydration (if powder CN ingredient) | occupational | inhalation | 3 | 3 | 3 | 3 | 12 | 1 |
| Product Application | PA | 3 | 7 | CN rehydration (if powder CN ingredient) | occupational | ingestion/dermal/eye | 3 | 3 | 3 | 3 | 12 | 1 |
| Product Application | PA | 3 | 19 | CN drying (film formation) | occupational | ingestion/dermal/eye | 2 | 3 | 3 | 3 | 11 | 2 |
| Product Application | PA | 3 | 21 | Surface treatment of CN film (e.g., hot press, wax coating, other) | occupational | ingestion/dermal/eye | 2 | 3 | 3 | 3 | 11 | 2 |
| Product Application | PA | 3 | 23 | Physical treatment of CN film (e.g., forming, bending, other) | occupational | ingestion/dermal/eye | 2 | 3 | 3 | 3 | 11 | 2 |
| Product Use | PU | 4 | 1 | Food packaging use (release, migration to food) | consumer | ingestion | 2 | 3 | 3 | 3 | 11 | 2 |
| Product Use | PU | 4 | 3 | Food packaging handling/interaction (release) | consumer | dermal/eye | 2 | 3 | 3 | 3 | 11 | 2 |
| Re-use/Recycling | RR | 5 | 2 | Collection and transport of used food packaging to recycling, re-use or compost facility | occupational | ingestion/dermal/eye | 2 | 3 | 3 | 3 | 11 | 2 |
| Re-use/Recycling | RR | 5 | 4 | Recycling activities: Physical breakdown of used food packaging (e.g. tearing) | occupational | ingestion/dermal/eye | 2 | 3 | 3 | 3 | 11 | 2 |
| Re-use/Recycling | RR | 5 | 6 | Recycling activities: Chemical breakdown of used food packaging (e.g. pulping) | occupational | ingestion/dermal/eye | 2 | 3 | 3 | 3 | 11 | 2 |
| Re-use/Recycling | RR | 5 | 11 | Composting used food packaging | environmental | direct | 2 | 3 | 3 | 3 | 11 | 2 |
| Disposal | D | 6 | 5 | Long-term MSW landfill storage | environmental | direct | 2 | 3 | 3 | 3 | 11 | 2 |
| Product Manufacturing | PM | 2 | 1 | Cleaning out synthesis equipment | occupational | inhalation | 2 | 2 | 3 | 3 | 10 | 3 |
| Product Manufacturing | PM | 2 | 2 | Cleaning out synthesis equipment | occupational | ingestion/dermal/eye | 2 | 2 | 3 | 3 | 10 | 3 |
| Product Application | PA | 3 | 3 | Deposition and formulation equipment cleanout | occupational | inhalation | 2 | 2 | 3 | 3 | 10 | 3 |
| Product Application | PA | 3 | 4 | Deposition and formulation equipment cleanout | occupational | ingestion/dermal/eye | 2 | 2 | 3 | 3 | 10 | 3 |

| | | | | | | | | | | | | |
|---|---|---|---|---|---|---|---|---|---|---|---|---|
| Product Application | PA | 3 | 8 | CN film formulation preparation (e.g. mixing with other ingredients, homogenixing, other) | occupational | inhalation | 2 | 2 | 3 | 3 | 10 | 3 |
| Product Application | PA | 3 | 9 | CN film formulation preparation (e.g. mixing with other ingredients, homogenixing, other) | occupational | ingestion/dermal/eye | 2 | 2 | 3 | 3 | 10 | 3 |
| Product Application | PA | 3 | 10 | CN casting to form film | occupational | inhalation | 2 | 2 | 3 | 3 | 10 | 3 |
| Product Application | PA | 3 | 11 | CN casting to form film | occupational | ingestion/dermal/eye | 2 | 2 | 3 | 3 | 10 | 3 |
| Re-use/Recycling | RR | 5 | 10 | Composting used food packaging | occupational | dermal/eye | 2 | 3 | 2 | 3 | 10 | 3 |
| Disposal | D | 6 | 1 | Collection and transport to final end-of-life location | occupational | ingestion/dermal/eye | 2 | 3 | 2 | 3 | 10 | 3 |
| Disposal | D | 6 | 2 | Collection and transport to final end-of-life location | environmental | direct | 2 | 3 | 2 | 3 | 10 | 3 |
| Disposal | D | 6 | 6 | Food packaging discarded in uncontrolled environment | environmental | direct | 2 | 3 | 2 | 3 | 10 | 3 |
| Product Application | PA | 3 | 18 | CN drying (film formation) | occupational | inhalation | 2 | 3 | 1 | 3 | 9 | 4 |
| Product Application | PA | 3 | 20 | Surface treatment of CN film (e.g., hot press, wax coating, other) | occupational | inhalation | 2 | 3 | 1 | 3 | 9 | 4 |
| Product Application | PA | 3 | 22 | Physical treatment of CN film (e.g., forming, bending, other) | occupational | inhalation | 2 | 3 | 1 | 3 | 9 | 4 |
| Product Use | PU | 4 | 2 | Food packaging handling/interaction (release) | consumer | inhalation | 2 | 3 | 1 | 3 | 9 | 4 |
| Re-use/Recycling | RR | 5 | 1 | Collection and transport of used food packaging to recycling, re-use or compost facility | occupational | inhalation | 2 | 3 | 1 | 3 | 9 | 4 |
| Re-use/Recycling | RR | 5 | 3 | Recycling activities: Physical breakdown of used food packaging (e.g. tearing) | occupational | inhalation | 2 | 3 | 1 | 3 | 9 | 4 |
| Re-use/Recycling | RR | 5 | 5 | Recycling activities: Chemical breakdown of used food packaging (e.g. pulping) | occupational | inhalation | 2 | 3 | 1 | 3 | 9 | 4 |
| Product Manufacturing | PM | 2 | 4 | Incidental release of CN from synthesis equipment | occupational | inhalation | 2 | 2 | 2 | 2 | 8 | 5 |
| Product Manufacturing | PM | 2 | 5 | Incidental release of CN from synthesis equipment | occupational | ingestion/dermal/eye | 2 | 2 | 2 | 2 | 8 | 5 |
| Product Manufacturing | PM | 2 | 6 | Incidental release of CN from synthesis equipment | environmental | direct | 2 | 2 | 2 | 2 | 8 | 5 |
| Product Application | PA | 3 | 12 | Incidental release of CN from deposition equipment | occupational | inhalation | 2 | 2 | 2 | 2 | 8 | 5 |
| Product Application | PA | 3 | 13 | Incidental release of CN from deposition equipment | occupational | ingestion/dermal/eye | 2 | 2 | 2 | 2 | 8 | 5 |
| Product Use | PU | 4 | 5 | Misuse: Degraded food packaging | consumer | dermal/eye | 2 | 3 | 2 | 1 | 8 | 5 |
| Product Use | PU | 4 | 6 | Misuse: Degraded food packaging | consumer | ingestion | 2 | 3 | 2 | 1 | 8 | 5 |
| Product Use | PU | 4 | 7 | Misuse: Degraded food packaging | environmental | direct | 2 | 3 | 2 | 1 | 8 | 5 |
| Re-use/Recycling | RR | 5 | 8 | Composting used food packaging | consumer | ingestion/dermal/eye | 2 | 3 | 2 | 1 | 8 | 5 |
| Re-use/Recycling | RR | 5 | 13 | Bioconversion (anaerobic digestion) | occupational | ingestion/dermal/eye | 2 | 3 | 2 | 1 | 8 | 5 |
| Re-use/Recycling | RR | 5 | 14 | Bioconversion (anaerobic digestion) | environmental | direct | 2 | 3 | 2 | 1 | 8 | 5 |
| Disposal | D | 6 | 3 | Incineration or heat recovery | occupational | inhalation | 2 | 3 | 2 | 1 | 8 | 5 |
| Disposal | D | 6 | 4 | Incineration or heat recovery | environmental | direct | 2 | 3 | 2 | 1 | 8 | 5 |

| | | | | | | | | | | | | |
|---|---|---|---|---|---|---|---|---|---|---|---|---|
| Product Manufacturing | PM | 2 | 3 | Cleaning out synthesis equipment | environmental | direct | 2 | 2 | 2 | 1 | 7 | 6 |
| Product Manufacturing | PM | 2 | 7 | Accidental spill of CN from synthesis equipment | occupational | inhalation | 2 | 2 | 2 | 1 | 7 | 6 |
| Product Manufacturing | PM | 2 | 8 | Accidental spill of CN from synthesis equipment | occupational | ingestion/dermal/eye | 2 | 2 | 2 | 1 | 7 | 6 |
| Product Manufacturing | PM | 2 | 9 | Accidental spill of CN from synthesis equipment | environmental | direct | 2 | 2 | 2 | 1 | 7 | 6 |
| Product Application | PA | 3 | 5 | Deposition and formulation equipment cleanout | environmental | direct | 2 | 2 | 2 | 1 | 7 | 6 |
| Product Application | PA | 3 | 14 | Incidental release of CN from deposition equipment | environmental | direct | 2 | 2 | 1 | 2 | 7 | 6 |
| Product Application | PA | 3 | 15 | Accidental spill of CN from deposition equipment | occupational | inhalation | 2 | 2 | 2 | 1 | 7 | 6 |
| Product Application | PA | 3 | 16 | Accidental spill of CN from deposition equipment | occupational | ingestion/dermal/eye | 2 | 2 | 2 | 1 | 7 | 6 |
| Product Application | PA | 3 | 17 | Accidental spill of CN from deposition equipment | environmental | direct | 2 | 2 | 2 | 1 | 7 | 6 |
| Product Use | PU | 4 | 4 | Misuse: Degraded food packaging | consumer | inhalation | 2 | 3 | 1 | 1 | 7 | 6 |
| Re-use/Recycling | RR | 5 | 7 | Composting used food packaging | consumer | inhalation | 2 | 3 | 1 | 1 | 7 | 6 |
| Re-use/Recycling | RR | 5 | 9 | Composting used food packaging | occupational | inhalation | 2 | 3 | 1 | 1 | 7 | 6 |
| Re-use/Recycling | RR | 5 | 12 | Bioconversion (anaerobic digestion) | occupational | inhalation | 2 | 3 | 1 | 1 | 7 | 6 |
| Raw Material | RM | 1 | 1 | Harvesting, chipping/shredding, pulping softwood or hardwood | occupational | inhalation | | | | | 0 | 7 |

**Legend:** LCSC, Life-Cycle Stage Code; LCSN, Life-Cycle Stage Number; SN, Scenario Number; ER, Exposure Route; DE, Directness of Exposure; M, Magnitude; L, Likelihood; F, Frequency; RM, raw material; PM, product manufacturing; PA, product application; PU, product use; RR, re-use/recycling; D, disposal; MSW; municipal solid waste; CN, cellulose nanomaterial; CNFs, cellulose nanofibers; CNC, cellulose nanocrystals.

**Table S12.** Exposure scenario ranking for CS2.2: sulfated CNF/CNC food packaging coating.

| Life-Cycle Stage | LCSC | LCSN | SN | Scenario | Receptor | ER | DE | M | L | F | Score | Rank |
|---|---|---|---|---|---|---|---|---|---|---|---|---|
| Product Manufacturing | PM | 2 | 10 | Dried formulation extraction and handling (for powder CN ingredients) | occupational | inhalation | 3 | 3 | 3 | 3 | 12 | 1 |
| Product Manufacturing | PM | 2 | 11 | Dried formulation extraction and handling (for powder CN ingredients) | occupational | ingestion/dermal/eye | 3 | 3 | 3 | 3 | 12 | 1 |
| Product Application | PA | 3 | 1 | Transfer CN from synthesis to application facility (e.g. handling, packaging) | occupational | inhalation | 3 | 3 | 3 | 3 | 12 | 1 |
| Product Application | PA | 3 | 2 | Transfer CN from synthesis to application facility (e.g. handling, packaging) | occupational | ingestion/dermal/eye | 3 | 3 | 3 | 3 | 12 | 1 |
| Product Application | PA | 3 | 6 | CN rehydration (if powder NC ingredient) | occupational | inhalation | 3 | 3 | 3 | 3 | 12 | 1 |
| Product Application | PA | 3 | 7 | CN rehydration (if powder NC ingredient) | occupational | ingestion/dermal/eye | 3 | 3 | 3 | 3 | 12 | 1 |
| Product Application | PA | 3 | 19 | CN coating dried on paper substrate (coating formation) | occupational | ingestion/dermal/eye | 2 | 3 | 3 | 3 | 11 | 2 |
| Product Application | PA | 3 | 21 | Surface treatment of CN coating (hot press, other) | occupational | ingestion/dermal/eye | 2 | 3 | 3 | 3 | 11 | 2 |
| Product Application | PA | 3 | 23 | Physical treatment of CN coating (e.g. forming, bending, other) | occupational | ingestion/dermal/eye | 2 | 3 | 3 | 3 | 11 | 2 |
| Product Use | PU | 4 | 1 | Food packaging use (release, migration) | consumer | ingestion | 2 | 3 | 3 | 3 | 11 | 2 |
| Product Use | PU | 4 | 3 | Food packaging handling/interaction (release) | consumer | ingestion/dermal/eye | 2 | 3 | 3 | 3 | 11 | 2 |
| Re-use/Recycling | RR | 5 | 2 | Collection and transport of used food packaging to recycling, re-use or compost facility | occupational | ingestion/dermal/eye | 2 | 3 | 3 | 3 | 11 | 2 |
| Re-use/Recycling | RR | 5 | 4 | Recycling activities: Physical breakdown of used food packaging (e.g. tearing) | occupational | ingestion/dermal/eye | 2 | 3 | 3 | 3 | 11 | 2 |
| Re-use/Recycling | RR | 5 | 6 | Recycling activities: Chemical breakdown of used food packaging (e.g. pulping) | occupational | ingestion/dermal/eye | 2 | 3 | 3 | 3 | 11 | 2 |
| Re-use/Recycling | RR | 5 | 11 | Composting used food packaging | environmental | direct | 2 | 3 | 3 | 3 | 11 | 2 |
| Disposal | D | 6 | 5 | Long-term MSW landfill storage | environmental | direct | 2 | 3 | 3 | 3 | 11 | 2 |
| Product Manufacturing | PM | 2 | 1 | Cleaning out synthesis equipment | occupational | inhalation | 2 | 2 | 3 | 3 | 10 | 3 |
| Product Manufacturing | PM | 2 | 2 | Cleaning out synthesis equipment | occupational | ingestion/dermal/eye | 2 | 2 | 3 | 3 | 10 | 3 |
| Product Application | PA | 3 | 3 | Deposition (and formulation prep) equipment cleanout | occupational | inhalation | 2 | 2 | 3 | 3 | 10 | 3 |
| Product Application | PA | 3 | 4 | Deposition (and formulation prep) equipment cleanout | occupational | ingestion/dermal/eye | 2 | 2 | 3 | 3 | 10 | 3 |
| Product Application | PA | 3 | 8 | CN coating formulation preparation (e.g. mixing with other ingredients, homogenixing, other) | occupational | inhalation | 2 | 2 | 3 | 3 | 10 | 3 |
| Product Application | PA | 3 | 9 | CN coating formulation preparation (e.g. mixing with other ingredients, homogenixing, other) | occupational | ingestion/dermal/eye | 2 | 2 | 3 | 3 | 10 | 3 |

| | | | | | | | | | | | | |
|---|---|---|---|---|---|---|---|---|---|---|---|---|
| Product Application | PA | 3 | 10 | CN formulation applied via spraying coating on paper food contact material | occupational | inhalation | 2 | 2 | 3 | 3 | 10 | 3 |
| Product Application | PA | 3 | 11 | CN formulation applied via spraying coating on paper food contact material | occupational | ingestion/dermal/eye | 2 | 2 | 3 | 3 | 10 | 3 |
| Re-use/Recycling | RR | 5 | 10 | Composting used food packaging | occupational | ingestion/dermal/eye | 2 | 3 | 2 | 3 | 10 | 3 |
| Disposal | D | 6 | 1 | Collection and transport to final end-of-life location | occupational | ingestion/dermal/eye | 2 | 3 | 2 | 3 | 10 | 3 |
| Disposal | D | 6 | 2 | Collection and transport to final end-of-life location | environmental | direct | 2 | 3 | 2 | 3 | 10 | 3 |
| Disposal | D | 6 | 6 | Food packaging discarded in uncontrolled environment | environmental | direct | 2 | 3 | 2 | 3 | 10 | 3 |
| Product Application | PA | 3 | 18 | CN coating dried on paper substrate (coating formation) | occupational | inhalation | 2 | 3 | 1 | 3 | 9 | 4 |
| Product Application | PA | 3 | 20 | Surface treatment of CN coating (hot press, other) | occupational | inhalation | 2 | 3 | 1 | 3 | 9 | 4 |
| Product Application | PA | 3 | 22 | Physical treatment of CN coating (e.g. forming, bending, other) | occupational | inhalation | 2 | 3 | 1 | 3 | 9 | 4 |
| Product Use | PU | 4 | 2 | Food packaging handling/interaction (release) | consumer | inhalation | 2 | 3 | 1 | 3 | 9 | 4 |
| Re-use/Recycling | RR | 5 | 1 | Collection and transport of used food packaging to recycling, re-use or compost facility | occupational | inhalation | 2 | 3 | 1 | 3 | 9 | 4 |
| Re-use/Recycling | RR | 5 | 3 | Recycling activities: Physical breakdown of used food packaging (e.g. tearing) | occupational | inhalation | 2 | 3 | 1 | 3 | 9 | 4 |
| Re-use/Recycling | RR | 5 | 5 | Recycling activities: Chemical breakdown of used food packaging (e.g. pulping) | occupational | inhalation | 2 | 3 | 1 | 3 | 9 | 4 |
| Product Manufacturing | PM | 2 | 4 | Incidental spill of CN from synthesis equipment | occupational | inhalation | 2 | 2 | 2 | 2 | 8 | 5 |
| Product Manufacturing | PM | 2 | 5 | Incidental spill of CN from synthesis equipment | occupational | ingestion/dermal/eye | 2 | 2 | 2 | 2 | 8 | 5 |
| Product Manufacturing | PM | 2 | 6 | Incidental spill of CN from synthesis equipment | environmental | direct | 2 | 2 | 2 | 2 | 8 | 5 |
| Product Application | PA | 3 | 12 | Incidental release of CN from deposition equipment | occupational | inhalation | 2 | 2 | 2 | 2 | 8 | 5 |
| Product Application | PA | 3 | 13 | Incidental release of CN from deposition equipment | occupational | ingestion/dermal/eye | 2 | 2 | 2 | 2 | 8 | 5 |
| Product Use | PU | 4 | 5 | Misuse: Degraded food packaging | consumer | dermal/eye | 2 | 3 | 2 | 1 | 8 | 5 |
| Product Use | PU | 4 | 6 | Misuse: Degraded food packaging | consumer | ingestion | 2 | 3 | 2 | 1 | 8 | 5 |
| Product Use | PU | 4 | 7 | Misuse: Degraded food packaging | environmental | direct | 2 | 3 | 2 | 1 | 8 | 5 |
| Re-use/Recycling | RR | 5 | 8 | Composting used food packaging | consumer | ingestion/dermal/eye | 2 | 3 | 2 | 1 | 8 | 5 |
| Re-use/Recycling | RR | 5 | 13 | Bio-conversion (anaerobic digestion) | occupational | ingestion/dermal/eye | 2 | 3 | 2 | 1 | 8 | 5 |
| Re-use/Recycling | RR | 5 | 14 | Bio-conversion (anaerobic digestion) | environmental | direct | 2 | 3 | 2 | 1 | 8 | 5 |
| Disposal | D | 6 | 3 | Incineration or heat recovery | occupational | inhalation | 2 | 3 | 2 | 1 | 8 | 5 |
| Disposal | D | 6 | 4 | Incineration or heat recovery | environmental | direct | 2 | 3 | 2 | 1 | 8 | 5 |
| Product Manufacturing | PM | 2 | 3 | Cleaning out synthesis equipment | environmental | direct | 2 | 1 | 1 | 3 | 7 | 6 |
| Product Manufacturing | PM | 2 | 7 | Accidental spill of CN from synthesis equipment | occupational | inhalation | 2 | 2 | 2 | 1 | 7 | 6 |
| Product Manufacturing | PM | 2 | 8 | Accidental spill of CN from synthesis equipment | occupational | ingestion/dermal/eye | 2 | 2 | 2 | 1 | 7 | 6 |

| | | | | | | | | | | | | |
|---|---|---|---|---|---|---|---|---|---|---|---|---|
| Product Manufacturing | PM | 2 | 9 | Accidental spill of CN from synthesis equipment | environmental | direct | 2 | 2 | 2 | 1 | 7 | 6 |
| Product Application | PA | 3 | 5 | Deposition (and formulation prep) equipment cleanout | environmental | direct | 2 | 2 | 2 | 1 | 7 | 6 |
| Product Application | PA | 3 | 14 | Incidental release of CN from deposition equipment | environmental | direct | 2 | 2 | 1 | 2 | 7 | 6 |
| Product Application | PA | 3 | 15 | Accidental spill of CN from deposition equipment | occupational | inhalation | 2 | 2 | 2 | 1 | 7 | 6 |
| Product Application | PA | 3 | 16 | Accidental spill of CN from deposition equipment | occupational | ingestion/dermal/eye | 2 | 2 | 2 | 1 | 7 | 6 |
| Product Application | PA | 3 | 17 | Accidental spill of CN from deposition equipment | environmental | direct | 2 | 2 | 2 | 1 | 7 | 6 |
| Product Use | PU | 4 | 4 | Misuse: Degraded food packaging | consumer | inhalation | 2 | 3 | 1 | 1 | 7 | 6 |
| Re-use/Recycling | RR | 5 | 7 | Composting used food packaging | consumer | inhalation | 2 | 3 | 1 | 1 | 7 | 6 |
| Re-use/Recycling | RR | 5 | 9 | Composting used food packaging | occupational | inhalation | 2 | 3 | 1 | 1 | 7 | 6 |
| Re-use/Recycling | RR | 5 | 12 | Bio-conversion (anaerobic digestion) | occupational | inhalation | 2 | 3 | 1 | 1 | 7 | 6 |
| Raw Material | RM | 1 | 1 | Harvesting, chipping/shredding, pulping softwood or hardwood | occupational | inhalation | | | | | 0 | 7 |

**Legend:** LCSC, Life-Cycle Stage Code; LCSN, Life-Cycle Stage Number; SN, Scenario Number; ER, Exposure Route; DE, Directness of Exposure; M, Magnitude; L, Likelihood; F, Frequency; RM, raw material; PM, product manufacturing; PA, product application; PU, product use; RR, re-use/recycling; D, disposal; MSW, municipal solid waste; CN, cellulose nanomaterial; CNFs, cellulose nanofibers; CNC, cellulose nanocrystals.

**Table S13.** Exposure scenario ranking for CS2.3: sulfated CNF/CNC food packaging additive.

| Life-Cycle Stage | LCSC | LCSN | SN | Scenario | Receptor | ER | DE | M | L | F | Score | Rank |
|---|---|---|---|---|---|---|---|---|---|---|---|---|
| Product Manufacturing | PM | 2 | 10 | Dried formulation extraction and handling (for powder CN ingredients) | occupational | inhalation | 3 | 3 | 3 | 3 | 12 | 1 |
| Product Manufacturing | PM | 2 | 11 | Dried formulation extraction and handling (for powder CN ingredients) | occupational | ingestion/dermal/eye | 3 | 3 | 3 | 3 | 12 | 1 |
| Product Application | PA | 3 | 1 | Transfer CN from synthesis to application facility (e.g. handling, packaging) | occupational | inhalation | 3 | 3 | 3 | 3 | 12 | 1 |
| Product Application | PA | 3 | 2 | Transfer CN from synthesis to application facility (e.g. handling, packaging) | occupational | ingestion/dermal/eye | 3 | 3 | 3 | 3 | 12 | 1 |
| Product Application | PA | 3 | 6 | CN rehydration (if powder NC ingredient) | occupational | inhalation | 3 | 3 | 3 | 3 | 12 | 1 |
| Product Application | PA | 3 | 7 | CN rehydration (if powder NC ingredient) | occupational | ingestion/dermal/eye | 3 | 3 | 3 | 3 | 12 | 1 |
| Product Application | PA | 3 | 19 | CN coating dried on paper substrate (coating formation) | occupational | ingestion/dermal/eye | 2 | 3 | 3 | 3 | 11 | 2 |
| Product Application | PA | 3 | 21 | Surface treatment of CN coating (hot press, other) | occupational | ingestion/dermal/eye | 2 | 3 | 3 | 3 | 11 | 2 |
| Product Application | PA | 3 | 23 | Physical treatment of CN coating (e.g. forming, bending, other) | occupational | ingestion/dermal/eye | 2 | 3 | 3 | 3 | 11 | 2 |
| Product Use | PU | 4 | 1 | Food packaging use (release, migration) | consumer | ingestion | 2 | 3 | 3 | 3 | 11 | 2 |
| Product Use | PU | 4 | 3 | Food packaging handling/interaction (release) | consumer | ingestion/dermal/eye | 2 | 3 | 3 | 3 | 11 | 2 |
| Re-use/Recycling | RR | 5 | 2 | Collection and transport of used food packaging to recycling, re-use or compost facility | occupational | ingestion/dermal/eye | 2 | 3 | 3 | 3 | 11 | 2 |
| Re-use/Recycling | RR | 5 | 4 | Recycling activities: Physical breakdown of used food packaging (e.g. tearing) | occupational | ingestion/dermal/eye | 2 | 3 | 3 | 3 | 11 | 2 |
| Re-use/Recycling | RR | 5 | 6 | Recycling activities: Chemical breakdown of used food packaging (e.g. pulping) | occupational | ingestion/dermal/eye | 2 | 3 | 3 | 3 | 11 | 2 |
| Re-use/Recycling | RR | 5 | 11 | Composting used food packaging | environmental | direct | 2 | 3 | 3 | 3 | 11 | 2 |
| Disposal | D | 6 | 5 | Long-term MSW landfill storage | environmental | direct | 2 | 3 | 3 | 3 | 11 | 2 |
| Product Manufacturing | PM | 2 | 1 | Cleaning out synthesis equipment | occupational | inhalation | 2 | 2 | 3 | 3 | 10 | 3 |
| Product Manufacturing | PM | 2 | 2 | Cleaning out synthesis equipment | occupational | ingestion/dermal/eye | 2 | 2 | 3 | 3 | 10 | 3 |
| Product Application | PA | 3 | 3 | Deposition (and formulation prep) equipment cleanout | occupational | inhalation | 2 | 2 | 3 | 3 | 10 | 3 |
| Product Application | PA | 3 | 4 | Deposition (and formulation prep) equipment cleanout | occupational | ingestion/dermal/eye | 2 | 2 | 3 | 3 | 10 | 3 |
| Product Application | PA | 3 | 8 | CN coating formulation preparation (e.g. mixing with other ingredients, homogenixing, other) | occupational | inhalation | 2 | 2 | 3 | 3 | 10 | 3 |
| Product Application | PA | 3 | 9 | CN coating formulation preparation (e.g. mixing with other ingredients, homogenixing, other) | occupational | ingestion/dermal/eye | 2 | 2 | 3 | 3 | 10 | 3 |

| | | | | | | | | | | | | |
|---|---|---|---|---|---|---|---|---|---|---|---|---|
| Product Application | PA | 3 | 10 | CN formulation applied via spraying coating on paper food contact material | occupational | inhalation | 2 | 2 | 3 | 3 | 10 | 3 |
| Product Application | PA | 3 | 11 | CN formulation applied via spraying coating on paper food contact material | occupational | ingestion/dermal/eye | 2 | 2 | 3 | 3 | 10 | 3 |
| Re-use/Recycling | RR | 5 | 10 | Composting used food packaging | occupational | ingestion/dermal/eye | 2 | 3 | 2 | 3 | 10 | 3 |
| Disposal | D | 6 | 1 | Collection and transport to final end-of-life location | occupational | ingestion/dermal/eye | 2 | 3 | 2 | 3 | 10 | 3 |
| Disposal | D | 6 | 2 | Collection and transport to final end-of-life location | environmental | direct | 2 | 3 | 2 | 3 | 10 | 3 |
| Disposal | D | 6 | 6 | Food packaging discarded in uncontrolled environment | environmental | direct | 2 | 3 | 2 | 3 | 10 | 3 |
| Product Application | PA | 3 | 18 | CN coating dried on paper substrate (coating formation) | occupational | inhalation | 2 | 3 | 1 | 3 | 9 | 4 |
| Product Application | PA | 3 | 20 | Surface treatment of CN coating (hot press, other) | occupational | inhalation | 2 | 3 | 1 | 3 | 9 | 4 |
| Product Application | PA | 3 | 22 | Physical treatment of CN coating (e.g. forming, bending, other) | occupational | inhalation | 2 | 3 | 1 | 3 | 9 | 4 |
| Product Use | PU | 4 | 2 | Food packaging handling/interaction (release) | consumer | inhalation | 2 | 3 | 1 | 3 | 9 | 4 |
| Re-use/Recycling | RR | 5 | 1 | Collection and transport of used food packaging to recycling, re-use or compost facility | occupational | inhalation | 2 | 3 | 1 | 3 | 9 | 4 |
| Re-use/Recycling | RR | 5 | 3 | Recycling activities: Physical breakdown of used food packaging (e.g. tearing) | occupational | inhalation | 2 | 3 | 1 | 3 | 9 | 4 |
| Re-use/Recycling | RR | 5 | 5 | Recycling activities: Chemical breakdown of used food packaging (e.g. pulping) | occupational | inhalation | 2 | 3 | 1 | 3 | 9 | 4 |
| Product Manufacturing | PM | 2 | 4 | Incidental spill of CN from synthesis equipment | occupational | inhalation | 2 | 2 | 2 | 2 | 8 | 5 |
| Product Manufacturing | PM | 2 | 5 | Incidental spill of CN from synthesis equipment | occupational | ingestion/dermal/eye | 2 | 2 | 2 | 2 | 8 | 5 |
| Product Manufacturing | PM | 2 | 6 | Incidental spill of CN from synthesis equipment | environmental | direct | 2 | 2 | 2 | 2 | 8 | 5 |
| Product Application | PA | 3 | 12 | Incidental release of CN from deposition equipment | occupational | inhalation | 2 | 2 | 2 | 2 | 8 | 5 |
| Product Application | PA | 3 | 13 | Incidental release of CN from deposition equipment | occupational | ingestion/dermal/eye | 2 | 2 | 2 | 2 | 8 | 5 |
| Product Use | PU | 4 | 5 | Misuse: Degraded food packaging | consumer | dermal/eye | 2 | 3 | 2 | 1 | 8 | 5 |
| Product Use | PU | 4 | 6 | Misuse: Degraded food packaging | consumer | ingestion | 2 | 3 | 2 | 1 | 8 | 5 |
| Product Use | PU | 4 | 7 | Misuse: Degraded food packaging | environmental | direct | 2 | 3 | 2 | 1 | 8 | 5 |
| Re-use/Recycling | RR | 5 | 8 | Composting used food packaging | consumer | ingestion/dermal/eye | 2 | 3 | 2 | 1 | 8 | 5 |
| Re-use/Recycling | RR | 5 | 13 | Bio-conversion (anaerobic digestion) | occupational | ingestion/dermal/eye | 2 | 3 | 2 | 1 | 8 | 5 |
| Re-use/Recycling | RR | 5 | 14 | Bio-conversion (anaerobic digestion) | environmental | direct | 2 | 3 | 2 | 1 | 8 | 5 |
| Disposal | D | 6 | 3 | Incineration or heat recovery | occupational | inhalation | 2 | 3 | 2 | 1 | 8 | 5 |
| Disposal | D | 6 | 4 | Incineration or heat recovery | environmental | direct | 2 | 3 | 2 | 1 | 8 | 5 |
| Product Manufacturing | PM | 2 | 3 | Cleaning out synthesis equipment | environmental | direct | 2 | 1 | 1 | 3 | 7 | 6 |
| Product Manufacturing | PM | 2 | 7 | Accidental spill of CN from synthesis equipment | occupational | inhalation | 2 | 2 | 2 | 1 | 7 | 6 |
| Product Manufacturing | PM | 2 | 8 | Accidental spill of CN from synthesis equipment | occupational | ingestion/dermal/eye | 2 | 2 | 2 | 1 | 7 | 6 |

| | | | | | | | | | | | | |
|---|---|---|---|---|---|---|---|---|---|---|---|---|
| Product Manufacturing | PM | 2 | 9 | Accidental spill of CN from synthesis equipment | environmental | direct | 2 | 2 | 2 | 1 | 7 | 6 |
| Product Application | PA | 3 | 5 | Deposition (and formulation prep) equipment cleanout | environmental | direct | 2 | 2 | 2 | 1 | 7 | 6 |
| Product Application | PA | 3 | 14 | Incidental release of CN from deposition equipment | environmental | direct | 2 | 2 | 1 | 2 | 7 | 6 |
| Product Application | PA | 3 | 15 | Accidental spill of CN from deposition equipment | occupational | inhalation | 2 | 2 | 2 | 1 | 7 | 6 |
| Product Application | PA | 3 | 16 | Accidental spill of CN from deposition equipment | occupational | ingestion/dermal/eye | 2 | 2 | 2 | 1 | 7 | 6 |
| Product Application | PA | 3 | 17 | Accidental spill of CN from deposition equipment | environmental | direct | 2 | 2 | 2 | 1 | 7 | 6 |
| Product Use | PU | 4 | 4 | Misuse: Degraded food packaging | consumer | inhalation | 2 | 3 | 1 | 1 | 7 | 6 |
| Re-use/Recycling | RR | 5 | 7 | Composting used food packaging | consumer | inhalation | 2 | 3 | 1 | 1 | 7 | 6 |
| Re-use/Recycling | RR | 5 | 9 | Composting used food packaging | occupational | inhalation | 2 | 3 | 1 | 1 | 7 | 6 |
| Re-use/Recycling | RR | 5 | 12 | Bio-conversion (anaerobic digestion) | occupational | inhalation | 2 | 3 | 1 | 1 | 7 | 6 |
| Raw Material | RM | 1 | 1 | Harvesting, chipping/shredding, pulping softwood or hardwood | occupational | inhalation | | | | | 0 | 7 |

**Legend:** LCSC, Life-Cycle Stage Code; LCSN, Life-Cycle Stage Number; SN, Scenario Number; ER, Exposure Route; DE, Directness of Exposure; M, Magnitude; L, Likelihood; F, Frequency; RM, raw material; PM, product manufacturing; PA, product application; PU, product use; RR, re-use/recycling; D, disposal; MSW, municipal solid waste; CN, cellulose nanomaterial; CNFs, cellulose nanofibers; CNC, cellulose nanocrystals.

**Table S14.** Exposure scenario ranking for CS3: carboxylated CNF/CNC food additive.

| Life-Cycle Stage | LCSC | LCSN | SN | Scenario | Receptor | ER | DE | M | L | F | Score | Rank |
|---|---|---|---|---|---|---|---|---|---|---|---|---|
| Product Manufacturing | PM | 2 | 10 | Dried formulation extraction and handling (for powder CN ingredients) | occupational | inhalation | 3 | 3 | 3 | 3 | 12 | 1 |
| Product Manufacturing | PM | 2 | 11 | Dried formulation extraction and handling (for powder CN ingredients) | occupational | ingestion/dermal/eye | 3 | 3 | 3 | 3 | 12 | 1 |
| Product Application | PA | 3 | 1 | Transfer CN from synthesis to application facility (e.g. handling, packaging) | occupational | inhalation | 3 | 3 | 3 | 3 | 12 | 1 |
| Product Application | PA | 3 | 2 | Transfer CN from synthesis to application facility (e.g. handling, packaging) | occupational | ingestion/dermal/eye | 3 | 3 | 3 | 3 | 12 | 1 |
| Product Application | PA | 3 | 6 | CN rehydration (if powder CN ingredient) | occupational | inhalation | 3 | 3 | 3 | 3 | 12 | 1 |
| Product Application | PA | 3 | 7 | CN rehydration (if powder CN ingredient) | occupational | ingestion/dermal/eye | 3 | 3 | 3 | 3 | 12 | 1 |
| Product Manufacturing | PM | 2 | 1 | Cleaning out synthesis equipment | occupational | inhalation | 2 | 2 | 3 | 3 | 10 | 2 |
| Product Manufacturing | PM | 2 | 2 | Cleaning out synthesis equipment | occupational | ingestion/dermal/eye | 2 | 2 | 3 | 3 | 10 | 2 |
| Product Application | PA | 3 | 3 | Food manufacturing equipment cleanout | occupational | inhalation | 2 | 2 | 3 | 3 | 10 | 2 |
| Product Application | PA | 3 | 4 | Food manufacturing equipment cleanout | occupational | ingestion/dermal/eye | 2 | 2 | 3 | 3 | 10 | 2 |
| Product Application | PA | 3 | 8 | CN containing food formulation & preparation (e.g. high shear mixing with other ingredients, homogenixing, other) | occupational | inhalation | 2 | 2 | 3 | 3 | 10 | 2 |
| Product Application | PA | 3 | 9 | CN containing food formulation & preparation (e.g. high shear mixing with other ingredients, homogenixing, other) | occupational | ingestion/dermal/eye | 2 | 2 | 3 | 3 | 10 | 2 |
| Product Use | PU | 4 | 1 | Food handling and consumption | consumer | ingestion | 2 | 2 | 3 | 3 | 10 | 2 |
| Product Use | PU | 4 | 2 | Food handling and consumption | consumer | dermal/eye | 2 | 2 | 3 | 3 | 10 | 2 |
| Re-use/Recycling | RR | 5 | 1 | Collection and transport of waste food to re-use or compost facility | occupational | ingestion/dermal/eye | 2 | 2 | 3 | 3 | 10 | 2 |
| Re-use/Recycling | RR | 5 | 2 | Production of food waste into animal feed | environmental | direct | 2 | 2 | 3 | 3 | 10 | 2 |
| Re-use/Recycling | RR | 5 | 7 | Composting CN containing food | environmental | direct | 2 | 2 | 3 | 3 | 10 | 2 |
| Disposal | D | 6 | 5 | Long-term MSW landfill storage | environmental | direct | 2 | 2 | 3 | 3 | 10 | 2 |
| Product Application | PA | 3 | 17 | Packaging and handling of CN-containing food | occupational | ingestion/dermal/eye | 2 | 2 | 2 | 3 | 9 | 3 |
| Re-use/Recycling | RR | 5 | 6 | Composting CN containing food | occupational | dermal/eye | 2 | 2 | 2 | 3 | 9 | 3 |
| Disposal | D | 6 | 1 | Collection and transport to final end-of-life location | occupational | ingestion/dermal/eye | 2 | 2 | 2 | 3 | 9 | 3 |
| Disposal | D | 6 | 2 | Collection and transport to final end-of-life location | environmental | direct | 2 | 2 | 2 | 3 | 9 | 3 |
| Disposal | D | 6 | 6 | Food discarded in uncontrolled environment | environmental | direct | 2 | 2 | 2 | 3 | 9 | 3 |
| Product Manufacturing | PM | 2 | 4 | Incidental release of CN from synthesis equipment | occupational | inhalation | 2 | 2 | 2 | 2 | 8 | 4 |
| Product Manufacturing | PM | 2 | 5 | Incidental release of CN from synthesis equipment | occupational | ingestion/dermal/eye | 2 | 2 | 2 | 2 | 8 | 4 |

| | | | | | | | | | | | | |
|---|---|---|---|---|---|---|---|---|---|---|---|---|
| Product Manufacturing | PM | 2 | 6 | Incidental release of CN from synthesis equipment | environmental | direct | 2 | 2 | 2 | 2 | 8 | 4 |
| Product Application | PA | 3 | 10 | Incidental release of CN from manufacturing equipment | occupational | inhalation | 2 | 2 | 2 | 2 | 8 | 4 |
| Product Application | PA | 3 | 11 | Incidental release of CN from manufacturing equipment | occupational | ingestion/dermal/eye | 2 | 2 | 2 | 2 | 8 | 4 |
| Product Manufacturing | PM | 2 | 3 | Cleaning out synthesis equipment | environmental | direct | 2 | 1 | 1 | 3 | 7 | 5 |
| Product Manufacturing | PM | 2 | 7 | Accidental spill of CN from synthesis equipment | occupational | inhalation | 2 | 2 | 2 | 1 | 7 | 5 |
| Product Manufacturing | PM | 2 | 8 | Accidental spill of CN from synthesis equipment | occupational | ingestion/dermal/eye | 2 | 2 | 2 | 1 | 7 | 5 |
| Product Manufacturing | PM | 2 | 9 | Accidental spill of CN from synthesis equipment | environmental | direct | 2 | 2 | 2 | 1 | 7 | 5 |
| Product Application | PA | 3 | 5 | Food manufacturing equipment cleanout | environmental | direct | 2 | 2 | 2 | 1 | 7 | 5 |
| Product Application | PA | 3 | 12 | Incidental release of CN from manufacturing equipment | environmental | direct | 2 | 2 | 1 | 2 | 7 | 5 |
| Product Application | PA | 3 | 13 | Accidental spill of CN from manufacturing equipment | occupational | inhalation | 2 | 2 | 2 | 1 | 7 | 5 |
| Product Application | PA | 3 | 14 | Accidental spill of CN from manufacturing equipment | occupational | ingestion/dermal/eye | 2 | 2 | 2 | 1 | 7 | 5 |
| Product Application | PA | 3 | 15 | Accidental spill of CN from manufacturing equipment | environmental | direct | 2 | 2 | 2 | 1 | 7 | 5 |
| Re-use/Recycling | RR | 5 | 4 | Composting CN containing food | consumer | ingestion/dermal/eye | 2 | 2 | 2 | 1 | 7 | 5 |
| Re-use/Recycling | RR | 5 | 9 | Bio-conversion (anaerobic digestion) | occupational | ingestion/dermal/eye | 2 | 2 | 2 | 1 | 7 | 5 |
| Re-use/Recycling | RR | 5 | 10 | Bio-conversion (anaerobic digestion) | environmental | direct | 2 | 2 | 2 | 1 | 7 | 5 |
| Disposal | D | 6 | 3 | Incineration or heat recovery | occupational | inhalation | 2 | 2 | 2 | 1 | 7 | 5 |
| Disposal | D | 6 | 4 | Incineration or heat recovery | environmental | direct | 2 | 2 | 2 | 1 | 7 | 5 |
| Product Application | PA | 3 | 16 | Packaging and handling of CN-containing food | occupational | inhalation | 2 | 2 | 1 | 1 | 6 | 6 |
| Re-use/Recycling | RR | 5 | 3 | Composting CN containing food | consumer | inhalation | 2 | 2 | 1 | 1 | 6 | 6 |
| Re-use/Recycling | RR | 5 | 5 | Composting CN containing food | occupational | inhalation | 2 | 2 | 1 | 1 | 6 | 6 |
| Re-use/Recycling | RR | 5 | 8 | Bio-conversion (anaerobic digestion) | occupational | inhalation | 2 | 2 | 1 | 1 | 6 | 6 |
| Raw Material | RM | 1 | 1 | Harvesting, chipping/shredding, pulping softwood or hardwood | occupational | inhalation | | | | | 0 | 7 |

**Legend: LCSC, Life-Cycle Stage Code; LCSN, Life-Cycle Stage Number; SN, Scenario Number; ER, Exposure Route; DE, Directness of Exposure; M, Magnitude; L, Likelihood; F, Frequency; RM, raw material; PM, product manufacturing; PA, product application; PU, product use; RR, re-use/recycling; D, disposal; MSW, municipal solid waste; CN, cellulose nanomaterial; CNFs, cellulose nanofibers; CNC, cellulose nanocrystal.4. Hazard Assessment: SbD Toolbox.**

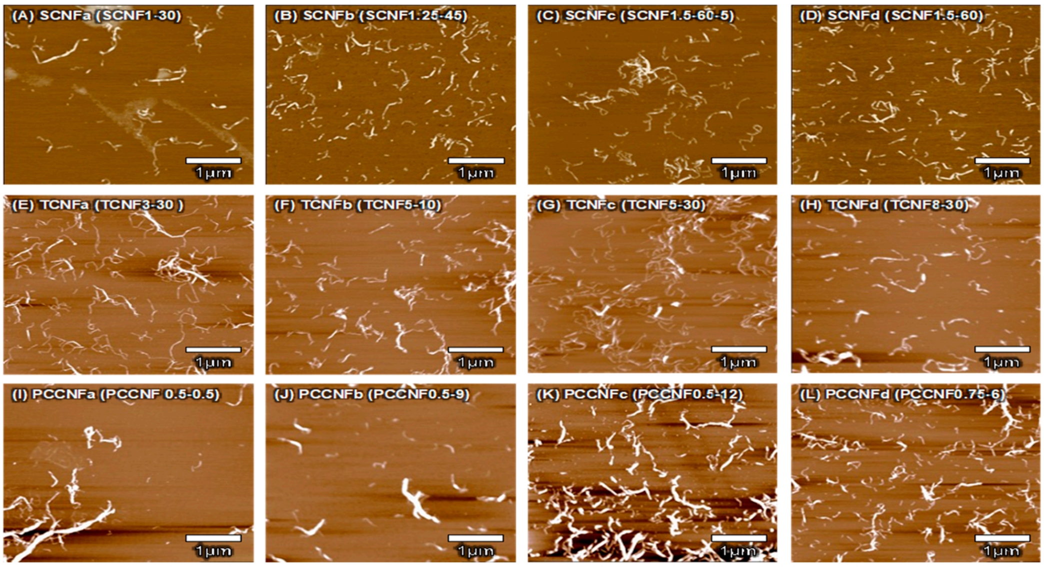


**Figure S1.** Atomic force micrographs of SCNFs (a-d; top row), TCNFs (a-d; middle row) and PCCNFs (a-d; bottom row) designed, synthesized, and characterized for hazard assessment [24]. The micrographs show how differences in manufacturing conditions (concentration, time, blending) resulted in materials that varied by chemistry and morphology.

## 5. Hazard Assessment: Literature Review

*Pulmonary Toxicity of Carboxylated CNs*

Aimonen *et al.* (2022) evaluated the *in vivo* pulmonary effects of TCNF administered to female C57BL/6 mice by repeated pharyngeal aspiration [40]. Three (1-day post-exposure) or six (28- and 90-days post-exposure) mice per group were exposed to three doses (50 µL/mouse) of TCNF at 14, 28, and 56 µg/mouse/aspiration, resulting in accumulative doses of 42, 84, and 168 µg/mouse. An acute inflammatory reaction was induced at the highest dose of TCNF by a statistically significant increase in bronchoalveolar lavage (BAL) fluid neutrophils and BAL eosinophils. At 90 days post-administration, an increase in DNA damage in bronchoalveolar lavage was found.

Catalán *et al.* (2017) evaluated the pulmonary effect of TCNF administered by a single pharyngeal aspiration [41]. Female C57B mice were treated with TCNF (10, 40, 80, or 200 µg/mouse). DNA damage was assessed by the comet assay in BAL and lung cells. Chromosome damage was assessed by the bone marrow erythrocyte micronucleus assay. Inflammation was evaluated by BAL cell counts and analysis of cytokine and histopathological alterations in the lungs. Results showed that TCNF administered by pharyngeal aspiration caused an acute inflammatory response and DNA damage in the lungs, but no systemic genotoxic effect in the bone marrow. Dose-related TCNF accumulation occurred in the bronchi, alveoli, and, to a lesser extent, macrophages.

Ilves *et al.* (2018) also investigated the pulmonary inflammatory effects of carboxylated CNF exposure in mice by oropharyngeal aspiration at 10 or 40 µg/mouse (0.5 or 2 mg/kg bw) [42]. There was a significant induction of inflammatory markers at both doses, suggesting a LOAEL of 0.5 mg/kg bw. Modest immune reactions were also seen after 28 days. However, the effects were markedly attenuated as compared with the ones after 24 h.

Hadrup *et al.* (2019) evaluated the potential for pulmonary inflammation following 24 h and 28 days of exposure of female C57BL/6 mice to 6 or 18 µg of carboxylated CNF by intratracheal instillation [43]. A No Observed Adverse Effect Level (NOAEL) of 0.3 mg/kg bw/day was observed. The lowered BAL neutrophil influx and systemic plasma

SAA3 levels indicated less inflammation and a reduced acute phase response from carboxylated CNF compared to non-functionalized CNF.

Fujita *et al.* (2021) investigated the potential for pulmonary inflammatory response in Sprague-Dawley rats following exposure to a maximum dose of 2.0 mg/kg of TCNFs for 90 days [48]. BALF analysis, histopathological examination, and a comprehensive gene expression analysis confirmed acute inflammation following the instillation of TCNFs. The authors also found that the degree of pulmonary toxicity of TCNF was lower compared with that of multiwalled carbon nanotubes (MWCNTs) and that after 90-days exposure, inflammation had attenuated.

Fujita *et al.* (2022) then exposed rats to TCNF via intratracheal instillation at 0.25, 0.5, or 1.0 mg/kg bw/day for 2 days with an interval of 24 h using 0.5, 1.0, or 2.0 mg/mL aqueous suspension [44]. The researchers found no difference between the proportion of MNPCEs in bone marrow from exposure to TCNF compared to the negative control group. They concluded that the TCNFs studied did not exhibit genotoxicity *in vivo*. The authors also exposed Chinese hamster lung fibroblasts at 25, 50, and 100 μg/mL TCNF for 12 days, in accordance with OECD Test Guideline (TG) 490. They found no increase in chromosomal aberrations or in abnormal chromosomal and polyploid cells. They concluded that the TCNFs studied did not exhibit genotoxicity *in vivo* or *in vitro*.

Ventura *et al.* (2018) observed an increase in chromosome anomalies, but no DNA damage, in human lung epithelial alveolar A549 cells cultured with THP-1 macrophages after treatment with TCNF [45].

*Dermal/Eye Toxicity of Carboxylated CNs*

Nordli *et al.* (2016) assessed the dermal toxicity of TCNF in Human Dermal Fibroblasts and Human Epidermal Keratinocytes [47]. They found no cytotoxicity up to 50 μg/mL, no cytokine induction, and comparable metabolic activity.

No studies were identified that evaluating the potential eye toxicity of carboxylated CNs.

*Oral Toxicity of Carboxylated CNs*

Shimotoyodome *et al.* (2011) evaluated the potential impacts to postprandial blood metabolic variables (*e.g.*, blood glucose, insulin, GIP, and triglyceride levels) in male C57BL/6J mice fed TCNF [46]. TCNF (1.2 mmol/g carboxyl content and 120 aspect ratio) administered with glucose and glyceryl trioleate to mice significantly decreased both plasma insulin and GIP concentrations, especially at 10 min after administration. As the TCNF dose increased from 0 to 0.3 mg/g bw, a more marked decrease in plasma insulin and GIP levels was observed after administration. TCNF (1.2 mmol/g carboxyl content and 120 aspect ratio) exposure significantly reduced the postprandial blood glucose, plasma insulin, GIP, and triglyceride concentrations.

No studies evaluating the chronic oral toxicity of carboxylated CNs were identified.

Table 12 summarizes toxicity benchmark values for carboxylated CNs derived using standardized testing and reported in literature.

*Environmental Hazards of Carboxylated CNs*

Tai *et al.* (2024) investigated the acute toxicity of TCNFs exposed to *D. magna* and *O. latipes* for 48 h at 0, 3.1, 6.3, 12.5, 25, 50, and 100 mg/L, in accordance with OECD TGs 202 and 203 [49]. The researchers found no acute toxicity to the two species at any concentration, resulting in an $E_{50}$ and $LC_{50}$ value of > 100 mg/L.

Tai *et al.* (2024) studied the effects of TCNF exposure on the green alga *R. subcapitata* for 72 h at 0, 3.1, 6.3, 12.5, 25, 50, and 100 mg/L, in accordance with OECD TG

201 [50]. Overall, growth inhibition was not observed at any TCNF concentration, indicating TCNFs exposure does not impact algal growth.

Rusconi *et al.* (2024) performed an acute *in vivo* study with the marine bivalve *M. galloprovincialis* to investigate the uptake and sub-lethal biological responses to CNF and TCNF exposure [51]. Uptake from 96 h of exposure to TCNF (1 μg $L^{-1}$ or 1 mg $L^{-1}$) was evaluated using rhodamine B (RhB) labelled stocks (CNF–RhB and TCNF–RhB). TCNF was found to be taken up by marine mussels but did not result in significant acute toxicity.

Esposito *et al.* (2023) performed embryotoxicity, spermiotoxicity, and ovotoxicity tests by exposing fertilized eggs (zygotes), sperm, or eggs from sea urchin species *P. lividus* and *A. lixula* to TCNF solutions at various concentrations in filtered natural seawater (FNSW) [52]. Neat TCNF exposure affected sea urchin embryo development at the highest tested concentrations, and did not affect fertilization; mitochondrial membrane potential (MMP); intracellular pH and ROS levels; lipid peroxidation (LPO); or sperm motility.

Harper *et al.* (2016) carried out several assays using zebrafish embryos and an *in vivo* developmental toxicity model to assess impacts on growth and reproduction, as well as to assess relative toxicity [53]. Following 5-day continuous exposure to different functionalized CNs, no effects or mortality were observed from TCNF exposure.

*Pulmonary Toxicity of Sulfated CNs*

An acute inhalation toxicity study conducted by O'Connor *et al.* (2014) resulted in no mortality, gross toxicity, adverse effects, or abnormalities in rats after administration of a maximum concentration of 0.26 mg/L of S-CNC for 4 hours [33].

Shvedova *et al.* (2015) assessed the inhalation hazards of S-CNC to mice through pharyngeal aspiration [58]. The exposure involved a dose of 10 μg/mouse administered 2 times a week for 3 weeks. This resulted in impaired pulmonary function, pulmonary inflammation and damage, oxidative stress, increased TGF-β, and elevated collagen levels in lung tissue. Similarly, Yanamala *et al.* (2014) evaluated the pulmonary hazards caused by exposure of C57BL/6 mice to S-CNC (freeze-dried powder or 10 wt% suspension) by pharyngeal aspiration in doses of 50, 100, and 200 μg/mouse [54]. This led to an innate inflammatory response, as evidenced by an increase in the number of leukocytes and eosinophils recovered by BAL.

Endes *et al.* (2014) used a 3D multi-cellular in vitro model of the human epithelial airway barrier to assess the inhalation hazards of S-CNCs [55]. The study involved the aerosolization of well-dispersed and characterized S-CNC suspensions using an Air Liquid Interface Cell Exposure System (ALICE) at realistic, cell-delivered concentrations ranging from 0.14 to 1.57 μg/$cm^2$. Endes *et al.* found no pro-inflammatory responses at the highest concentration of 1.57 μg/$cm^2$ S-CNC. Similarly, Clift *et al.* (2011) investigated the acute inhalation hazard of CNs using a 3D in vitro triple cell coculture model of the human epithelial airway barrier [56]. Aqueous suspensions of S-CNC (5, 15, and 30 mg/L for 24 h) resulted in only a small elevation of pro-inflammatory chemokines at the highest dose examined.

*Dermal/Eye Toxicity of Sulfated CNs*

Following an acute dermal irritation/corrosion test (OECD TG 404), O'Connor *et al.* (2014) observed no corrosive effects from a single dose of S-CNC (0.5 g) to the skin of an albino rabbit [33]. Following a skin sensitization test in guinea pigs (OECD TG 406), S-CNC was found to be nonsensitizing at 1.1 mg/mL (intradermal) and 103 mg/mL (topical). Lastly, following a skin sensitization test in mice (OECD 429), O'Connor *et al.* found S-CNC was not a contact dermal sensitizer at concentrations of < 10.7%.

*Oral Toxicity of Sulfated CNs*

Ede *et al.* (2020) found no adverse effects from oral CNC exposure in rats up to 4% of the diet in a 7, 14, and 90-day subchronic toxicity (OECD 407, 408) test [57]. CNC raises no safety concerns when used as a food ingredient and has a NOAEL > 2,085.3 (males) and 2,682.8 (females) mg/kg.

O'Connor *et al.* (2014) reported no adverse effects from a single-dose oral exposure of CNC to rats (OECD TG 425) and calculated a median lethal dose (LD50) of > 2000 mg/kg bw [33]. Following a 28-day repeated-dose oral exposure of CNC to rats (OECD TG 407), O'Connor *et al.* observed no adverse effects and calculated a NOAEL of > 2000 mg/kg bw.

Table 13 presents a summary of toxicity benchmark values from the literature for sulfated CNs derived using standardized testing.

*Environmental Hazards of Sulfated CNs*

Kovacs *et al.* (2010) evaluated the toxicity of S-CNCs toward various aquatic species [59]. They found that S-CNC have low or no toxicity in zebrafish embryos (*Danio rerio*) at up to 3 g $L^{-1}$ exposure. While S-CNC affected the reproduction of the fathead minnow at a certain concentration, no significant adverse effects on survival and growth in the other species were observed at concentrations below 1 g/L. No toxicity was reported for the freshwater green algae *Pseudokirchneriella subcapitata* (up to 2.8 g $L^{-1}$ of CNC and 1.2 g $L^{-1}$ of CNF) or the microcrustacean *Daphnia sp*. (up to 14 g $L^{-1}$ of CNC and 6 g $L^{-1}$ of CNF), even in prolonged exposure (21-day up to 2.06 g $L^{-1}$). Overall, Kovacs *et al.* found that S-CNC had low toxicity potential and environmental risk.

Harper *et al.* (2016) carried out several assays using zebrafish embryos and an *in vivo* developmental toxicity model to assess impacts on growth and reproduction, as well as to assess relative toxicity [53]. Following 5-day continuous exposure to different functionalized CNs, no effects or mortality were observed from SCNF exposure.
